# Metabolomics-Based Discovery of Small Molecule Biomarkers in Serum Associated with Dengue Virus Infections and Disease Outcomes

**DOI:** 10.1371/journal.pntd.0004449

**Published:** 2016-02-25

**Authors:** Natalia V. Voge, Rushika Perera, Sebabrata Mahapatra, Lionel Gresh, Angel Balmaseda, María A. Loroño-Pino, Amber S. Hopf-Jannasch, John T. Belisle, Eva Harris, Carol D. Blair, Barry J. Beaty

**Affiliations:** 1 Department of Microbiology, Immunology, and Pathology, Colorado State University, Fort Collins, Colorado, United States of America; 2 Sustainable Sciences Institute, Managua, Nicaragua; 3 Laboratorio Nacional de Virología, Centro Nacional de Diagnóstico y Referencia, Ministry of Health, Managua, Nicaragua; 4 Laboratorio de Arbovirología, Centro de Investigaciones Regionales Dr. Hideyo Noguchi, Universidad Autónoma de Yucatán, Mérida, Yucatán, México; 5 Bindley Bioscience Center, Purdue University, West Lafayette, Indiana, United States of America; 6 Division of Infectious Diseases and Vaccinology, School of Public Health, University of California, Berkeley, Berkeley, California, United States of America; Oxford University Clinical Research Unit, VIETNAM

## Abstract

**Background:**

Epidemic dengue fever (DF) and dengue hemorrhagic fever/dengue shock syndrome (DHF/DSS) are overwhelming public health capacity for diagnosis and clinical care of dengue patients throughout the tropical and subtropical world. The ability to predict severe dengue disease outcomes (DHF/DSS) using acute phase clinical specimens would be of enormous value to physicians and health care workers for appropriate triaging of patients for clinical management. Advances in the field of metabolomics and analytic software provide new opportunities to identify host small molecule biomarkers (SMBs) in acute phase clinical specimens that differentiate dengue disease outcomes.

**Methodology/Principal Findings:**

Exploratory metabolomic studies were conducted to characterize the serum metabolome of patients who experienced different dengue disease outcomes. Serum samples from dengue patients from Nicaragua and Mexico were retrospectively obtained, and hydrophilic interaction liquid chromatography (HILIC)-mass spectrometry (MS) identified small molecule metabolites that were associated with and statistically differentiated DHF/DSS, DF, and non-dengue (ND) diagnosis groups. In the Nicaraguan samples, 191 metabolites differentiated DF from ND outcomes and 83 differentiated DHF/DSS and DF outcomes. In the Mexican samples, 306 metabolites differentiated DF from ND and 37 differentiated DHF/DSS and DF outcomes. The structural identities of 13 metabolites were confirmed using tandem mass spectrometry (MS/MS). Metabolomic analysis of serum samples from patients diagnosed as DF who progressed to DHF/DSS identified 65 metabolites that predicted dengue disease outcomes. Differential perturbation of the serum metabolome was demonstrated following infection with different DENV serotypes and following primary and secondary DENV infections.

**Conclusions/Significance:**

These results provide proof-of-concept that a metabolomics approach can be used to identify metabolites or SMBs in serum specimens that are associated with distinct DENV infections and disease outcomes. The differentiating metabolites also provide insights into metabolic pathways and pathogenic and immunologic mechanisms associated with dengue disease severity.

## Author Summary

Epidemics of dengue fever (DF) and dengue hemorrhagic fever/dengue shock syndrome (DHF/DSS) are overwhelming public health capacity for diagnosis and patient care. Developing a panel of biomarkers in acute-phase serum specimens for prognosis of severe dengue disease would be of enormous value for appropriate triaging of patients for management. Metabolomics offers great potential for identification of small molecule biomarkers (SMBs) for diagnosis and prognosis of dengue virus (DENV) infections. We identified metabolites that were associated with and differentiated DHF/DSS, DF and non-dengue (ND) febrile illness outcomes, primary and secondary virus infections, and infections with different DENV serotypes. These metabolites provide insights into metabolic pathways that play roles in DENV infection, replication, and pathogenesis. Some are associated with lipid metabolism and regulation of inflammatory processes controlled by signaling fatty acids and phospholipids, and others with endothelial cell homeostasis and vascular barrier function. Such metabolites and associated metabolic pathways are potentially biologically relevant in DENV pathogenesis. The diagnostic and prognostic efficacy of differentiating metabolites is currently being investigated. Our goal is to identify the most parsimonious SMB biosignature that, when combined with laboratory diagnostic results, eg., DENV NS1 or RNA detection, will provide the most efficient algorithm for dengue diagnosis and prognosis.

## Introduction

Epidemic dengue fever (DF) and dengue hemorrhagic fever/dengue shock syndrome (DHF/DSS) have emerged throughout the tropical and subtropical world with devastating consequences and are overwhelming public health capacity for diagnosis and patient care [1, 2]. Upon presentation early after disease onset, it is clinically impossible to differentiate dengue virus (DENV)-infected patients who will have an unremarkable DF disease episode from those who will progress to potentially fatal DHF/DSS [3–7]. Viral biomarkers that correlate with dengue severity include viremia titer and nonstructural protein 1 (NS1) concentration in the blood, secondary DENV infection, and infection with specific virus genotypes [8–11]. Host biomarkers associated with disease severity include multiple immune molecules, biochemical and physiological response indicators, and genetic polymorphisms [3, 4, 12–21]. Algorithms based upon clinical signs and laboratory test results have been proposed to predict dengue severity [22–28]. However, currently there are no standardized biomarkers or algorithms for prognosis of severe disease outcomes.

Current diagnostic tests and approaches are not meeting the challenges posed by dengue [29, 30]. A paradigm shift in diagnosis/prognosis is essential to address the increasing threat of severe dengue disease. Advances in mass spectrometry, metabolite databases, and analytical software provide exciting new opportunities to identify small molecule biomarkers (SMBs) of dengue disease outcome in acute-phase serum specimens. Mass spectrometry-based metabolomics techniques are being applied with increasing frequency for diagnosis, investigation of pathogenic mechanisms, and monitoring the effects of treatments and interventions of infectious diseases [31–36].

Metabolomics is the analysis of low molecular weight biological molecules that result from metabolic processes. Disease states result in changes in metabolism in cells and systems that affect the profile of metabolites [34]. Analysis of metabolite profiles in disease conditions and comparison with the profiles of non-diseased individuals can be used in diagnosis. Metabolites that differentiate DF and DHF/DSS outcomes could potentially be exploited as SMBs for diagnosis of DENV infections and prognosis of disease severity. Liquid chromatography-tandem mass spectrometry (LC-MS/MS) metabolomics approaches have been used to detect and characterize changing metabolite levels in humans and mosquito vectors that are directly attributable to DENV infection and pathogenesis [32, 37]. Primary DENV infection in humans was shown to cause temporally distinct changes in the serum metabolome, particularly in the lipidome, reflecting the pathogenic mechanisms and metabolic pathways perturbed during the time course of DF [32].

Here, hydrophilic interaction liquid chromatography (HILIC)-MS [38, 39] was used to characterize retrospectively the serum metabolome of patients who were diagnosed as DHF/DSS, DF, or non-dengue (ND) febrile disease, as well as for preliminary characterization of the serum metabolome following infection with two different DENV serotypes and after primary or secondary DENV infection. In this exploratory, proof-of-concept study, metabolites that were associated with and differentiated DF and DHF/DSS, that predicted progression to DHF/DSS in serum of DF patients, and that differentiated infecting DENV serotypes and primary and secondary infections were identified. The differentiating metabolites reflect host responses to the pathogen, including tissue damage, inflammation, and other virus-induced pathology and thus provide insights into fundamental metabolic pathways associated with DENV pathogenesis and potentially novel targets for therapeutic intervention [32, 35]. We have identified candidate SMBs to be evaluated in prospective clinical studies for their diagnostic and prognostic efficacy for DENV infections.

## Methods

### Ethics statement

Serum samples were obtained from collections of sera from patients who had presented with dengue-like febrile disease in Managua, Nicaragua, and Mérida, México. Nicaraguan serum samples had been collected as part of two ongoing pediatric studies being conducted by the University of California, Berkeley, the Nicaraguan Ministry of Health, and the Sustainable Sciences Institute: the Pediatric Dengue Cohort Study, which is focused upon studying transmission of DENV and identifying immune correlates of protection, and the Hospital-based Dengue Study, which is focused on studying clinical, immunological, and viral risk factors for severe DENV infections. Parents or legal guardians of participants provided written informed consent, participants 6 years of age and older provided assent, and participants in the Hospital-based Dengue Study 12 years of age and older provided written assent. These studies were approved by the UC Berkeley Committee for the Protection of Human Subjects (Protocols # 2010-06-1649 and 2010-09-2245) and the IRB of the Nicaraguan Ministry of Health. Mexican samples had been collected in the Laboratorio de Arbovirología, Centro de Investigaciones Regionales Dr. Hideyo Noguchi or the Unidad Universitaria de Inserción Social (UUIS) San José Tecoh, both from the Universidad Autónoma de Yucatán (UADY), Mérida, Yucatán, México, from patients who were referred by a primary-care physician for diagnostic testing. These samples were procured as part of the normal dengue diagnosis mission of the laboratory and not as part of an experimental protocol. These samples provided an opportunity to determine if metabolites detected in Nicaraguan patients that differentiated dengue disease outcomes could also be detected in dengue patients with different genetic, environmental, and demographic backgrounds. This research was approved by the Bioethics Committee of the Centro de Investigaciones Regionales “Dr. Hideyo Noguchi” (CIR) of the Universidad Autónoma de Yucatán and reviewed by the CSU Institutional Review Board and considered to be an exempt project (Category 4). A portion of serum samples from Nicaraguan and Mexican patients who were diagnosed as DHF/DSS, DF, or ND were de-identified and sent to CSU for metabolomics analysis.

### Serum samples

In Nicaragua, 88 serum samples were retrospectively obtained from patients who had been diagnosed as DHF/DSS, DF, or ND (Table 1). The serum sample collection dates for the DF patients ranged from days 1 to 6 of illness and for the DHF/DSS patients from days 3 to 6 of illness. These patients had presented to the study clinic Centro de Salud Sócrates Flores Vivas and met the 1997 WHO case definition for dengue [30] or presented with undifferentiated fever, or to the Hospital Infantil Manuel de Jesús Rivera, the National Pediatric Reference Hospital, with a fever or history of fever <7 days and one or more of the following signs and symptoms: headache, arthralgia, myalgia, retro-orbital pain, positive tourniquet test, petechiae, or signs of bleeding. In Nicaragua, all samples were from pediatric patients <15 years of age, and 50% of the samples were from male and 50% from female patients. Fifty-nine positive samples were included in the analysis, and all were from patients infected with DENV-2. Of the 59 positive samples, 18 (30%) were primary infections and 41 (69%) were secondary infections (Table 1). The majority of both DF and DHF/DSS patients experienced secondary infections.

**Table 1:** Serum specimens from Nicaraguan (A) patients analyzed in mass spectrometry studies.

Nicaraguan samples (N = 88, 100%)								
Clinical Diagnosis		Day of illness		Sex		Serotype	Infection	
	N	Mean	Range	Female	Male	DENV-2	Primary	Secondary
**DF**	29 (32.95%)	4.2	1 to 6	13 (14.77%)	16 (18.18%)	29 (32.95%)	9 (10.22%)	20 (22.72%)
**DHF/DSS**	30 (34.09%)	4	3 to 6	16 (18.18%)	14 (15.90%)	30 (34.09%)	9 (10.22%)	21 (23.86%)
**ND**	29 (32.95%)	3.2	2 to 5	15 (17.04%)	14 (15.90%)	NA	NA	NA

Abbreviations: DF—dengue fever; DHF/DSS-dengue hemorrhagic fever/shock syndrome; ND—non-dengue; N- total number of samples; U- unknown; DENV-dengue virus; NA–Not applicable.

In México, 101 serum samples were retrospectively obtained from patients who were referred to the UADY clinics and had been diagnosed as DHF/DSS, DF, or ND (Table 2). The sample collection dates for the DF patients ranged from days 2 to 5 of illness, and for the DHF/DSS patients, from days 2 to 6 of illness. Approximately 10% (11) of these samples were from pediatric patients (1–15 years old) and 90% (90) from adult patients (ages 16–71 years); 53% were from female and 47% from male patients. Sixty-eight DENV-positive samples were included in the analysis. In 47 samples (69%), the infecting DENV serotype was determined: 25 patients were infected with DENV-1 (37%), and 22 (32%) were infected with DENV-2. For patients infected with DENV-1, 15 patients were diagnosed as DF and 10 as DHF/DSS. For patients infected with DENV-2, 15 were diagnosed as DF and 7 as DHF/DSS (Table 2). In 21 patients (31%), the infecting virus serotype was not determined (Table 2). For most of the Mexican patients, information on whether the patients experienced a primary or secondary infection was not available, as these clinical specimens were not collected as part of an experimental protocol.

**Table 2:** Serum specimens from Mexican patients analyzed in mass spectrometry studies.

Mexican samples (N = 101, 100%)											
Clinical Diagnosis		Day of illness		Sex		DENV Serotype			Infection		
	N	Mean	Range	Female	Male	1	2	Unk	Primary	Secondary	Unk
**DF**	42 (41.85%)	2.7	2 to 5	21 (20.79%)	21 (20.79%)	15 (14.85%)	15 (14.85%)	12 (11.88%)	2 (1.98%)	1 (0.99%)	39 (38.61%)
**DHF/DSS**	26 (25.74%)	3.8	2 to 6	14 (13.86%)	12 (11.88%)	10 (9.90%)	7 (6.93%)	9 (8.91%)	0	4 (3.96%)	22 (21.78%)
**ND**	33 (32.67%)	3.5	2 to 6	17 (16.83%)	16 (15.84%)	NA	NA	NA	NA	NA	NA

Abbreviations: DF—dengue fever; DHF/DSS—dengue hemorrhagic fever/dengue shock syndrome; ND—non-dengue; N- total number of samples; Unk—unknown; DENV—dengue virus; NA—Not applicable

Serum samples from patients in Nicaragua and Mexico were frozen at -80°C until thawed prior to preparation for LC-MS.

In Nicaragua, a case was considered laboratory-confirmed dengue when acute DENV infection was demonstrated by detection of DENV RNA by RT-PCR, isolation of DENV, seroconversion of DENV-specific IgM antibody titers observed by MAC-ELISA in paired acute- and convalescent-phase samples, and/or a ≥4-fold increase in anti-DENV antibody titer measured using inhibition ELISA in paired acute and convalescent samples. In Nicaragua, computerized algorithms based on the 1997 WHO schema were used to classify cases according to disease severity [7, 30, 40]. In Mexico, a case was considered laboratory-confirmed dengue by detection of DENV RNA by RT-PCR, isolation of DENV, or detection of DENV-specific IgM antibodies. Classification of dengue severity was based on the traditional 1997 WHO definitions [7, 30, 41]. The final diagnosis of DHF/DSS, DF, or ND febrile illness based upon clinical and laboratory test results was available for each patient.

In the Nicaraguan hospital study, a medical history was taken upon enrollment, and a complete physical exam was performed. Clinical data were collected every 12 hours for inpatients and every 24 hours for outpatients on Case Report Forms (CRFs) to follow patients’ disease evolution, with vital signs and fluid intake/output recorded more often as appropriate. In the Pediatric Dengue Cohort Study, febrile illnesses that met the WHO criteria for suspected dengue and undifferentiated febrile illnesses were treated as possible dengue cases and followed during the acute phase of illness by study physicians at the clinic. Cases were monitored closely for severe manifestations and were transferred by study personnel to the Infectious Disease Ward of the Hospital Infantil Manuel de Jesús Rivera when they presented with any sign of alarm [42, 43].

### Serum sample preparation

For LC-MS analysis, serum samples from Nicaraguan and Mexican patients were thawed on ice, and 25 μl of serum was added to cold LC-MS grade methanol (final concentration 75%) [33]. The extract was dried using a speed vacuum at room temperature, suspended in 25 μl of 100% LC-MS grade acetonitrile (ACN), and incubated at room temperature for 10 minutes (min). Following vortexing for 1 min and centrifuging for 5 min at 4°C at 14,000 rpm, 15 μl of the supernatant was transferred to a glass vial for LC-MS analysis [38, 39]. Biological samples were randomized, and the clinical diagnosis was not considered during sample preparation and data collection.

Abundance measures of MFs produced by LC-MS metabolomics analyses should be considered semi-quantitative in discovery-phase studies [36] and are influenced by instrument and technical variation. To address this, quality control (QC) for SMB measurement followed the recommendations of Dunn et al. [44] for LC-MS analysis of human biofluids. Specifically, human serum (Sigma) was purchased and aliquoted. Each aliquot was processed using the protocol for preparation of human serum samples. After drying, the aliquot was frozen at -80°C until analyzed. For each experimental analysis, the QC sample and the dried experimental samples were reconstituted at the same time. The QC sample was analyzed first by LC-MS and the results compared to QC results obtained in previous analyses. Comparisons included the number of MFs detected, abundance of the MFs, and the baseline of the total ion chromatogram (TIC) among previously analyzed runs. In addition, the QC sample was analyzed after every 15 clinical samples. If differences were detected between QC control results (either previous results or within the analysis), the analysis was stopped, the ionization source and the column (see below) were cleaned, and the mass spectrometer recalibrated. Additional samples were not analyzed until the QC analyses were satisfactory. A reference solution containing ions with *m/z* (mass-to-charge ratio) values 121.050873 and 922.009798 was infused directly with a capillary pump to ensure mass accuracy; the mass spectrometer continually was normalized to the intensity of these two ions.

To evaluate the reproducibility of the LC-MS analysis, the retention time (RT), and the area under the peaks of ten randomly selected representative metabolites were determined using the Nicaraguan serum specimens (N = 88). All differences in RTs and *m/z* values were ≤0.25 min and 15 ppm, respectively. All relative standard deviations (RSD) of the peak areas was below 25% (Table 3), confirming acceptable reproducibility of the chromatographic separation and accuracy of the mass measurements.

**Table 3:** Reproducibility of peak measurements in QC samples.

No.	*m/z*	RT (minutes) (±SD)*	RSD** (100%) Peak areas
1	664.50	1.021 (±0.267)	14.3%
2	682.36	1.05 (±0.002)	23.09%
3	757.35	1.06 (±0.008)	17.1%
4	375.18	2.32 (±0.011)	8.4%
5	456.32	2.34 (±0.129)	11.5%
6	326.25	2.34 (±0.064)	20.6%
7	430.75	12.11 (±0.035)	8.1%
8	884.56	12.07 (±0.105)	25.4%
9	902.55	11.96 (±0.124)	20.5%
10	116.07	16.41 (±0.118)	13.0%

*S.D—standard deviation

** RSD–relative standard deviation

### HILIC-MS analyses of serum specimens

Analyses of the prepared serum samples were performed using an Agilent 1200 series high performance liquid chromatography (HPLC) system connected to an Agilent 6520 Quadrupole Time-of-Flight (Q-TOF) MS fitted with a dual electrospray ionization (ESI) source (Agilent Technologies, Palo Alto, CA). Metabolites were separated using a Cogent hydrophilic type-C silica diamond-hydride column (particle size 4μm, pore size 100 Å, 2.1 mm x 150 mm) with a Cogent diamond hydride guard column (size 2.0 mm x 20 mm) (Microsolv Technology Corporation, NJ) [45, 46]. A 5-μl aliquot of each processed serum sample was applied to the column that had been equilibrated with 5% solvent A (0.1% formic acid in H_2_O) and 95% solvent B (0.1% formic acid in ACN). Metabolites were eluted with the following nonlinear gradient formed with solvents A and B at a flow rate of 0.4 ml/min: 0.2 to 30 min, 95–50% B; 30 to 35 min, hold at 50% B; 35 to 40 min 50–20% B; 40 to 45 min 20–95% B.

HPLC column eluent was directly introduced into the Q-TOF instrument for metabolite detection. The MS parameters used were as follows: scan rate: 1.4 spectra/sec; Vcap: 4000V; drying gas (N_2_): 325°C at 10 l/min; nebulizer pressure: 45 psi fragmentor: 150 V; skimmer: 65V; octopole RF peak: 750 V and 2 GHz extended dynamic range mode; mass range: 100–1700 Da. Reference solution containing ions with *m/z* (mass-to-charge ratio) values of 121.050873 and 922.009798 was infused directly with a capillary pump to ensure mass accuracy. Mass spectra data were collected in both centroid and profile modes.

### Data analysis

HILIC-MS data were analyzed using Agilent’s MassHunter Qualitative Analysis version B.05 software to detect molecular features (MFs) (compounds with defined accurate mass and RT) present in each sample with a minimum abundance of 600 counts, ion species H+, charge state maximum 1, compound ion count threshold 2 or more ions, and all other parameters left at default values.

MFs from the dengue diagnosis groups (DHF/DSS, DF, and ND) were compared using Agilent’s Mass Profiler Professional (MPP), version B.12.01. MFs were aligned with 0.2 min retention time and 15 ppm mass tolerance and filtered based on their presence in at least 50% of samples in at least one diagnosis group. Subsequently, MFs were baselined to the median of all samples and normalized to the 75^th^ percentile shift. The relative abundance of each filtered MF was then compared pairwise between diagnosis groups using ANOVA and Student's t-test. For all comparisons, the false discovery rate was calculated using the Benjamini-Hochberg algorithm, and the fold change (FC) was calculated for metabolites with corrected p-values of <0.05. MFs with a corrected p-value of <0.05 and FC of >2 (positive or negative) were identified *in silico* when possible by interrogating the neutral mass of each in online databases [36].

The metabolites were putatively identified using Metlin [47], HMDB [48], or the Omics discovery pipeline [49]. Metlin parameters used for identification were neutral charge and mass tolerance of ± 10 ppm. HMDB parameters used were ion mode: positive, adduct type M+H, and molecular weight tolerance ± 0.01 Da. The Omics pipeline parameters used for identification included charge 0 and mass tolerance 0.01 Da. The number of database hits or other possible identities presented in S1 and S2 Tables were obtained using Metlin.

All of the MFs that statistically differentiated the DHF/DSS, DF and ND disease outcomes are listed in S1 and S2 Tables (Nicaraguan and Mexican serum differentiating metabolites, respectively). The chemical formulas were calculated using ChemCalc (Institute of Chemical Sciences and Engineering) [50]. The metabolites are listed by the Metabolomics Standard Initiative (MSI) level of identification [51, 52].

MSI level 1: Identified metabolites (experimental data matched chemical reference standards acquired on the same analytical platform).

MSI level 2: Identified metabolites (without chemical reference standards, based on physicochemical properties and spectrum similarity with public/commercial spectrum libraries).

MSI level 3: Putatively identified metabolites (based on physicochemical characteristics of a chemical class of compounds or by spectrum similarity to known compounds of a chemical class).

MSI level 4: Unidentified metabolite (unidentified or unclassified MFs that still could be differentiated or quantified based on spectrum data). These MFs could not be identified using the databases and Omics discovery pipeline [52].

### Structural identification of metabolites by HILIC-MS/MS

Based upon their potential biological relevance, selected *in silico*-identified metabolites were further analyzed by targeted liquid chromatography-tandem mass spectrometry (LC-MS/MS) to corroborate their identities. When available, commercial standards were purchased, and the MS/MS spectra of the standard and the candidate SMB in the native sample were compared. If a commercial standard was not available for the *in silico*-identified compound, the spectrum obtained from LC-MS/MS analysis of the native sample was compared to spectra available in the NIST commercial library [53]. Many metabolites that differentiated the disease outcomes with strong p-values and FCs remain to be identified at MSI levels 1 and 2 (S2 and S3 Tables). This is due in part to the lack of commercially available standards and to lack of appropriate spectra (e.g., spectra obtained using same platforms and parameters) in the commercially available libraries.

### Identification of vitamin D_3_ isotypes using multiple reaction monitoring (MRM) LC-MS/MS

A targeted analysis of a subset of 15 samples was utilized to validate the identification of vitamin D_3_ isotypes [54, 55]. To each sample, 10 ng of [^2^H]_3_-25-hydroxyvitamin D_3_, 10 ng of [^2^H]_6_−1,25-dihydroxyvitamin D_3_, and 100 ng of [^2^H]_6_-vitamin D_3_ internal standard were added, followed by 1 ml of cold (-20°C) acetone. Each sample was vortexed and centrifuged to precipitate protein. The supernatant was dried using a rotary evaporation device. Just before analysis, each sample was derivatized by adding 50 μl 4-phenyl-1,2,4-triazoline-3,5-dione (PTAD) solution (1 mg/ml of ACN) to each dry sample and reacting for 1 hour at room temperature.

Samples were immediately analyzed by LC-MS/MS (Agilent 6460 QQQ coupled to Rapid Resolution 1200 LC system; Agilent Technologies, Santa Clara, CA). Vitamin D_3_ concentrations were determined with [^2^H]_6_-vitamin D_3_ internal standard; 25-hydroxyvitamin D_3_ concentrations were determined with [^2^H]_3_-25-hydroxyvitamin D_3_- internal standard; 1,25-dihydroxyvitamin D_3_ concentrations were determined with [^2^H]_6_−1,25-dihydroxyvitamin D_3_ internal standard. An Agilent Zorbax C18 2.1x50 mm column was used for analysis. Buffers A and B consisted of 0.1% formic acid + 0.1% methylamine and ACN + 0.1% formic acid + 0.1% methylamine. All data were acquired in MRM mode by monitoring the methylamine adducts [54, 55]. The transitions that were monitored by MRM for the identification of vitamin D_3_ isotypes and the collision energy used for fragmenting each MF are shown in Table 4. Two transitions (a deuterated and a non-deuterated) were monitored for each compound [55].

**Table 4:** Multiple reaction monitoring transitions used during LC-MS/MS analysis.

Metabolite	Mass transition (m/z)	Collision Energy (V)
[^2^H]_6_−1,25-OH Vitamin D_3_	629.3 →314.1	15
1,25-OH Vitamin D_3_	623.3→314.1	15
[^2^H]_3_-25-OH Vitamin D_3_	610.3→301.1	10
25-OH Vitamin D_3_	607.3→298.1	10
[^2^H]_6_-Vitamin D_3_	597.3→298.1	10
Vitamin D_3_	591.3→298.1	10

## Results

### Global metabolomic changes in sera of DF and DHF/DSS patients

**Fig 1:**
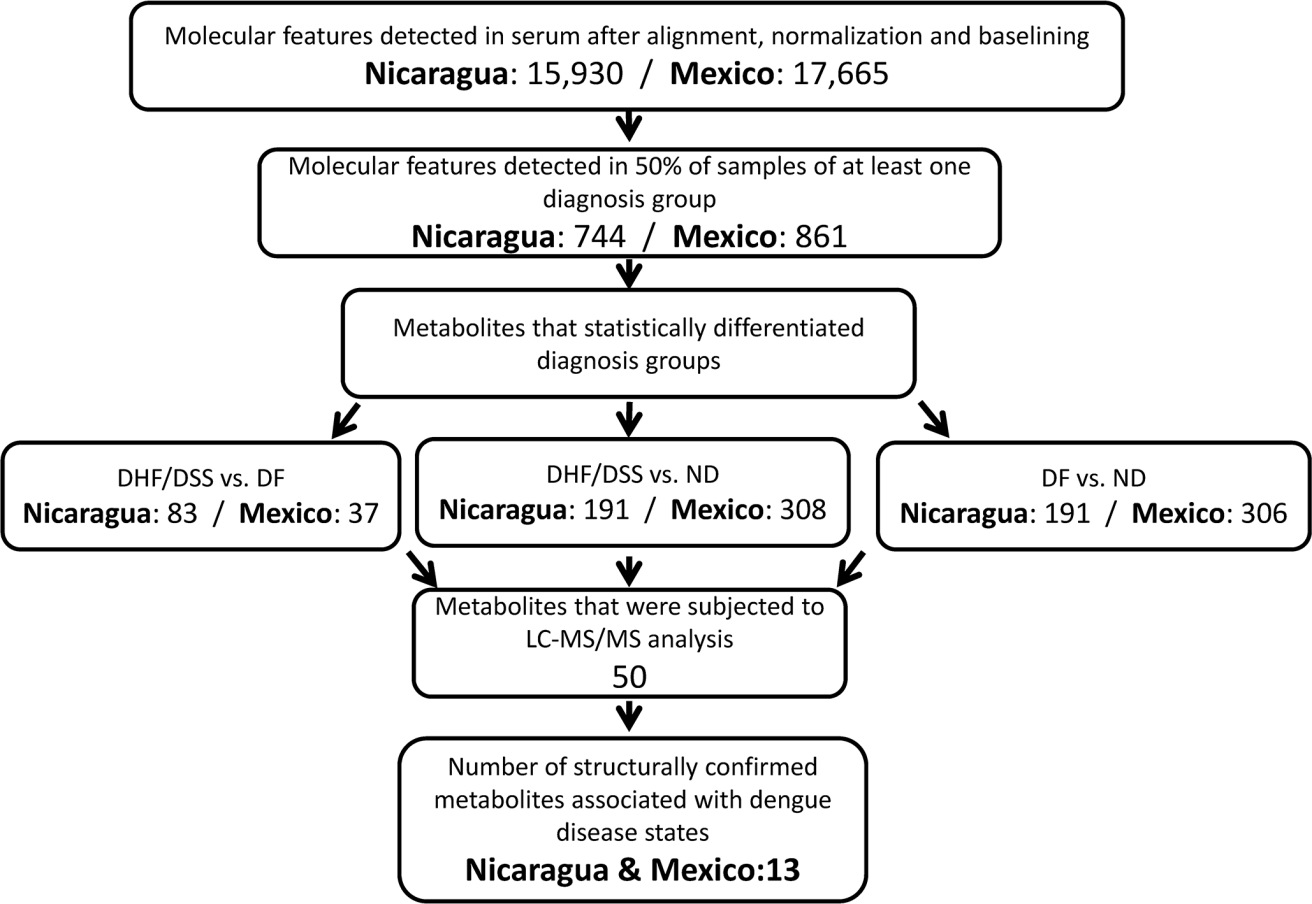
Flow chart of hydrophilic interaction liquid chromatography (HILIC)-MS analyses of molecular features (MFs) in serum specimens of Nicaraguan and Mexican dengue patients. Results are based upon pairwise comparison of MFs between diagnosis groups using cut-offs of corrected p-value <0.05, FC >2.

Characterization of metabolites in sera by HILIC-MS revealed 15,930 MFs in Nicaraguan specimens and 17,665 MFs in Mexican specimens (Fig 1). These were further analyzed using Mass Profiler Professional (MPP) to select MFs present in at least 50% of samples of at least one diagnosis group. This yielded 744 MFs in Nicaraguan serum specimens and 861 in Mexican samples (Fig 1).

### Principal component analyses reveals clustering of samples based on dengue disease diagnosis groups in Nicaraguan samples

**Fig 2:**
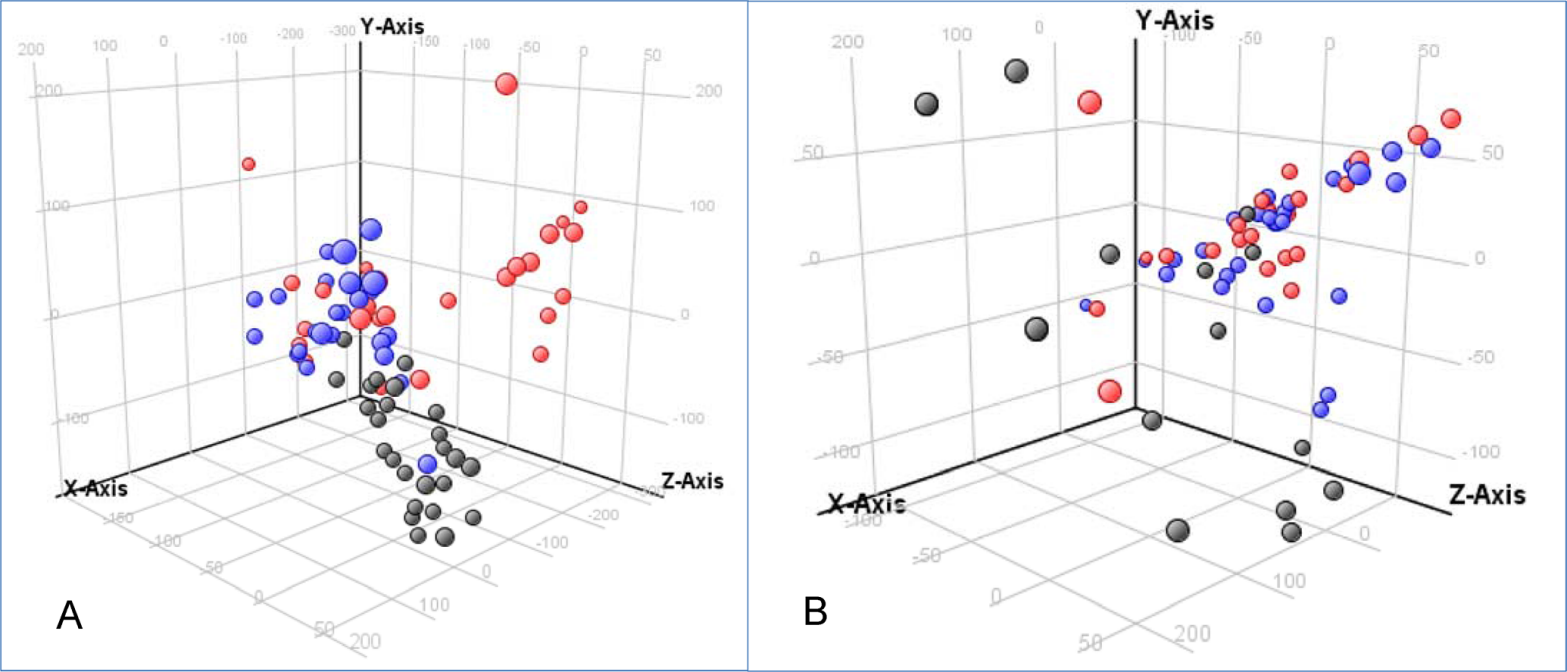
**PCA of Nicaraguan (A) and Mexican (B) HILIC-MS serum metabolomics data.** Red spheres represent patients diagnosed as DHF/DSS; blue spheres represent patients diagnosed as DF; black spheres represent patients with non-dengue febrile illness. The percentage of variation found in A is: X axis 25.4%, Y axis 16%, Z axis 5.8%. The percentage of variation found in B is: X axis 18.39%, Y axis 9.82%, Z axis 8.02%

PCA plots demonstrated clustering of specimens by dengue diagnosis group in Nicaraguan samples, notably of the DHF/DSS patients (Fig 2A). In contrast, there was little evidence of clustering by diagnosis group in the Mexican samples (Fig 2B). Potential factors, such as age, gender, or infecting serotype that could contribute to the lack of clustering in the Mexican samples will be addressed below.

### Principal component analysis reveals clustering of samples stratified by infecting virus serotype and by primary versus secondary DENV infection

Many factors are known to condition dengue disease severity, including primary versus secondary infection and infecting virus serotype and genotype. PCA was used to investigate the role of these potentially confounding factors on the serum metabolome of dengue patients.

#### DENV serotype

**Fig 3:**
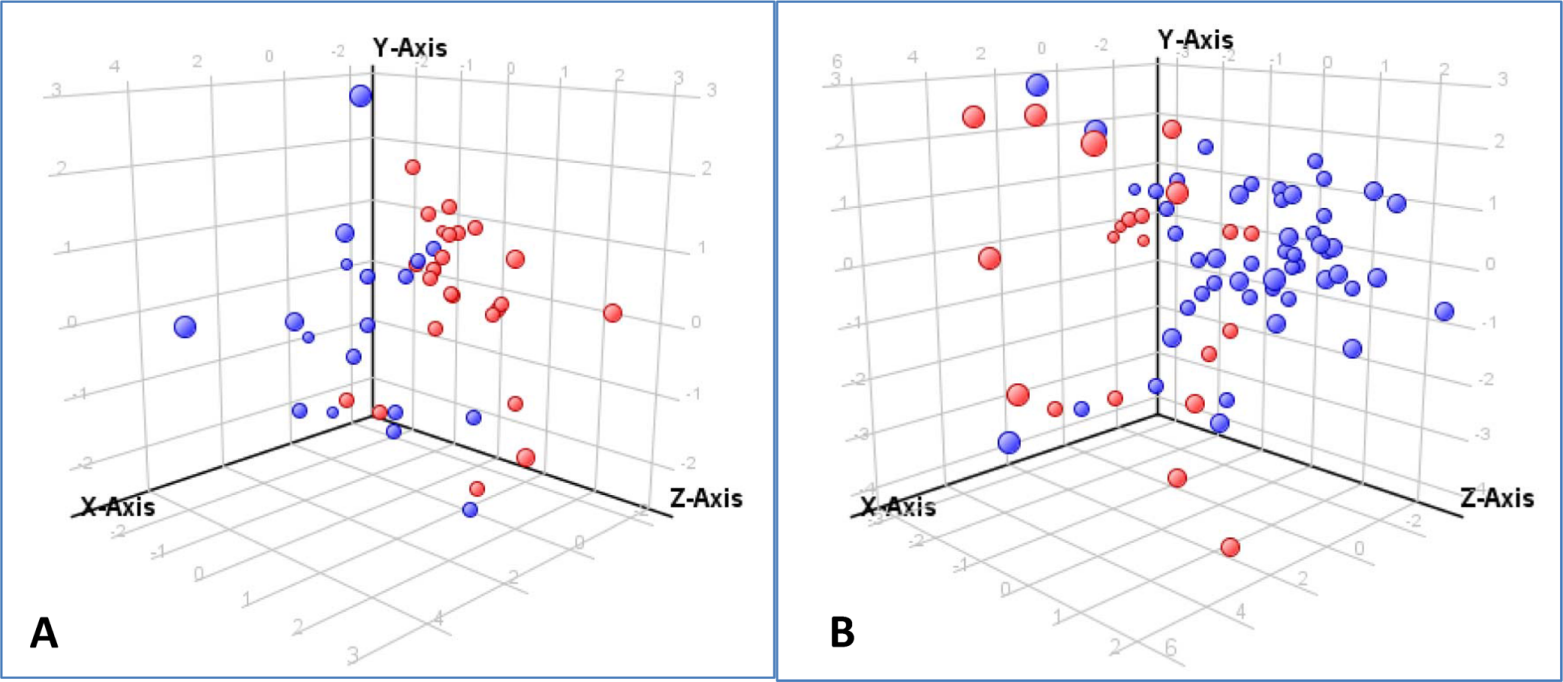
**Principal component analysis reveals clustering of (A) Mexican serum samples by infecting DENV serotype and (B) Nicaraguan serum samples by primary or secondary infection.** In A, red spheres represent patients infected with DENV-1, and blue spheres represent patients infected with DENV-2. The percentage of variation found is: X axis 19.65%, Y axis 12.43%, Z axis 10.09%. In B, red spheres represent patients with primary-infection; blue spheres represent patients with secondary infection. The percentage of variation found is: X axis 31.75%, Y axis 10.72%, Z axis 8.83%

All of the Nicaraguan dengue patients (DF and DHF/DSS) included in this analysis had been infected with DENV-2 (Table 1). Thus we could not compare the effect of infecting serotype on the metabolome in these patients. The Mexican patients had been infected either with DENV-1 or DENV-2 (Table 2), which provided an opportunity to investigate the role of DENV serotype in perturbation of the serum metabolome. All of the DF and DHF/DSS serum samples were stratified by infecting DENV serotype and characterized by PCA (Fig 3A).

Clustering of DENV-1 and DENV-2 infections was demonstrated in the PCA plot (Fig 3A). Statistical analysis revealed 33 MFs present in 100% of one group (either DENV-1 or DENV-2 infections) and 429 MFs were present in at least 50% of the samples of either serogroup. Pairwise comparisons of the 429 MFs, using the same statistical approach and cut-off values used for the dengue diagnosis/prognosis analysis, yielded 25 metabolites that statistically differentiated DENV-1 from DENV-2 infections. Differentiating metabolites include 13 metabolites identified at MSI level 3, predominantly lipids, and 12 metabolites identified at MSI level 4 (S4 Table).

#### Primary versus secondary infection

As noted previously, the Mexican samples were procured as part of the normal diagnostic mission of the laboratories in Merida, Mexico, and unfortunately, little information was available concerning whether the patient experienced a primary or secondary DENV infection (Table 2). In contrast, the Nicaraguan specimens were collected as part of a study protocol, and the presence of a primary or secondary immune response was determined (Table 1). To evaluate the potential role of primary or secondary DENV infection on the serum metabolome, Nicaraguan patient specimens were stratified by primary or secondary infections and the serum metabolomes were characterized by PCA (Fig 3B). Clustering of samples by primary and secondary infections was detected. Statistical analysis revealed 76 MFs in in at least 100% of one group and 610 MFs were present in 50% of the samples of either infection group. Pairwise comparisons of these 610 MFs, using the same statistical approach and cut-off values used for the previous dengue diagnosis analysis, yielded 30 metabolites that differentiated patients with primary or secondary infections. Differentiating metabolites included 8 MFs identified at MSI level 3 and 22 MFs at MSI level 4 (S5 Table). None of the 13 metabolites listed in Table 5 that differentiated the dengue diagnosis groups were detected in this analysis.

**Table 5:** Differentiating serum metabolites* with identities confirmed by HILIC or MRM tandem mass spectrometry**.

Serum				Nicaragua						Mexico						MSI
				DHF/DSS-DF**		DHF/DSS-ND		DF-ND		DHF/DSS-DF		DHF/DSS- ND		DF-ND		Level
Accurate mass	RT**	Metabolite	Chemical formula	P value	FC**	P value	FC	P value	FC	P value	FC**	P value	FC	P value	FC	***
**Amino acid**																
115.0635	14.24	Proline	C_5_H_9_NO_2_	>0.05	**-2.15**	**6.65E-05**	**-8.88**	**5.37E-03**	**-6.74**	>0.05	<2	>0.05	<2	>0.05	<2	1
**Unsaturated fatty acids**																
226.1932 ***	1.33	Myristoleic acid	C_14_H_26_O_2_	**3.21E-03**	**-5.06**	**7.37E-03**	**-4.38**	**4.84E-02**	<2	>0.05	<2	**5.18E-02**	**-4.68**	**1.06E-02**	**-5.64**	2
278.2245	1.10	α–Linolenic acid	C_18_H_30_O_2_	**1.20E-06**	**10.94**	**7.82E-06**	**10.01**	>0.05	<2	>0.05	<2	**5.04E-04**	**-8.61**	**3.32E-03**	**-7.27**	1
302.2243	1.10	Arachidonic acid	C_20_H_30_O_2_	**7.49E-03**	**6.49**	**4.18E-02**	**4.98**	>0.05	<2	**8.09E-03**	**3.75**	7.87E-02	<2	**2.93E-02**	**-2.10**	1
328.2402	1.15	Docosahexaenoic acid	C_22_H_32_O_2_	**1.98E-04**	**7.44**	**8.39E-05**	**7.61**	>0.05	<2	**5.85E-04**	**-9.6**	**4.18E-02**	**-2.00**	**4.11E-03**	**2.00**	1
**Vitamin D**																
384.3387	1.15	Vitamin D_3_	C_27_H_44_O	>0.05	<2	>0.05	<2	>0.05	<2	>0.05	<2	**6.35E-03**	**-6.53**	**2.27E-02**	**-5.02**	1
400.3339	1.14	25-hydroxyvitamin D_3_	C_27_H_44_O_2_	>0.05	**4.27**	>0.05	**-3.76**	**9.91E-05**	**-8.03**	>0.05	<2	**2.16E-03**	**-7.64**	**1.25E-02**	**-5.79**	1
416.3282	1.31	1,25-dihydroxyvitamin D_3_	C_27_ H_44_O_3_	**5.33E-03**	**-5.03**	**7.35E-03**	**7.95**	>0.05	<2	>0.05	<2	**2.52E-04**	**-8.30**	**3.06E-04**	**-8.08**	1
**Phospholipids**																
495.3324	14.97	Lysophosphatidylcholine (16:0)	C_24_H_50_NO_7_P	**9.29E-03**	**7.55**	**4.97E-03**	**7.95**	>0.05	<2	>0.05	<2	**2.42E-04**	**11.69**	**1.22E-03**	**10.07**	1
521.3481	13.90	Lysophosphatidylcholine (18:1)	C_26_H_52_NO_7_P	**1.02E-03**	**9.16**	**4.03E-04**	**9.50**	>0.05	<2	>0.05	<2	**2.47E-04**	**9.60**	**1.24E-03**	**8.49**	1
759.5778 ****	12.25	Phosphatidylcholine (34:1)	C_42_H_82_NO_8_P	**6.82E-02**	**3.76**	**8.63E-04**	**-7.46**	**1.16E-07**	**-11.22**	**5.47E-02**	**2.56**	**5.90E-04**	**-13.35**	**3.12E-04**	**-13.65**	2
761.5934 ****	11.88	Phosphatidylcholine (34:0)	C_42_H_84_NO_8_P	**9.82E-02**	**3.92**	**1.32E-02**	**-5.92**	**2.28E-05**	**-9.84**	>0.05	<2	**1.01E-02**	**-9.87**	**3.65E-03**	**-6.13**	2
771.5415	12.22	Phosphatidylcholine (36:1)	C_44_H_86_NO_7_P	**3.70E-03**	**6.90**	**8.95E02**	**-4.25**	**2.27E-06**	**-11.15**	>0.05	<2	>0.05	<2	>0.05	**2.18**	2

*Metabolites that statistically differentiated (P<0.05, FC >2) dengue outcomes in at least one of the pairwise comparisons of DHF/DSS, DF, and ND diagnosis groups.

**Abbreviations: HILIC- hydrophilic interaction chromatography or MRM—multiple reaction monitoring tandem mass spectrometry (LC-MS/MS); DHF/DSS—dengue hemorrhagic fever/ dengue shock syndrome; DF—dengue fever; ND- non-dengue febrile disease; RT—retention time; FC—fold change. Bolded values indicate statistically significant differences in pairwise comparisons of the two diagnosis groups.

*** MSI—Metabolomics Standard Initiative: level 1- identity verified with a commercial standard, level 2- identity verified using National Institute of Standards and Technology (NIST) commercial library.

****Ionized form of certain metabolites: myristoleic acid [M+H-H_2_O]^+^ = 226.1932, phosphatidylcholine (34:1) [M+Na-2H]^+^ = 781.5627, phosphatidylcholine (34:0) [M+H-Na]^+^ = 784.5827. All other metabolites listed were [M+H]^+^.

Infection by two DENV serotypes and differential proportions of primary and secondary infections could have contributed to the lack of clustering of Mexican patients by dengue diagnosis groups (Fig 2B).

### Identification of metabolites that differentiate dengue diagnosis groups

Pairwise comparisons of abundances revealed MFs in acute phase serum specimens that statistically (corrected p-value <0.05, FC >2) differentiated the DHF/DSS, DF, and ND diagnosis groups. In Nicaraguan specimens, 83 MFs differentiated DHF/DSS from DF patients, 191 MFs differentiated DHF/DSS from ND patients, and 191 MFs differentiated DF from ND patients (Fig 1). In Mexican serum specimens, 36 MFs differentiated DHF/DSS from DF patients, 313 MFs differentiated DHF/DSS from ND patients, and 309 MFs differentiated DF from ND patients (Fig 1).

MFs that statistically differentiated the dengue diagnosis groups were (when possible) given putative structural identification by interrogation of the Metlin and HMDB databases and the Omics discovery pipeline [47–49, 56, 57]. The metabolites are listed by MSI Level of identification in S1 and S2 Tables. In the Nicaraguan specimens, 13 identified metabolites (MSI Levels 1 and 2), 103 putatively identified metabolites (MSI Level 3), and 101 unidentified metabolites (MSI Level 4) differentiated the dengue diagnosis groups (S1 Table). In the Mexican specimens, 12 identified metabolites (MSI Levels 1 and 2), 120 putatively identified metabolites (MSI Level 3), and 182 unidentified metabolites (MSI Level 4) differentiated the diagnosis groups. Sixty-two of the differentiating metabolites were detected in both Nicaraguan and Mexican serum specimens (S1 and S2 Tables). Thirty-eight of the differentiating metabolites (denoted by **) exhibited a similar FC trend in the two groups; 24 metabolites exhibited an opposite FC trend (denoted by ***) in the two groups.

### Confirmation of metabolite identities

Thus far, the structural identities of 13 metabolites that statistically differentiate DHF/DSS, DF, and ND disease outcomes in at least one of the pairwise comparisons of the diagnosis groups have been confirmed using MS/MS (Table 4). These metabolites were grouped into biochemical classes including amino acids and lipids such as fatty acids and phospholipids, as well as vitamins.

The identities of six metabolites were confirmed (MSI level 1) by comparing the HILIC-LC-MS/MS spectrum of the candidate metabolite in the native serum with that of a commercial standard (Table 5). The spectra of MSI level 1 compounds that identified proline, *α*-linolenic acid (ALA), docosahexaenoic acid (DHA), lysophosphatidylcholine (lysoPC) (16:1), lysoPC (18:1) and arachidonic acid (AA), and the International Chemical Identifier (InChl) [58] for each of these metabolites are presented in S1–S6 Figs. The presence of the three vitamin D_3_ metabolites detected by HILIC-MS was validated (MSI level 1) by comparing the MRM spectrum of the candidate metabolite with that of a deuterated commercial standard using MRM LC-MS/MS [54, 55]. The spectra identifying endogenous vitamin D_3_, 25-hydroxyvitamin D_3_, 1,25-dihydroxyvitamin D_3_, and the InCHl identifiers are shown in S7–S9 Figs. The identities of four additional metabolites, myristoleic acid and three phosphatidylcholines (PCs) (34:1, 34:0, and 36:1) were confirmed (MSI level 2) by spectrum similarity with spectra in the NIST library [59].

The differentiating metabolites that were identified at MSI level 3 in Nicaraguan and Mexican samples are listed in S1 and S2 Tables, respectively. These metabolites (102 in Nicaraguan specimens and 121 in Mexican specimens) were identified *in silico* by interrogating online databases and libraries [47–49] and were assigned potential identities. These remain to be structurally confirmed. MFs that could not be identified *in silico* (101 in Nicaraguan specimens and 185 in Mexican specimens) but that were differentiated and quantified based on LC-MS spectrum data (MSI level 4) are also listed in S1 and S2 Tables.

### Identification of metabolites in Nicaraguan patients initially diagnosed as DF that predict progression to DHF/DSS

**Fig 4:**
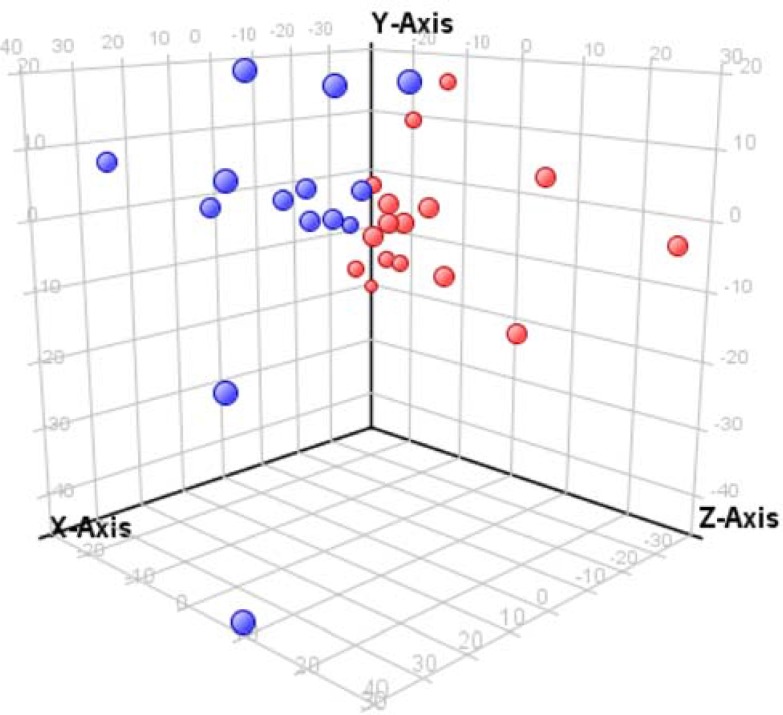
Principal component analysis of Nicaraguan serum samples from patients initially diagnosed with DF revealed clustering of patients who progressed to DHF/DSS and patients who did not progress to severe disease. Red spheres represent samples collected when a patient was initially classified as DF, who later progressed to DHF/DSS; blue spheres represent DF patients who did not progress to severe disease. The percentage of variation found is: X axis 14.93%, Y axis 9.74%, Z axis 8.25%.

Although the day of defervescence was unavailable for either Nicaraguan or Mexican patients, information regarding progression to DHF/DSS of patients initially diagnosed as DF was available for 31 Nicaraguan patients. These specimens were collected ≤ 4 days post onset of symptoms, presumably before the time of defervescence. Of these, 16 were collected from patients initially diagnosed as DF who later on progressed to DHF/DSS and 15 from patients who did not progress to DHF/DSS. The PCA plot revealed clustering of the patients who experienced unremarkable DF and those who progressed to severe dengue disease (Fig 4).

Statistical analysis of samples from these two patient groups yielded 65 metabolites that differentiated the eventual disease outcomes (S3 Table). Six metabolites were identified at MSI level 1 (Table 6), and all were previously identified (Table 5). The identified metabolites were proline, alpha-linolenic acid, arachidonic acid, docosahexaenoic acid, and two lysoPCs. The metabolites identified at MSI levels 3 and 4, which include 17 MFs at MSI level 3, predominantly lipids, and 44 MFs at MSI level 4, are listed in S3 Table. The potential metabolites (MSI level 3) include phosphatidylcholines, diacylglycerol, phosphatidic acid, phosphatidylserine, triglycerides, and diacylglycerophosphoglycerol.

**Table 6:** Metabolites in Nicaraguan serum samples of patients initially classified as DF that predicted progression to DHF/DSS*.

Mass	RT**	Compound ID	Chemical formula	DB identifier**	DF to DHF/DSS vs. DF P-value
MSI LEVEL 1**					
115.0635	16.45	Proline	C_5_H_9_NO_2_	HMDB00162	1.65E-02
278.2245	1.10	Alpha-linolenic acid	C_18_H_30_ O_2_	Metlin 192	2.49E-14
302.2243	1.13	Arachidonic acid	C_20_H_30_O_2_	Metlin 35293	1.27E-02
328.238	1.09	Docosahexaenoic acid	C_22_ H_32_ O_2_	Kegg C06429	1.48E-11
495.3326	13.74	LysoPC (16:0)	C_24_H_50_NO_7_P	Metlin 61692	2.91E-03
521.3492	13.54	LysoPC (18:1)	C_26_H_52_NO_7_P	Metlin 61695	5.17E-12

* DHF/DSS—dengue hemorrhagic fever/dengue shock syndrome; DF—dengue fever

**Abbreviations: RT—retention time; FC—fold change MetLin—Metabolite and Tandem Mass Spectrometry Database. HMDB—human metabolome database; KEGG- Kyoto Encyclopedia of Genes and Genomes MSI–Metabolomics Standard Initiative: level 1 –identity verified with a commercial standard. Bolded values indicate statistically significant differences in pairwise comparison of the two groups.

**Fig 5:**
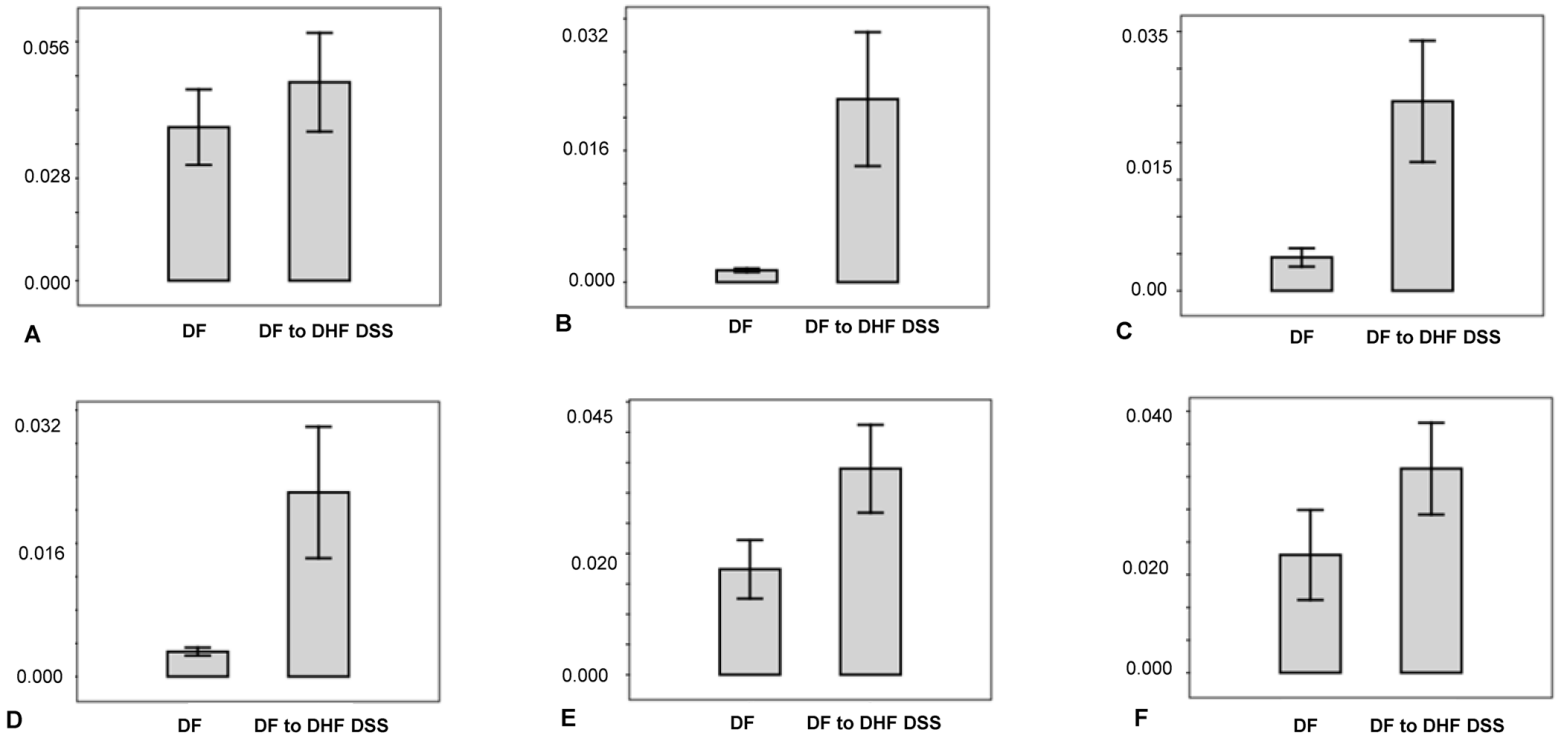
Relative abundance of differentiating metabolites in serum samples collected from patients initially diagnosed as DF who progressed to DHF/DSS and who did not progress to severe disease. A. proline (*p-value 4.60E-02). B. alpha-linolenic acid (*p-value 3.80E-04), C. arachidonic acid (*p-value 2.90E-04), D. docosahexaenoic acid (*p-value 4.30E-04), E. lysoPC (16:0) (*p-value 3.44E-05), F. (lysoPC (18:1) (*p-value 1.20E-02). DHF/DSS–dengue hemorrhagic fever/dengue shock syndrome; DF–dengue fever. (*) Statistically significant by two sample t test for unequal variances. Error bar–standard error.

Relative abundances of the six identified prognostic metabolites are presented in Fig 5. For this analysis, the data were further processed using Agilent Mass Hunter Quantitative Analysis software B.05.0, and the results were imported into PAST (Paleontological Statistics software package version 3.09). The abundances of the respective metabolite in DF and DF patients who later progressed to DHF/DSS patients were statistically compared using two sample t-test for unequal variances. Each of these metabolites was elevated in abundance in the DF patients that progressed to DHF/DSS compared to those who experienced DF disease.

The other metabolites listed in Table 6 and S3 Table are also candidate SMBs for progression to severe dengue disease and will be evaluated for their potential utility in predicting dengue disease outcomes.

## Discussion

Our studies confirm that DENV infection perturbs the human metabolome [32]. Statistical analyses indicated that many metabolites and MFs identified by HILIC-LC-MS had statistically significant differences in abundance in pairwise comparisons of the DHF/DSS, DF, and ND diagnosis groups (Table 5 and S1 and S2 Tables). Cui et al. [32] demonstrated perturbation of many of the same metabolites in DF patients during the time-course of primary DENV infection. Metabolites that were perturbed in DHF/DSS and DF patients in both Nicaraguan and Mexican patients included lysoPCs (14:0, 16:0) and long-chain polyunsaturated fatty acids such as DHA, AA, and ALA. To determine if differentiating metabolites identified by HILIC-LC-MS could be identified using a different LC-MS platform and to more thoroughly explore the metabolome, a subset of serum samples were analyzed in the Purdue Metabolite Profiling Facility (PMPF) using reverse phase (RP)-LC-MS [37]. In confirmation, 54% (117/288) of differentiating metabolites detected by HILIC-LC-MS were also detected using a T3 column (Waters, Milford, MA) in RP-LC-MS. All of the 13 differentiating metabolites whose identities were confirmed by LC-MS/MS (Table 5) were detected by RP-LC-MS and differentiated the dengue diagnosis groups.

These results provide proof of concept that differential perturbation of the serum metabolome is associated with different dengue infections and disease outcomes and that changes in relative concentrations of certain metabolites are associated with dengue diagnosis groups. Unfortunately, in this retrospective proof of concept study, a number of samples were obtained after the presumed time of defervescence and possible progression to severe disease (Tables 1 and 2). Thus, the differentiating metabolites identified in this retrospective study could represent metabolic perturbations reflecting the disease state instead of being predictive of progression to severe dengue disease. To address this issue, we compared the metabolic profiles of a subset of the Nicaraguan DF samples with those of DHF/DSS samples that were initially diagnosed as DF but then progressed to DHF/DSS (Fig 5); all of these samples were collected by day 4 of illness (presumably before the day of defervescence). Despite the small sample size, 65 metabolites differentiated the DF patients from those who progressed to DHF/DSS (S3 Table), including six of the structurally confirmed metabolites reported in Table 5 (proline, ALA, AA, DHA, and lysoPCs (16:0 and 18:1)) (Table 6). These current candidate SMBs are not specific for dengue disease but, when combined with DENV-positive laboratory test results (eg., NS1 antigen or viral RNA detection), may provide diagnosis and prognosis of DENV infection outcomes using acute-phase serum specimens.

Although these proof-of-concept prognostic metabolites are encouraging, they are based upon a small sample size and additional studies with increased numbers of patients will be needed to confirm the results. It must also be noted that these results are restricted to pediatric Nicaraguan patients. It cannot be assumed that the same metabolites will be predictive of progression to DHF/DSS in adult Nicaraguan patients or in patients from other geographic, genetic, and environmental backgrounds. Studies will be necessary to determine if these and/or alternate metabolites are predictive of progression to DHF/DSS in other patient populations.

It was surprising that PCA revealed little clustering of Mexican samples by dengue diagnosis group in contrast to the Nicaraguan samples (Fig 2A and 2B). Many factors have been demonstrated to condition dengue disease severity, including infecting DENV serotype and genotype and primary or secondary infections [24, 60, 61], which could have confounded the analyses. The Mexican patients were infected with either DENV-1 or DENV-2; only DENV-2 was detected in Nicaraguan patients. To explore reasons for the lack of segregation of dengue diagnosis groups in PCA, we analyzed Mexican samples stratified by infecting virus serotype (DENV-1 versus DENV-2). PCA and statistical analyses revealed significant differences in the perturbation of the serum metabolome of Mexican patients attributable to the different serotypes (Fig 3A, S4 Table). In this regard, the different numbers of DENV2 infections in Mexico and Nicaragua (22 and 59, respectively, could have confounded the results (Tables 1 and 2). We also explored the potential role of primary versus secondary infections in perturbation of the serum metabolome of dengue patients. Immune status was only available for the Nicaraguan samples, which were stratified by primary versus secondary infection and analyzed. PCA plots revealed clustering of patients by primary versus secondary infection, and analyses revealed multiple metabolites that differentiated infection by immune status (Fig 3B and S5 Table). This analysis of the Nicaraguan samples clearly demonstrates differential perturbations according to immune status, which are likely occurring in Mexican patients as well. Unfortunately immune status was only known for a few of the Mexican patients. Clearly the differences demonstrated for the Nicaraguan samples could have confounded the analyses of the Mexican samples. In addition the Mexican samples differed from the Nicaraguan samples in age distribution (the effect of age on dengue disease severity and metabolome perturbations is addressed below). All of these and other factors could have contributed to the lack of clustering in the Mexican patients by diagnosis group, and additional metabolomics studies will be necessary to identify the actual mechanisms involved.

Thus, although the available sample sizes were relatively small in this proof-of-concept study, PCA plots revealed clustering of patients by both infecting virus serotypes (Fig 3A) and by primary versus secondary infection, (Fig 3B) and differentiating metabolites were identified for each comparison (S4 and S5 Tables). Interestingly, none of the differentiating metabolites in these two analyses overlapped, suggesting the involvement of different metabolic pathways or mechanisms. It is also interesting that these metabolites differ from those reported in Table 5 that differentiated the dengue disease diagnosis groups (DHF/DSS versus DF, DHF/DSS versus ND and DF versus ND).

Differentiating metabolites identified in this study provide insights into fundamental metabolic mechanisms and pathways that condition DENV infection, replication, and pathogenesis in humans, and several are potentially biologically and physiologically relevant in terms of severe disease outcomes (DHF/DSS). Some are associated with lipid metabolism and regulation of inflammatory processes controlled by signaling fatty acids and phospholipids. Others are associated with immune regulation, endothelial function, and vascular barrier function, which is provocative in the context of the central role of vascular leakage in the pathogenesis of DENV infection and the possible progression to shock in DSS [3, 4, 7, 30].

DENV replication is dependent on host cell lipid biosynthesis and metabolism. Viral replication complexes are enclosed in endoplasmic reticulum-derived double-membrane vesicles that organize and localize the complexes to facilitate the exchange of components with the cytosol for genome replication and virus assembly [37, 62, 63]. Long-chain polyunsaturated fatty acids such as DHA (C22:6) and ALA (C18:3) were increased in abundance in DHF/DSS versus DF and DHF/DSS versus ND groups in Nicaraguan serum samples and in early DF that progressed to DHF/DSS (Tables 5 and 6). Long-chain omega-3 polyunsaturated fatty acids such as DHA and its precursor ALA are potent anti-inflammatory agents and have been previously reported to be elevated during DENV infection [32]. DHA has been shown to decrease the production of inflammatory eicosanoids, cytokines, and reactive oxygen species [64, 65]. This molecule can act both directly by inhibiting AA metabolism and indirectly by altering the expression of inflammatory gene products [66, 67]. DHA also is a precursor of a family of anti-inflammatory mediators called D-series resolvins [66, 67]. The increases in DHA levels we observed in dengue patient serum might represent the host attempt to mitigate immunopathology of dengue disease.

AA and its metabolites have been shown to be elevated in plasma at different stages of infection in dengue patients [32, 68]. This was confirmed in our results; we found AA levels elevated in DHF/DSS patients compared to DF patients in both Mexican and Nicaraguan populations. AA is mobilized from phospholipids in cell membranes and is metabolized by cyclooxygenases and lipoxygenases to pro-inflammatory eicosanoids such as prostaglandins, thromboxanes and leukotrienes [69, 70]. We detected significant changes in abundances of several of these AA metabolites when comparing the dengue disease groups (Tables 5 and 6).

A number of phospholipid metabolites differentiated dengue diagnosis groups in patients from both Nicaragua and Mexico (Table 5 and S1 and S2 Tables). The increases we observed in phospholipid biosynthesis make biological sense given that host cell phospholipid metabolism is known to be influenced by DENV replication in both mosquito and human cells through DENV NS3 protein-mediated redistribution and activation of fatty acid synthase [37, 61]. The prevalent phospholipids found to be increased primarily contain C16 and C18 unsaturated acyl chains. Palmitic acid (C16) and oleic acid (C18) have been found to be increased in DENV-infected mosquito cells and to facilitate production of lysoPCs. Phospholipids are precursors of lipid mediators, such as platelet activating factors (PAFs) and eicosanoids, which are involved in inflammatory responses [70, 71].

LysoPCs (18:1, 16:0) were elevated in acute-phase serum specimens of DHF/DSS patients (Table 5). These single fatty acid chain lipids are involved in alteration of membrane structures and can mediate acute inflammation and regulate pathophysiological events throughout the vasculature and at local tissue sites [72–75]. Interestingly, lysoPCs may alter homeostasis of vascular endothelium, causing endothelial cell instability, barrier dysfunction, and vascular leakage, a major component of the pathophysiology of DSS [18, 76–78]. Previous reports demonstrated perturbation of lysoPC concentrations in DENV-infected human serum [32, 37]. Up-regulation of the phosphatidylcholine biosynthesis pathway in acute DENV infections (days 1–3) was identified as one of the predictors for progression to DHF [19]. Other metabolites from different biochemical classes differentiated dengue disease outcomes. For example, we observed lower levels of 1,25-dihydroxyvitamin D3 (1,25-vitD_3_) in DHF/DSS versus DF and ND in Nicaraguan patients (Table 5). Reduced levels of 1,25-vitD_3_, with its roles in immunoregulation and vascular barrier function, could be involved in the immunopathophysiology associated with DHF/DSS [79]. A decrease in serum 1,25-vitD_3_ levels is associated with increased mortality in sepsis patients [80, 81]. The active form of vitamin D_3_ (1,25-vitD_3_) can be synthesized in vascular endothelium following stimulation of vitamin D3 1α-hydroxylase activity by inflammatory cytokines. Interactions of this metabolite with endothelial cells and the reduction of 1,25-vitD_3_ observed in immune-mediated diseases by others [79, 82] prompts speculation about the potential role of decreased concentrations of this metabolite in patients progressing to DHF/DSS. Interestingly, polymorphisms in the vitamin D receptor gene are linked with severe dengue disease outcomes [14].

Several amino acids or peptides were also found to differentiate disease outcomes. For example, proline, which can act as a modulator of the intracellular redox environment, differed in DHF/DSS and DF patients who were initially diagnosed as DF (Fig 5). Perturbations of proline in endothelial cells could affect endothelium function [83–85].

Clearly, metabolomics provides new opportunities and a powerful approach to investigate potential viral, host, pathogenic, and immunologic determinants of DENV infection and pathogenesis.

Identification of metabolites that differentiate dengue disease outcomes in patients from different geographic areas, environmental conditions, genetic backgrounds, sexes, and ages [34, 35] is an important first step in selecting SMBs for dengue diagnosis and prognosis. We have already identified a large, overlapping set of metabolites that differentiated dengue outcomes in genetically and geographically distinct populations. However, the associations were not always concordant. In some instances, a metabolite differentiated the disease outcomes in one study population but not in the other. For example, lysoPCs (16:0 and 18:1) statistically differentiated DHF/DSS and DF diagnosis groups in Nicaraguan patients but not in Mexican patients (Table 5). In other instances, a candidate SMB was either increased or decreased in abundance in serum from patients from one country and the opposite trend occurred in patients from the other country. Sixty-two of the differentiating metabolites were detected in serum specimens from the Nicaraguan and Mexican patients (see Table 5 and S1 and S2 Tables). Thirty-eight of these differentiating metabolites (denoted by **) had similar FC trends in Nicaraguan and Mexican patients, but 24 (denoted by ***) had opposite FC trends. For example, ALA and DHA exhibited positive fold-changes in differentiating DHF/DSS from DF and ND outcomes in Nicaraguan patient sera but negative trends in Mexican patients.

A number of factors could have contributed to the dissimilar change trends in the two populations. For example in this exploratory metabolomics study, no Mexican patient was officially diagnosed as DSS (although some were hospitalized and diagnosed with DHF), while 15% of the DHF/DSS patients in Nicaragua were classified as DSS. The lack of DSS cases in Mexico is a limitation of our study that may have confounded the identification of SMBs that differentiate DSS from non-DSS disease outcomes. In addition, there were major age differences in the two study populations. The age of the patient can condition dengue disease severity [86, 87]. Severity of symptoms (which can be subjective) that influence clinical diagnosis and disease classification in the two populations could also be strongly influenced by the age of the participants. Ninety percent of the Mexican patients were >15 years of age and may have been less likely to progress to DSS even though they were diagnosed as DHF patients. All of the Nicaraguan patients were children <15 years of age. Human metabolic profiles are age-dependent [88], and DENV pathophysiology and clinical symptomology (e.g., DHF) can differ in different age groups [86, 87, 89] and by sex [60]. DSS is negatively correlated with age [13]. Liver inflammation (an important target organ in DENV infection) is more prevalent in children than in adults [90, 91].

In this regard, we conducted a very preliminary analysis to examine the potential role of age on the DENV-infected serum metabolome. The Mexican patients with DF or DHF/DSS ranged from 1 to 62 years of age. These DENV-infected samples were stratified by age; pediatric patients <15 years of age (N = 11) and adult patients >15 years of age (N = 57), and the serum metabolites were characterized by PCA (S10 Fig). Because of the limited number of pediatric patients, in this preliminary analysis we used a filter of 25% metabolite presence in samples of one of the diagnosis groups instead of our standard filter of 50% metabolite presence. Despite the small number of pediatric patients, clustering of patients by age was evident (S10 Fig). Clearly, age differences could have contributed to the metabolomic differences between the two groups. The effect of age on the serum metabolome during DENV-infections will be a fruitful area of research. Identification of metabolites that differentiate age groups could provide important insights into differences in the pathophysiology of DENV infections in pediatric and adult patients [86, 87].

Other factors could also contribute to the dissimilar change trends in metabolite abundance in the two populations. Dietary differences between Nicaraguan and Mexican patients could confound results with metabolites such as ALA, which is obtained principally from dietary plant sources, and DHA, which is a metabolite of ALA. All of these factors could account for some of the metabolite differences seen in the two study populations. Further studies will be necessary to determine if metabolites such as lysoPCs and DHA are candidate SMBs for progression to DSS in older patients and in patients from other geographic areas.

We are currently conducting a prospective clinical study in Managua, Nicaragua, to determine the diagnostic and prognostic potential of existing and yet to be identified candidate SMBs. MRM analysis [92] will be conducted for accurate quantification of abundance of metabolites in different disease states as part of the evaluation of the potential diagnostic utility of candidate SMBs. Super learner analysis [93] is being used to identify the most parsimonious SMB “biosignature” in acute phase serum specimens that, when combined with other laboratory and clinical information, such as NS1 antigen detection and viral RNA detection by RT-PCR, will provide the most efficient algorithms for dengue diagnosis and prognosis. This would be of enormous value for appropriate patient triaging, management and clinical care. Prospective clinical studies will allow us to increase the number of early acute-phase patients and to identify additional metabolites that are predictive of progression to severe dengue disease. Additional clinical studies potentially will also allow us to increase the number of patients who progress to DSS and to identify metabolites that differentiate DHF and DSS disease outcomes [28]. The studies will also provide insights into metabolic pathways and pathogenic mechanisms that condition DHF and DSS outcomes. Such information could potentially be exploited in the development of new therapeutics for treatment of dengue patients in danger of progressing to DSS [28]. The 3- to 4-day window from dengue disease onset to defervescence provides a unique opportunity for therapeutic intervention [3–5, 7, 30]. Clearly, metabolomics provides new opportunities for diagnosis and prognosis of DENV infections.

## Supporting Information

S1 Fig Docosahexaenoic acid (DHA) spectra and chemical structure. A. MS/MS fragmentation pattern of the commercial standard. B. MS/MS fragmentation pattern of a representative serum sample. InChl key: MBMBGCFOFBJSGT-UHFFFAOYSA-N.
(TIFF)

S2 Fig α-linolenic acid (ALA) spectra and chemical structure. A. MS/MS fragmentation pattern of the commercial standard. B. MS/MS fragmentation pattern of a representative serum sample. InChl key: DTOSIQBPPRVQHS-UHFFFAOYSA-N.
(TIFF)

S3 Fig Arachidonic acid (AA) spectra and chemical structure. A. MS/MS fragmentation pattern of the commercial standard. B. MS/MS fragmentation pattern of a representative serum sample. InChI Key: GIOQWSLKUVKKAO-QNEBEIHSSA-N.
(TIFF)

S4 Fig Lysophosphatidylcholine (16:0) spectra and chemical structure. A. MS/MS fragmentation pattern of the commercial standard. B. MS/MS fragmentation pattern of a representative serum sample. InChI Key: ASWBNKHCZGQVJV-HSZRJFAPSA-N.
(TIFF)

S5 Fig Lysophosphatidylcholine (18:1) spectra and chemical structure. A. MS/MS fragmentation pattern of the commercial standard. B. MS/MS fragmentation pattern of a representative serum sample. InChI Key: YAMUFBLWGFFICM-PTGWMXDISA-N.
(TIFF)

S6 Fig Proline spectra and chemical structure. A. MS/MS fragmentation pattern of the commercial standard. B. MS/MS fragmentation pattern of a representative serum sample. International chemical identifier (InChl) key: ONIBWKKTOPOVIA-BYPYZUCNSA-N.
(TIFF)

S7 Fig MRM detection and conformation of endogenous 1,25-dihydroxyvitamin D3. Retention time is 8.3 minutes. A. Internal standard [2H]6–1,25-dihydroxy vitamin D3, B. 1,25-dihydroxy vitamin D3. InChl key GMRQFYUYWCNGIN-NKMMMXOESA-N.
(TIFF)

S8 Fig MRM detection and conformation of endogenous 25-hydroxyvitamin D3. Retention time is 9.9 minutes. A. Internal standard [2H]3-25-hydroxyvitamin D3, B. 25-hydroxyvitamin D3. InChI Key: JWUBBDSIWDLEOM-DTOXIADCSA-N.
(TIFF)

S9 Fig MRM detection and conformation of endogenous vitamin D3. Retention time is 13.8 minutes. A. Internal standard [2H]6-vitamin D3. B. vitamin D3. InChI Key: QYSXJUFSXHHAJI-YRZJJWOYSA-N.
(TIFF)

S10 Fig Principal component analysis reveals clustering of dengue virus infected Mexican patients by age. Blue spheres represent Mexican pediatric patients <15 years of age; red spheres represent adult patients >15 years of age. The percentage of variation found is: X axis 12.98%, Y axis is 7.87% and Z axis is 6.32%.
(TIF)

S1 Table Nicaraguan acute-phase serum metabolites that differentiate dengue outcomes in at least one of the pairwise comparisons of the DHF/DSS, DF, and ND* diagnosis groups. MSI Level 1: Metabolites identified by HILIC- MS/MS*or MRM-LC-MS/MS spectra matches with spectra of chemical reference standards acquired on the same analytical platform. MSI Level 2: Metabolites identified by HILIC-MS* and spectrum similarity with public/commercial spectrum libraries. MSI Level 3: Metabolites putatively identified based on physicochemical characteristics of a chemical class of compounds or by spectrum similarity to known compounds. MSI Level 4: Unidentified or unclassified metabolites that can be differentiated or quantified based on spectrum data. Bolded values statistically differentiated the pairwise comparison of the two diagnosis groups.
(DOCX)

S2 Table Mexican serum metabolites that differentiate dengue outcomes in at least one of the pairwise comparisons of the DHF/DSS, DF, and ND* diagnosis groups. MSI Level 1: Metabolites identified by HILIC- MS/MS*or MRM-LC-MS/MS* spectra matched with spectra of chemical reference standards acquired on the same analytical platform. MSI Level 2: Metabolites identified by HILIC-MS* and spectrum similarity with public/commercial spectrum libraries. MSI Level 3: Metabolites putatively characterized based on physicochemical characteristics of a chemical class of compounds or by spectrum similarity to known compounds. MSI Level 4: Unidentified or unclassified MFs that can be differentiated or quantified based on spectrum data. Bolded values statistically differentiated the pairwise comparison of the two diagnosis groups.
(DOCX)

S3 Table Metabolites in Nicaraguan sera collected ≤ 4 days after onset of symptoms that predicted progression of DF patients to DHF/DSS. MSI Level 1: Metabolites identified by HILIC- MS/MS*or MRM-LC-MS/MS spectra matches with spectra of chemical reference standards acquired on the same analytical platform. MSI Level 3: Metabolites putatively identified based on physicochemical characteristics of a chemical class of compounds or by spectrum similarity to known compounds. MSI Level 4: Unidentified or unclassified metabolites that can be differentiated or quantified based in spectrum data. Bolded values indicate statistically significant differences in pairwise comparison of the two diagnosis groups.
(DOCX)

S4 Table Mexican acute phase serum metabolites that differentiate DENV-1 and DENV-2 infections. MSI Level 3: Metabolites putatively identified based on physicochemical characteristics of a chemical class of compounds or by spectrum similarity to known compounds. MSI Level 4: Unidentified or unclassified metabolites that can be differentiated or quantified based in spectrum data. Bolded values show those that statistically differentiated pairwise comparisons of the two DENV serotypes. None of the following metabolites have been structurally characterized by LC-MS/MS to be classified at MSI Levels 1 or 2.
(DOCX)

S5 Table Nicaraguan acute-phase serum metabolites that differentiate dengue primary-type vs. secondary-type immune response in pairwise comparisons. MSI Level 3: Metabolites putatively identified based on physicochemical characteristics of a chemical class of compounds or by spectrum similarity to known compounds. MSI Level 4: Unidentified or unclassified metabolites that can be differentiated or quantified based in spectrum data. Bolded values indicate statistically significant differences in the pairwise comparison of the two immune response groups. The identities of the metabolites will be subjected to further LCMS-MS analysis to be classified at MSI levels 1 and 2.
(DOCX)
