# Supplementary figures and images for: Metabolomics-Based Discovery of Small Molecule Biomarkers in Serum Associated with Dengue Virus Infections and Disease Outcomes
Source: PLoS Negl Trop Dis. 2016 Feb 25;10(2):e0004449. doi: 10.1371/journal.pntd.0004449 (PMC4768770; doi:10.1371/journal.pntd.0004449)

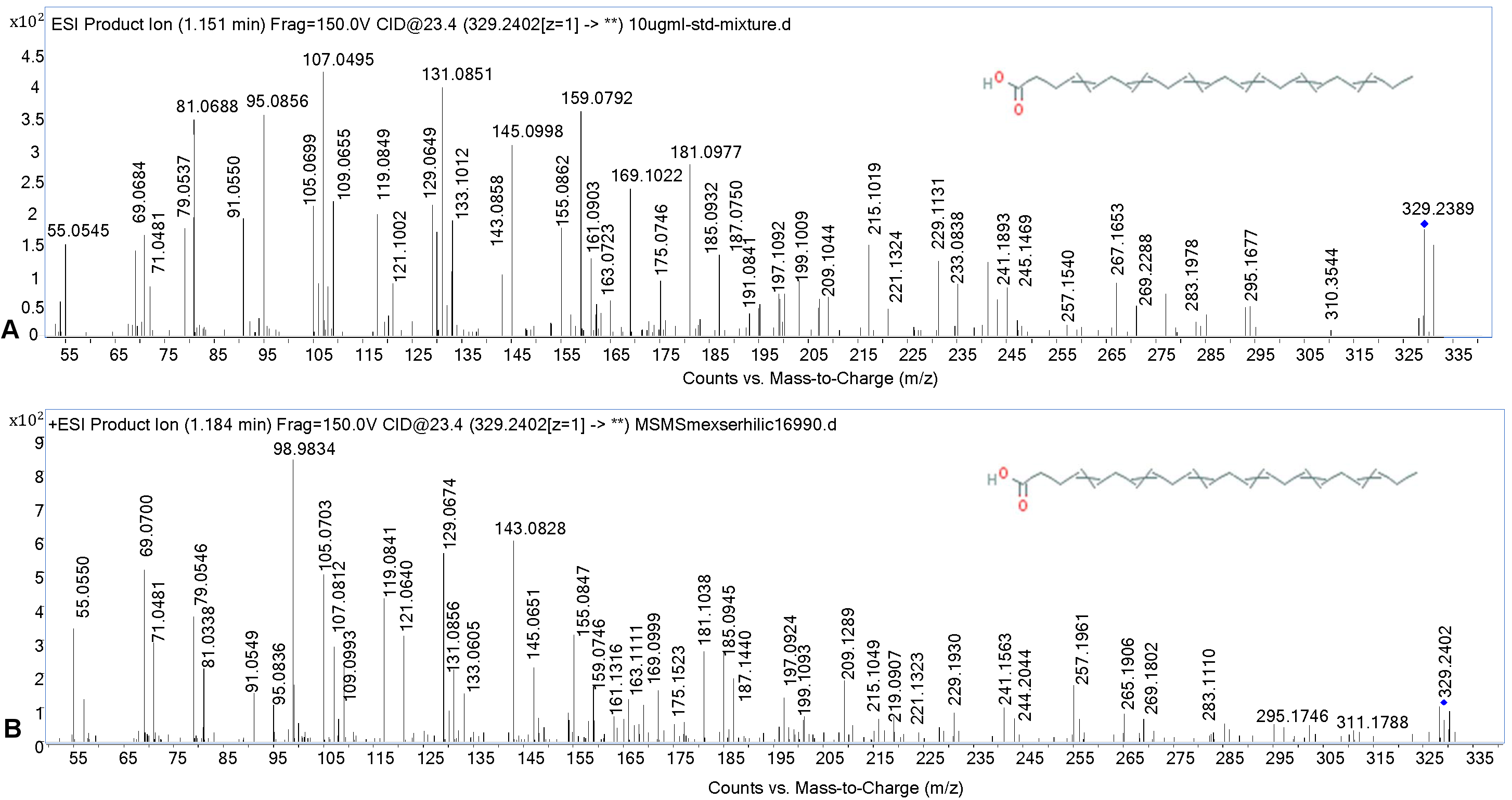

Supplement: S1 Fig — A. MS/MS fragmentation pattern of the commercial standard. B. MS/MS fragmentation pattern of a representative serum sample. InChl key: MBMBGCFOFBJSGT-UHFFFAOYSA-N. (TIFF) [file pntd.0004449.s001.tiff]

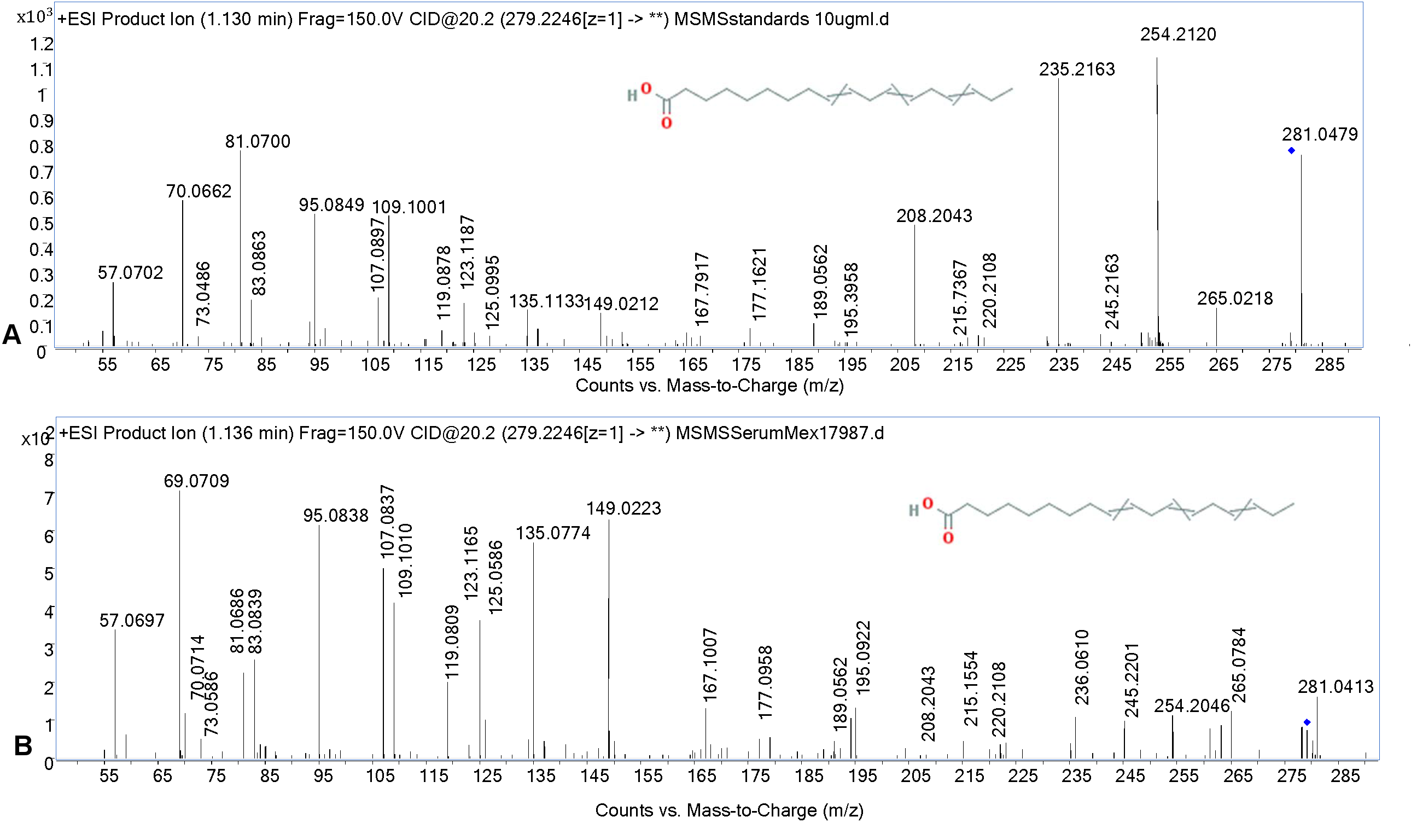

Supplement: S2 Fig — A. MS/MS fragmentation pattern of the commercial standard. B. MS/MS fragmentation pattern of a representative serum sample. InChl key: DTOSIQBPPRVQHS-UHFFFAOYSA-N. (TIFF) [file pntd.0004449.s002.tiff]

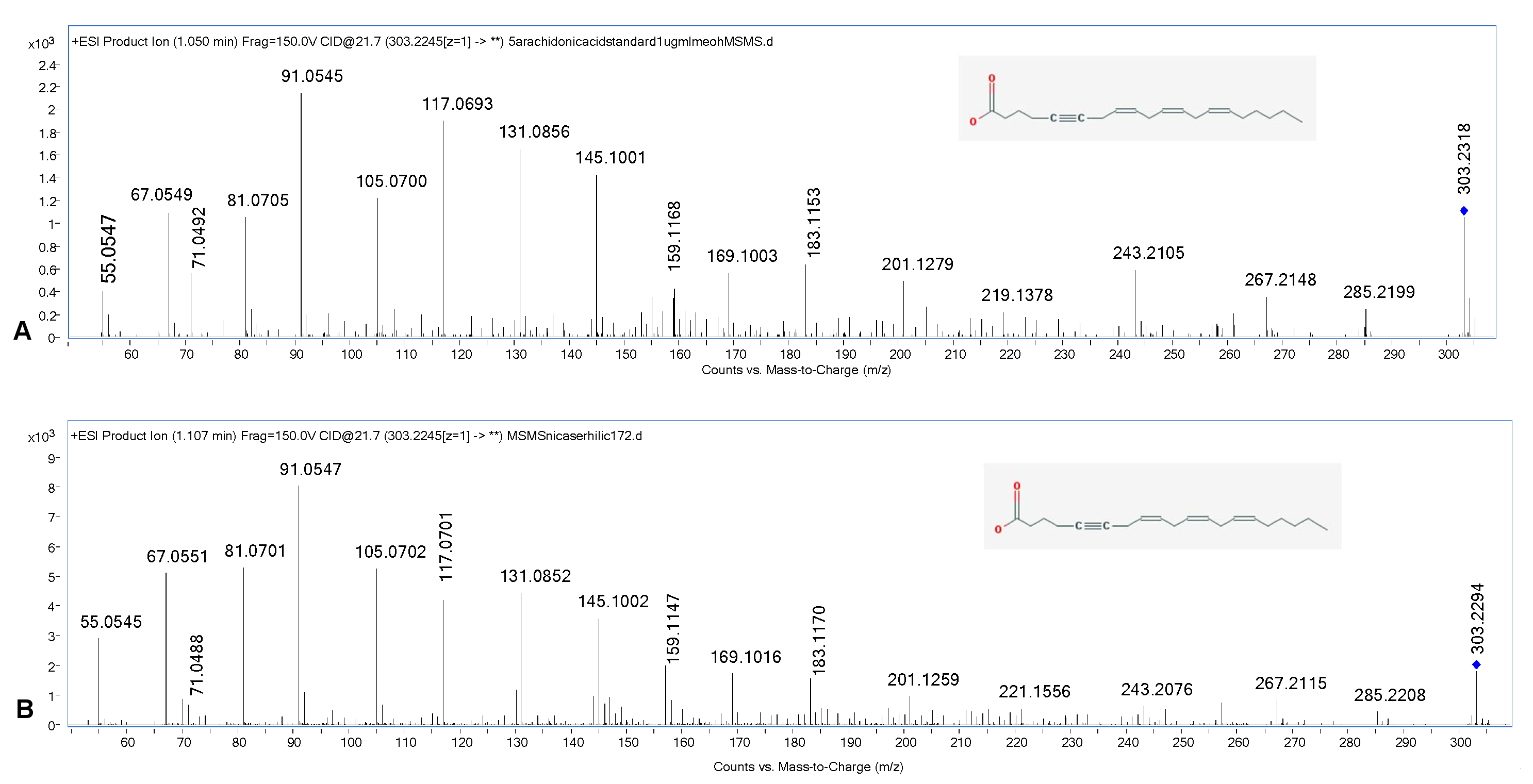

Supplement: S3 Fig — A. MS/MS fragmentation pattern of the commercial standard. B. MS/MS fragmentation pattern of a representative serum sample. InChI Key: GIOQWSLKUVKKAO-QNEBEIHSSA-N. (TIFF) [file pntd.0004449.s003.tiff]

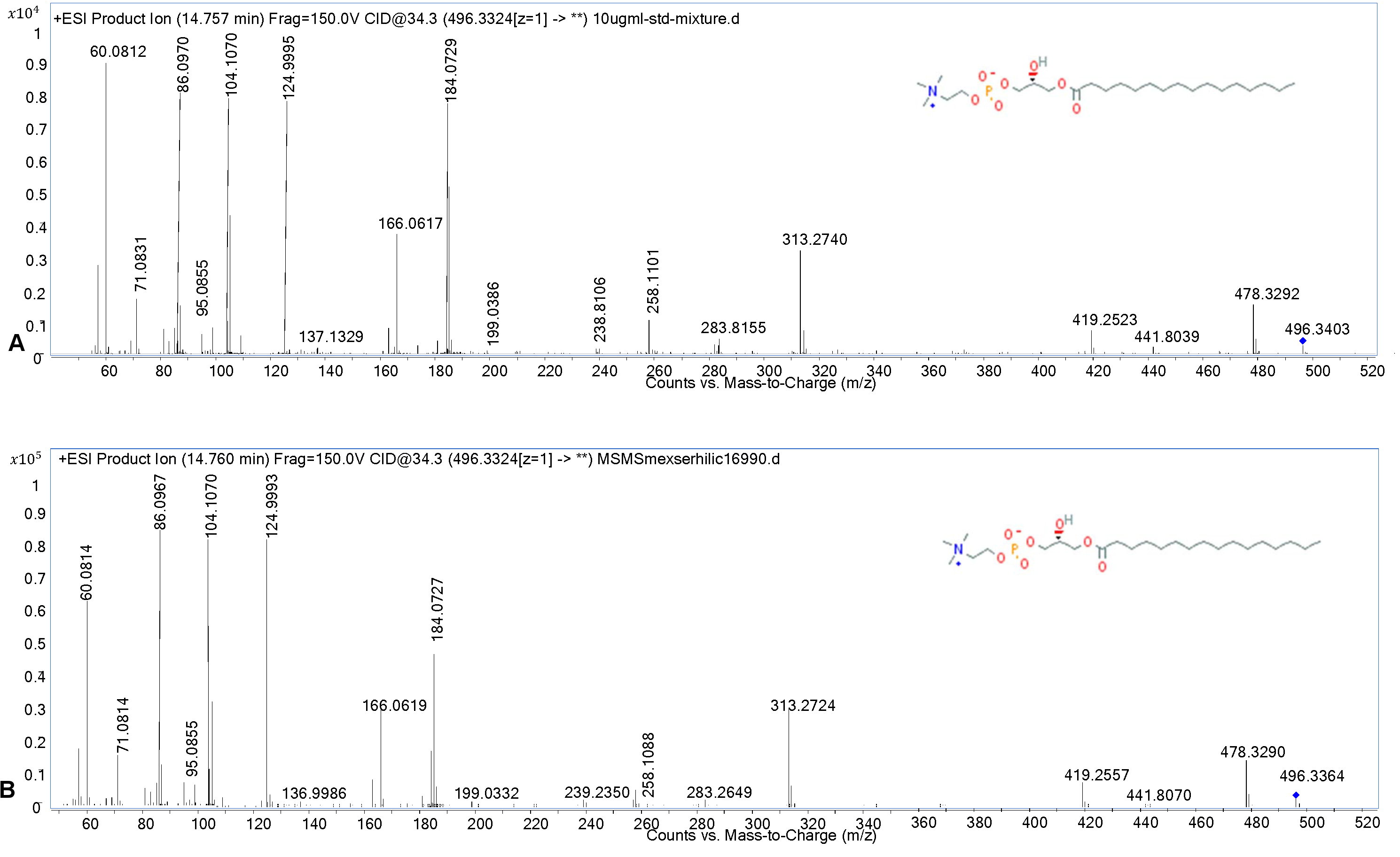

Supplement: S4 Fig — A. MS/MS fragmentation pattern of the commercial standard. B. MS/MS fragmentation pattern of a representative serum sample. InChI Key: ASWBNKHCZGQVJV-HSZRJFAPSA-N. (TIFF) [file pntd.0004449.s004.tiff]

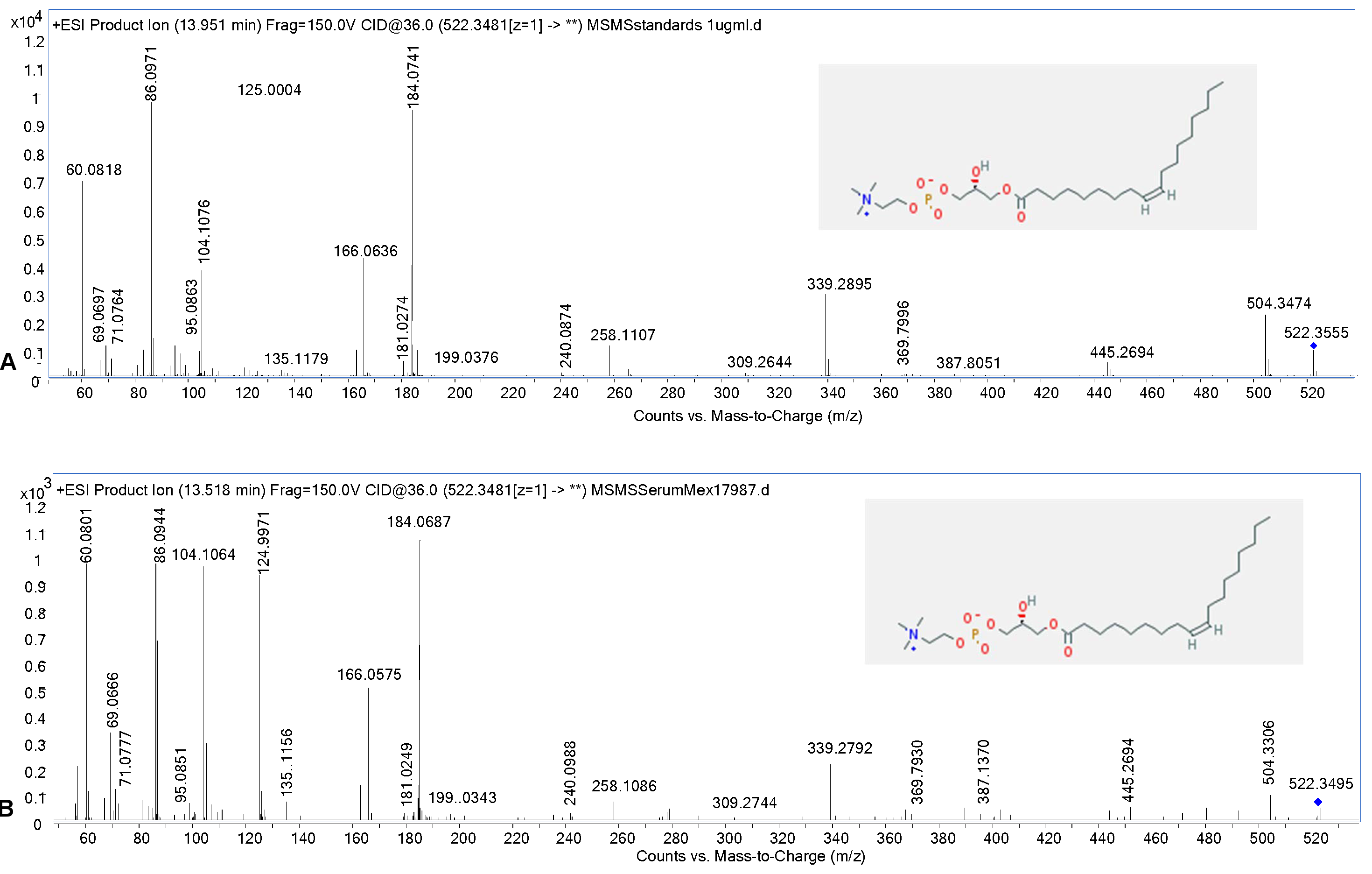

Supplement: S5 Fig — A. MS/MS fragmentation pattern of the commercial standard. B. MS/MS fragmentation pattern of a representative serum sample. InChI Key: YAMUFBLWGFFICM-PTGWMXDISA-N. (TIFF) [file pntd.0004449.s005.tiff]

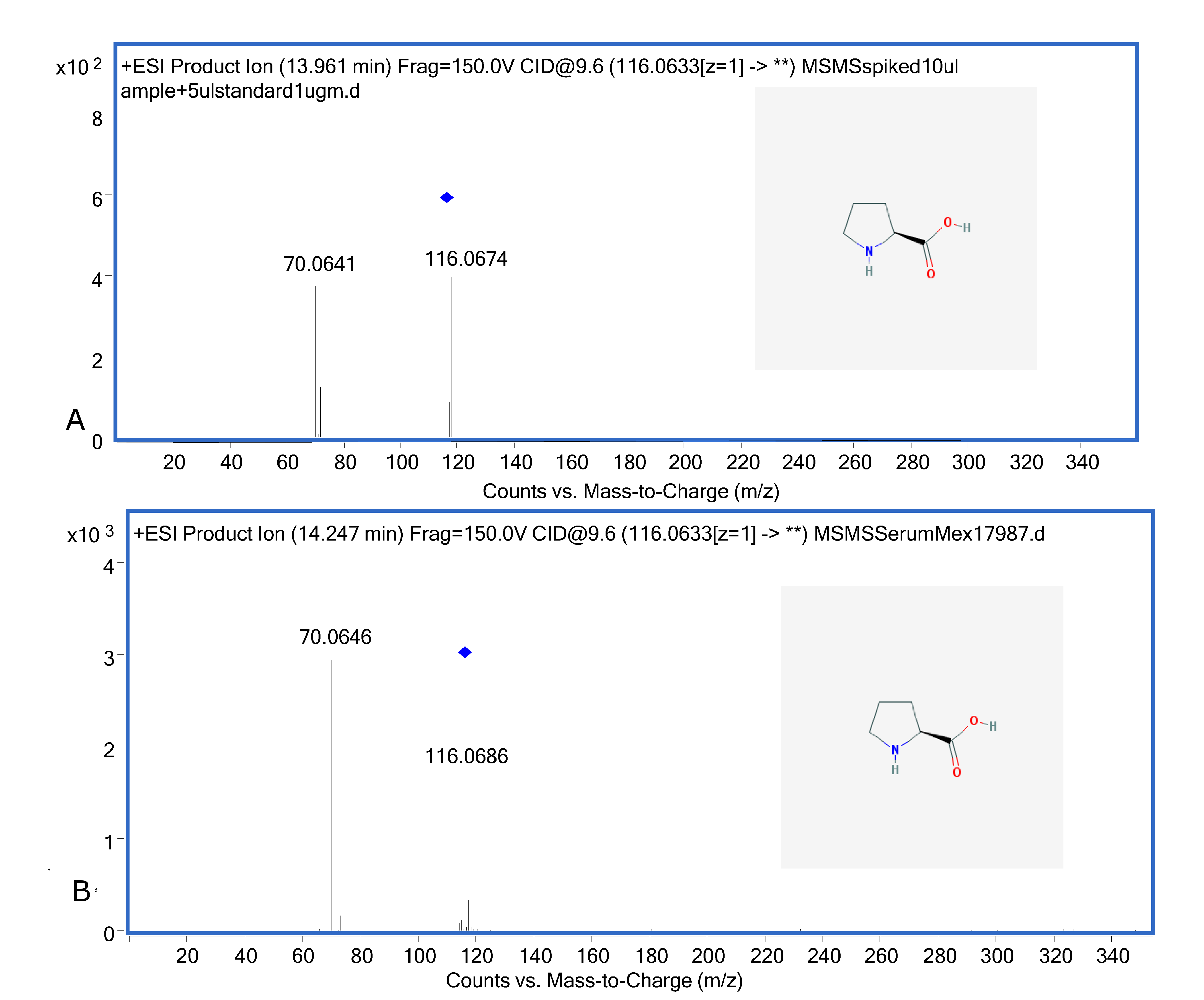

Supplement: S6 Fig — A. MS/MS fragmentation pattern of the commercial standard. B. MS/MS fragmentation pattern of a representative serum sample. International chemical identifier (InChl) key: ONIBWKKTOPOVIA-BYPYZUCNSA-N. (TIFF) [file pntd.0004449.s006.tiff]

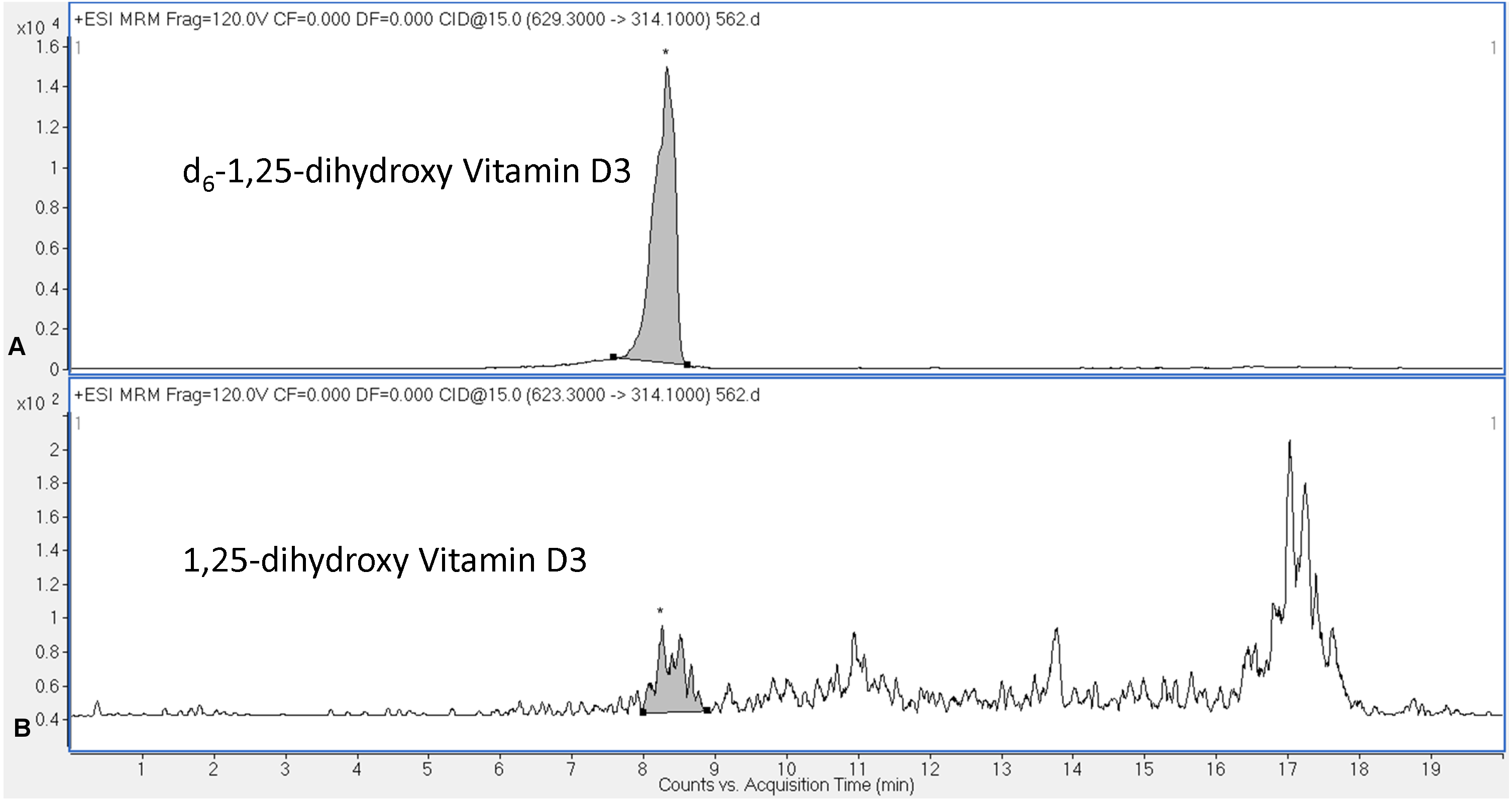

Supplement: S7 Fig — Retention time is 8.3 minutes. A. Internal standard [2H]6–1,25-dihydroxy vitamin D3, B. 1,25-dihydroxy vitamin D3. InChl key GMRQFYUYWCNGIN-NKMMMXOESA-N. (TIFF) [file pntd.0004449.s007.tiff]

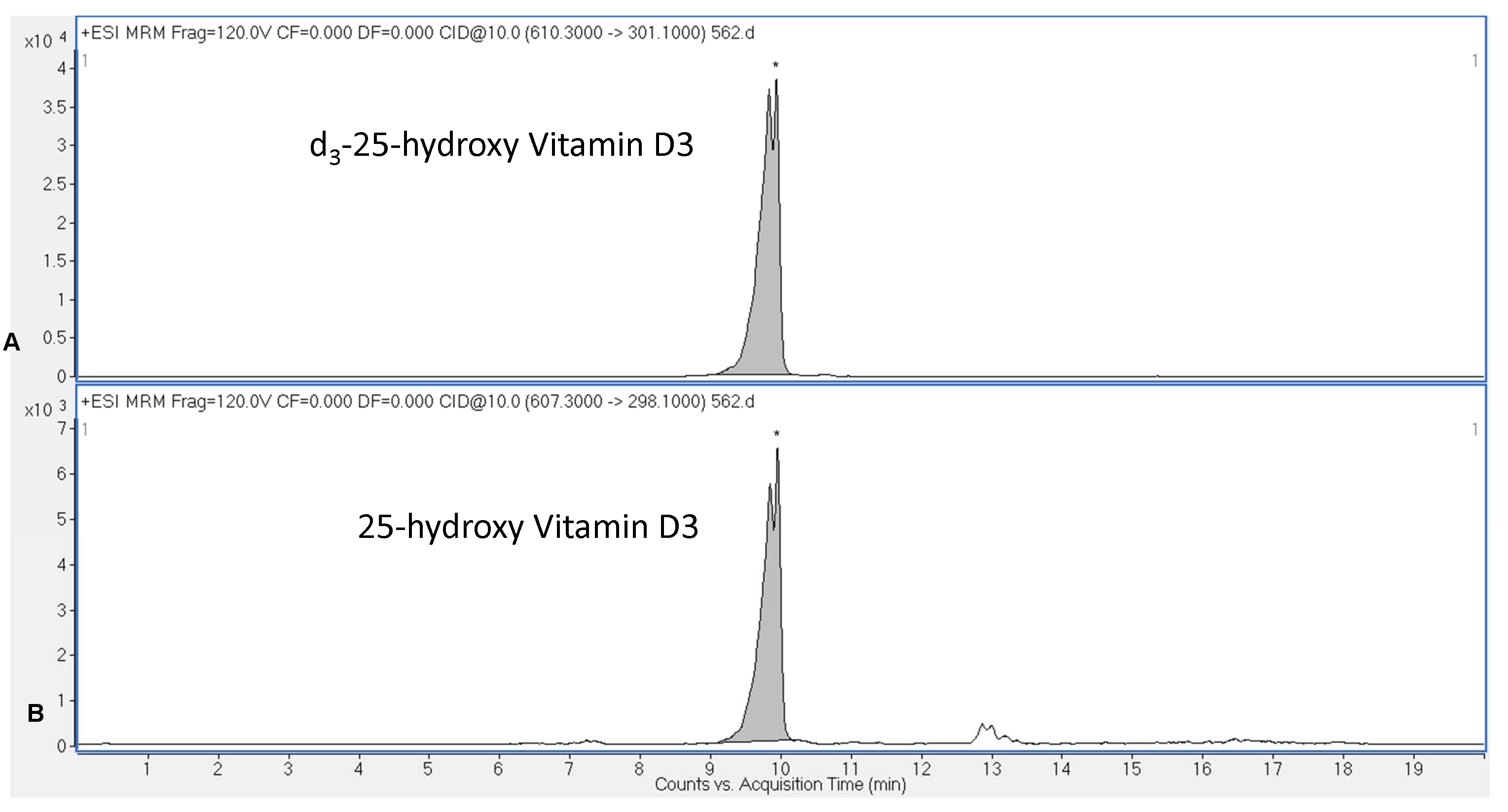

Supplement: S8 Fig — Retention time is 9.9 minutes. A. Internal standard [2H]3-25-hydroxyvitamin D3, B. 25-hydroxyvitamin D3. InChI Key: JWUBBDSIWDLEOM-DTOXIADCSA-N. (TIFF) [file pntd.0004449.s008.tiff]

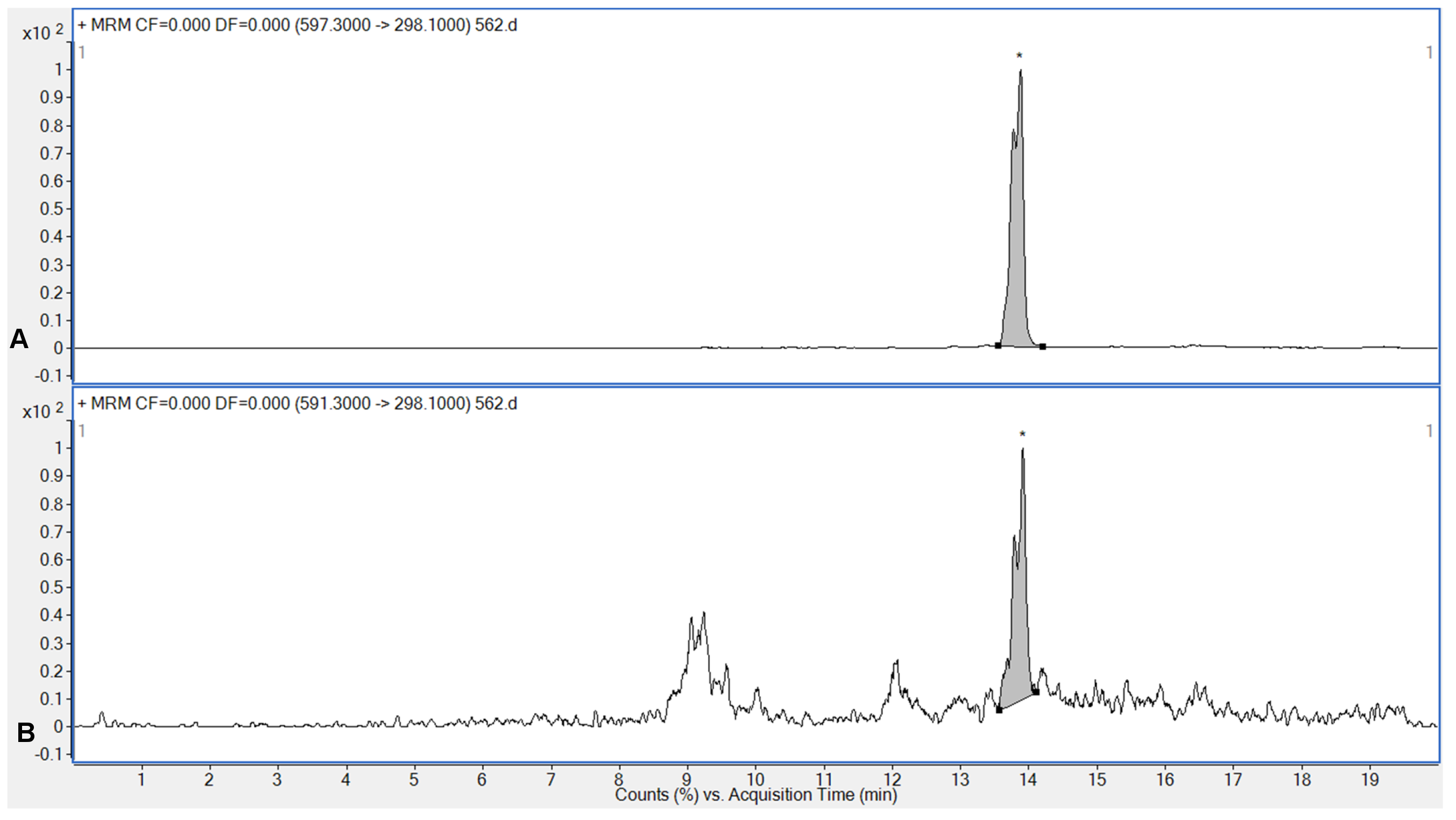

Supplement: S9 Fig — Retention time is 13.8 minutes. A. Internal standard [2H]6-vitamin D3. B. vitamin D3. InChI Key: QYSXJUFSXHHAJI-YRZJJWOYSA-N. (TIFF) [file pntd.0004449.s009.tiff]

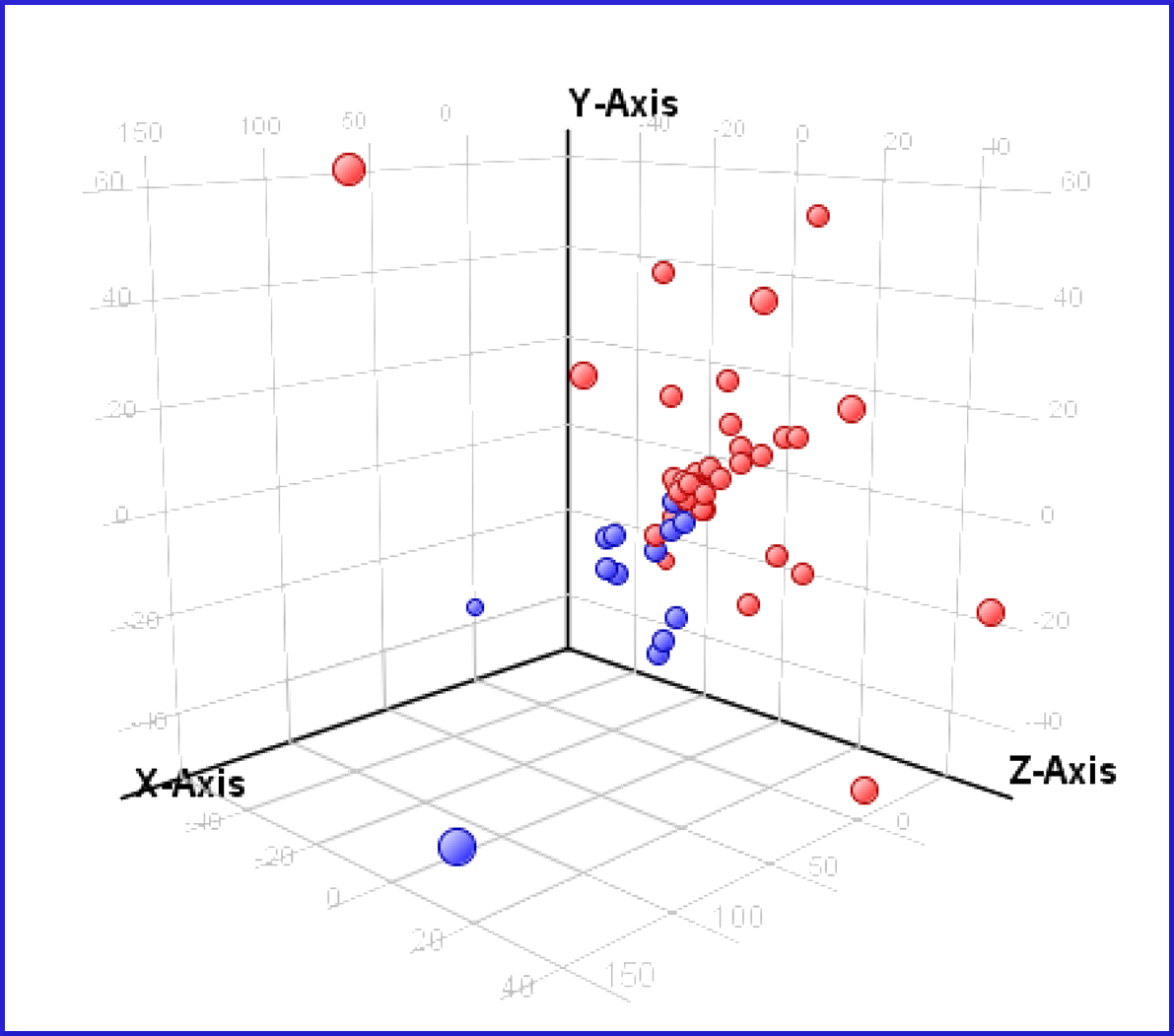

Supplement: S10 Fig — Blue spheres represent Mexican pediatric patients <15 years of age; red spheres represent adult patients >15 years of age. The percentage of variation found is: X axis 12.98%, Y axis is 7.87% and Z axis is 6.32%. (TIF) [file pntd.0004449.s010.tif]
