# Supplementary material for: Metabolomics-Based Discovery of Small Molecule Biomarkers in Serum Associated with Dengue Virus Infections and Disease Outcomes
Source: PLoS Negl Trop Dis. 2016 Feb 25;10(2):e0004449. doi: 10.1371/journal.pntd.0004449 (PMC4768770; doi:10.1371/journal.pntd.0004449)
Supplement: S1 Table — MSI Level 1: Metabolites identified by HILIC- MS/MS*or MRM-LC-MS/MS spectra matches with spectra of chemical reference standards acquired on the same analytical platform. MSI Level 2: Metabolites identified by HILIC-MS* and spectrum similarity with public/commercial spectrum libraries. MSI Level 3: Metabolites putatively identified based on physicochemical characteristics of a chemical class of compounds or by spectrum similarity to known compounds. MSI Level 4: Unidentified or unclassified metabolites that can be differentiated or quantified based on spectrum data. Bolded values statistically differentiated the pairwise comparison of the two diagnosis groups. (DOCX) [file pntd.0004449.s011.docx]

**S1 Table. Nicaraguan acute-phase serum metabolites that differentiate dengue outcomes in at least one of the pairwise comparisons of the DHF/DSS, DF, and ND* diagnosis groups.** MSI Level 1: Metabolites identified by HILIC- MS/MS*or MRM-LC-MS/MS spectra matches with spectra of chemical reference standards acquired on the same analytical platform**.** MSI Level 2: Metabolites identified by HILIC-MS* and spectrum similarity with public/commercial spectrum libraries. MSI Level 3: Metabolites putatively identified based on physicochemical characteristics of a chemical class of compounds or by spectrum similarity to known compounds. MSI Level 4: Unidentified or unclassified metabolites that can be differentiated or quantified based in spectrum data. Bolded values statistically differentiated the pairwise comparison of the two diagnosis groups.

|  | **Mass** | **RT*** | **Identity** | | | **ChemicalFormula** | | **DB identifier*** | | | **DHF/DSS Vs DF* p-value** | | **DHF/DSS Vs DF FC*** | **DHF/DSS Vs NEG* p-value** | | | **DHF/DSS Vs NEG* FC** | | **DF Vs NEG* p-value** | **DF Vs NEG FC*** | | |
| --- | --- | --- | --- | --- | --- | --- | --- | --- | --- | --- | --- | --- | --- | --- | --- | --- | --- | --- | --- | --- | --- | --- |
|  |  |  |  |  |  |  |  |  |  |  |  |  |  |  |  |  |  |  |  |  |  |  |
| **MSI level 1** | | | | | | | | | | | | | | | | | | | | | | |
|  | 115.0635 | 16.09 | L-Proline | | | C5H9NO2 | | HMDB00162 | | | **2.26E-01** | | **-2.15** | **6.65E-05** | | **-8.88** | | | **5.37E-03** | **-6.74** | | |
| ******* | 278.2245 | 1.1 | α-Linolenic acid | | | C18H30 O2 | | Metlin 192 | | | **1.20E-06** | | **10.94** | **7.82E-06** | | **10.01** | | | >0.05 | <2 | | |
| ****** | 302.2243 | 1.1 | Arachidonic Acid | | | C20H30O2 | | Metlin 35293 | | | **7.49E-03** | | **6.49** | **4.18E-02** | | **4.98** | | | >0.05 | <2 | | |
| ******* | 328.238 | 1.09 | Docosahexaenoic acid | | | C22H32O2 | | Kegg C06429 | | | **1.98E-04** | | **7.44** | **8.39E-05** | | **7.61** | | | >0.05 | <2 | | |
| ****** | 400.3312 | 1.25 | 1α-hydroxyvitamin D3 | | | C27H44O2 | | Metlin 42168 | | | **6.90E-02** | | **4.27** | **6.30E-02** | | **-3.76** | | | **9.91E-05** | **-8.03** | | |
| ****** | 416.3282 | 1.45 | 1,25-dihydroxyvitamin D3 | | | C27H44O3 | | Metlin 6382 | | | **5.33E-03** | | **-5.03** | **7.35E-03** | | **-4.96** | | | >0.05 | <2 | | |
| ****** | 495.3329 | 13.67 | LysoPC(16:0) | | | C24H50NO7P | | Metlin 61692 | | | **9.29E-03** | | **7.55** | **4.97E-03** | | **7.95** | | | >0.05 | <2 | | |
| ****** | 521.348 | 13.74 | LysoPC(18:1) | | | C26H52NO7P | | Metlin 61695 | | | **1.02E-03** | | **9.16** | **4.03E-04** | | **9.5** | | | >0.05 | <2 | | |
| **MSI Level 2** | | | | | | | | | | | | | | | | | | | | | | |
| ****** | 226.1932^****^ | 1.33 | Myristoleic acid | | | C14 H26 O2 | | NIST 1114112 | | | **3.21E-03** | | **-5.06** | **4.84E-02** | | <2 | | | **7.37E-03** | **-4.38** | | |
| ****** | 759.5778^****^ | 12.25 | Phosphatidylcholine (34:1) | | | C42 H82 NO8 P | | NIST 112638 | | | **6.82E-02** | | **3.76** | **1.16E-07** | | **-11.22** | | | **8.63E-04** | **-7.46** | | |
| ****** | 761.5934^****^ | 11.88 | Phosphatidylcholine(34:0) | | | C42H84NO8P | | NIST 112620 | | | **9.82E-02** | | **3.92** | **2.28E-05** | | **-9.84** | | | **1.32E-02** | **-5.92** | | |
| ****** | 771.5415 | 12.22 | Phosphatidylcholine (36:1) | | | C44H86NO7P | | NIST 1047871 | | | **3.70E-03** | | **6.9** | **2.27E-06** | | **-11.15** | | | **8.95E-02** | **-4.25** | | |
| **MSI Level 3** | | | | | | | | | | | | | | | | | | | | | | |
|  | **Mass** | **RT*** | | | **Potential ID** | | **Calculated Formula** | | **# DB hits*** | **DB identifier*** | **DHF/DSS Vs DF* p-value** | **DHF/DSS Vs DF FC*** | | **DHF/DSS Vs NEG* p-value** | | **DHF/DSS Vs NEG FC *** | | | **DF Vs NEG* p-value** | **DF Vs NEG FC*** | | |
|  |  |  |  |  |  |  |  |  |  |  |  |  |  |  |  |  |  |  |  |  |  |  |
|  |  |  |  |  |  |  |  |  |  |  |  |  |  |  |  |  |  |  |  |  |  |  |
|  |  |  |  |  |  |  |  |  |  |  |  |  |  |  |  |  |  |  |  |  |  |  |
|  | 113.0842 | 1.92 | | | Creatinine | | C4H7N3O | | 5 | HMDB00562 | >0.05 | 4.22 | | **1.19E-05** | | **11.98** | | | **1.27E-09** | **16.2** | | |
|  | 136.0383 | 6.21 | | | Hypoxanthine | | C5H4N4O | | >5 | Kegg C00262 | **2.58E-03** | **-7.16** | | **1.01E-03** | | **7.69** | | | >0.05 | <2 | | |
|  | 176.0837 | 2.18 | | | Ala-Ser | | C6H12N2O4 | | >5 | Metlin 85611 | >0.05 | 2.13 | | **2.09E-06** | | **10.34** | | | **1.02E-08** | **12.47** | | |
|  | 205.0594 | 1.24 | | | lipoamide | | C8H15NOS2 | | 2 | LMFA08010006 | >0.05 | <2 | | **1.22E-02** | | **-5.31** | | | **4.73E-04** | **-6.94** | | |
|  | 252.1127 | 1.49 | | | Phe Ser | | C12H16N2O4 | | >5 | Metlin 23673 | >0.05 | <2 | | **1.97E-06** | | **10.79** | | | **7.86E-08** | **12.19** | | |
| ****** | 253.2397 | 1.44 | | | Palmitoleamide | | C16H31NO | | 1 | Metlin 97431 | **3.43E-02** | **-4.57** | | >0.05 | | <2 | | | **7.57E-03** | **-5.38** | | |
|  | 255.1834 | 1.58 | | | N-decanoyl-L-homoserine lactone | | C14H25NO3 | | 1 | Metlin 45310 | **3.29E-02** | **4.49** | | **2.49E-03** | | **4.18** | | | **5.41E-07** | **8.67** | | |
|  | 276.2087 | 1.11 | | | 2,5-Octadecadiynoic acid | | C18H28O2 | | >5 | Kegg C16300 | **2.02E-05** | **8.38** | | >0.05 | | <2 | | | **2.93E-05** | **8.06** | | |
|  | 279.2558 | 1.4 | | | Linoleamide | | C18H33NO | | 1 | Metlin 43435 | **2.13E-02** | **-5.46** | | >0.05 | | <2 | | | **5.57E-03** | **-6.22** | | |
| ******* | 283.287 | 1.39 | | | Stearamide | | C18H37NO | | 1 | Metlin 34494 | >0.05 | <2 | | **1.07E-02** | | **6.05** | | | **2.81E-02** | **5.3** | | |
| ******* | 294.2196 | 1.1 | | | 9-OxoODE | | C18H30O3 | | >5 | Metlin 35860 | **1.01E-04** | **8.66** | | >0.05 | | <2 | | | **1.00E-03** | **7.31** | | |
|  | 296.1841 | 3.41 | | | Eicosatetraynoic acid | | C20H24O2 | | 1 | Metlin 62956 | >0.05 | <2 | | **3.63E-04** | | **6.65** | | | **8.54E-06** | **8.11** | | |
|  | 297.2667 | 1.47 | | | (d18:2) sphingosine | | C18H35NO2 | | >5 | Metlin 53911 | >0.05 | <2 | | **7.63E-03** | | **-5.2** | | | **9.77E-03** | **-4.6** | | |
| ******* | 300.2088 | 1.16 | | | 8,11,14-Eicosatetraynoic acid | | C20H28O2 | | >5 | Metlin 35290 | **2.91E-03** | **7.3** | | **8.43E-06** | | **-9.55** | | | >0.05 | -**2.25** | | |
|  | 302.2243 | 1.13 | | | 5,6-dehydro arachidonic acid | | C20H30O2 | | >5 | Metlin 35293 | **7.49E-03** | **6.49** | | >0.05 | | <2 | | | **4.18E-02** | **4.98** | | |
|  | 312.23 | 1.19 | | | 13(S)-HpODE | | C18H32O4 | | >5 | Metlin 35347 | **2.37E-03** | **6.93** | | >0.05 | | <2 | | | **8.65E-04** | **7.13** | | |
| ****** | 318.2209 | 1.22 | | | Leukotriene A4 | | C20H30O3 | | >5 | Kegg C00909 | **1.18E-03** | **7.06** | | **4.40E-03** | | **-5.64** | | | >0.05 | <2 | | |
| ******* | 324.2145 | 2.74 | | | 4,7,10,13-Docosatetraynoic acid | | C22H28O2 | | 1 | Metlin 74281 | >0.05 | <2 | | **3.39E-03** | | **6.39** | | | **1.05E-04** | **7.98** | | |
| ****** | 326.2242 | 1.09 | | | 4,5-dehydro docosahexaenoic acid | | C22H30O2 | | 5 | Metlin 62961 | **7.13E-03** | **5.8** | | **7.97E-04** | | **-6.84** | | | >0.05 | <2 | | |
|  | 346.1526 | 1.55 | | | Ala Ala Ala Asp | | C13H22N4O7 | | 1 | Metlin 103482 | >0.05 | <2 | | **2.63E-08** | | **-10.07** | | | **2.24E-09** | **-10.6** | | |
|  | 352.113 | 1.07 | | | Cys Gly Ser Ser | | C11H20N4O7S | | >5 | Metlin 113795 | >0.05 | -2.01 | | **1.46E-04** | | **7.95** | | | **3.34E-03** | **5.94** | | |
| ******* | 355.2564 | 3.03 | | | 6,9,12,15,18,21-Tetracosahexaenoic acid | | C15H31N8O2 | | >5 | HMDB13025 | >0.05 | <2 | | **1.00E-02** | | **5.33** | | | **3.77E-04** | **7.07** | | |
|  | 356.2911 | 1.17 | | | Heneicosanedioic acid | | C21H40O4 | | >5 | Metlin 35988 | >0.05 | <2 | | **6.32E-04** | | **7.02** | | | **3.86E-04** | **7.09** | | |
|  | 366.3279 | 1.18 | | | Alpha-Linoleoylcholine | | C23H44NO2 | | >5 | HMDB13213 | **1.63E-03** | **6.98** | | >0.05 | | <2 | | | **1.03E-02** | **5.79** | | |
|  | 368.3435 | 1.66 | | | 3-deoxyvitamin D3 | | C27H44 | | 2 | Metlin 42544 | **2.89E-03** | **8.37** | | >0.05 | | <2 | | | **6.98E-04** | **9.33** | | |
|  | 371.2517 | 3.6 | | | Lys Pro Lys | | C17H33N5O4 | | 3 | Metlin 16458 | >0.05 | **3.92** | | **1.26E-04** | | **7.03** | | | **1.05E-08** | **10.95** | | |
|  | 386.1757 | 1.23 | | | Cys His Lys | | C15H26N6O4S1 | | >5 | Metlin 16426 | >0.05 | **2.22** | | **1.63E-03** | | **-6.85** | | | **3.88E-02** | **-4.63** | | |
|  | 387.2462 | 5.19 | | | N-linoleoyl taurine | | C20H37NO4S | | >5 | LMFA08020138 | >0.05 | <2 | | **7.67E-04** | | **7.69** | | | **1.39E-04** | **8.28** | | |
|  | 414.204 | 1.21 | | | Ala Asp Ile Pro | | C18H30N4O7 | | >5 | Metlin 104472 | >0.05 | <2 | | **5.22E-03** | | **-4.55** | | | **6.12E-03** | **-5.32** | | |
|  | 415.2773 | 4.26 | | | Ala Lys Val Val | | C19H37N5O5 | | >5 | Metlin 107037 | **9.81E-02** | **3.93** | | **8.71E-04** | | **5.85** | | | **1.09E-07** | **9.78** | | |
|  | 428.2406 | 1.15 | | | Lys Pro Gln Gly | | C18H32N6O6 | | >5 | Metlin 172544 | **4.21E-03** | **-7.08** | | **1.99E-06** | | **10.28** | | | **2.07E-02** | **3.2** | | |
| ******* | 429.2929 | 3.74 | | | Arg Arg Val | | C20H39N5O5 | | >5 | Metlin 15788 | **7.65E-02** | **4.29** | | **8.71E-04** | | **5.89** | | | **8.18E-08** | **10.18** | | |
|  | 430.3758 | 1.27 | | | (+)-α-Tocopherol | | C29H50O2 | | >5 | Metlin 225 | **1.80E-04** | **-8.42** | | **1.30E-02** | | **4.98** | | | >0.05 | **-3.44** | | |
|  | 431.2722 | 5.89 | | | Ala Ile Lys Thr | | C19H37N5O6 | | >5 | Metlin 106456 | >0.05 | <2 | | **2.38E-04** | | **7.97** | | | **9.66E-04** | **6.91** | | |
|  | 445.267 | 1.15 | | | Trp Ile Lys | | C23H35N5O4 | | >5 | Metlin 16425 | **5.41E-03** | **-6.33** | | **5.83E-05** | | **8.27** | | | >0.05 | <2 | | |
|  | 448.2072 | 2.88 | | | Ala Cys Lys Gln | | C17H32N6O6S | | >5 | Metlin 104053 | >0.05 | **-3.42** | | **9.35E-05** | | **8.09** | | | **8.88E-03** | **4.67** | | |
|  | 455.3088 | 2.36 | | | 15S-HETE-dopamine | | C28H41NO4 | | >5 | LMFA08020151 | >0.05 | <2 | | **9.68E-05** | | **6.84** | | | **3.27E-07** | **8.49** | | |
|  | 459.3034 | 4.96 | | | Ile Ile Lys Ser | | C21H41N5O6 | | >5 | Metlin 162454 | >0.05 | **2.22** | | **8.84E-04** | | **5.58** | | | **9.22E-06** | **7.8** | | |
| ****** | 467.3008 | 13.97 | | | LysoPC(14:0) | | C22H46NO7P | | >5 | Metlin 61689 | >0.05 | -0.31 | | **8.78E-04** | | **-7.07** | | | **2.63E-04** | **-7.38** | | |
| ******* | 473.3198 | 4.4 | | | Ile Ile Thr Lys | | C22H43N5O6 | | >5 | Metlin 162455 | >0.05 | **3.05** | | **8.79E-04** | | **5.73** | | | **2.03E-06** | **8.78** | | |
|  | 477.2858 | 10.34 | | | LysoPE(18:2) | | C23H44NO7P | | >5 | HMDB11477 | **9.51E-02** | **2.45** | | **3.06E-06** | | **-9.22** | | | **1.57E-03** | **-6.77** | | |
|  | 478.3663 | 1.07 | | | 1α,25-dihydroxy-19-norvitamin D3 | | C29H50O5 | | >5 | Metlin 42477 | >0.05 | <2 | | **3.08E-03** | | **-5.63** | | | **4.72E-03** | **-5.3** | | |
| ****** | 481.3169 | 13.86 | | | PE(18:0) | | C23H48NO7P | | 2 | Metlin 40775 | >0.05 | **-2.15** | | **8.05E-03** | | **-5.67** | | | **2.62E-05** | **-7.82** | | |
|  | 490.2843 | 0.86 | | | Thr Lys Ser Arg | | C19H38N8O7 | | >5 | Metlin 234993 | **8.31E-02** | **3.66** | | **8.55E-02** | | **2.73** | | | **4.77E-04** | **6.39** | | |
|  | 499.3358 | 2.7 | | | Arg Ile Val Ile | | C23H45N7O5 | | >5 | Metlin 218436 | >0.05 | **2.17** | | **9.69E-05** | | **6.91** | | | **5.55E-08** | **9.08** | | |
|  | 501.2853 | 10.05 | | | PE(20:4) | | C25H44NO7P | | >5 | LMGP02050009 | **6.63E-02** | **3.12** | | **1.29E-04** | | **-7.38** | | | **4.26E-02** | **-4.26** | | |
|  | 509.3834 | 14.8 | | | LysoPC(O-20:0) | | C26H56NO6P | | >5 | LMGP01040041 | **9.46E-03** | **5.88** | | >0.05 | | <2 | | | **2.45E-02** | **5.05** | | |
|  | 517.3143 | 14 | | | PC(18:3) | | C26H48NO7P | | >5 | LMGP01050038 | >0.05 | <2 | | **1.01E-02** | | **6.18** | | | **8.29E-03** | **6.34** | | |
|  | 543.3626 | 3.08 | | | LysoPC(20:4) | | C28H50NO7P | | >5 | HMDB10395 | >0.05 | <2 | | **3.09E-04** | | **5.94** | | | **1.36E-06** | **7.83** | | |
|  | 557.3147 | 13.96 | | | LysoPC(20:4) | | C28H48NO8P | | >5 | Metlin 39147 | >0.05 | <2 | | **1.20E-02** | | **6.09** | | | **4.94E-03** | **6.68** | | |
|  | 571.328 | 12.61 | | | LysoPC(22:4) | | C30H54NO7P | | 2 | LMGP01050124 | **2.10E-02** | **-4.7** | | **7.90E-04** | | **6.99** | | | >0.05 | **2.28** | | |
|  | 573.3097 | 14.03 | | | PS(22:4) | | C28H48NO9P | | >5 | LMGP03050014 | >0.05 | <2 | | **1.97E-02** | | **4.82** | | | **2.34E-03** | **6.59** | | |
| ****** | 579.3536 | 13.23 | | | PS(22:1) | | C28H54NO9P | | >5 | LMGP03050023 | **1.88E-02** | **-4.47** | | **4.54E-05** | | **-7.81** | | | **4.19E-12** | **-12.28** | | |
|  | 584.2624 | 1.11 | | | Phe Gln Gln Tyr | | C28H36N6O8 | | 1 | Metlin 209358 | **2.29E-02** | **-4.93** | | >0.05 | | <2 | | | **1.01E-03** | **-6.67** | | |
|  | 586.3006 | 1.13 | | | Arg Gln His Phe | | C26H38N10O6 | | >5 | Metlin 220803 | **1.14E-02** | **-5.42** | | **2.01E-06** | | **8.85** | | | **6.03E-03** | **3.43** | | |
|  | 588.4753 | 1.07 | | | DG(34:4) | | C37H64O5 | | >5 | Metlin 4282 | >0.05 | <2 | | **3.87E-04** | | **-5.44** | | | **5.38E-03** | **-4.59** | | |
|  | 591.3255 | 1.11 | | | Cer(38:2) | | C38H73NO3 | | >5 | LMSP02010026 | >0.05 | <2 | | **2.38E-03** | | **4.27** | | | **2.57E-05** | **5.89** | | |
|  | 603.3249 | 1.12 | | | Trp Ser Arg Arg | | C26H41N11O6 | | >5 | Metlin 253773 | >0.05 | **-2.01** | | **1.98E-06** | | **9.31** | | | **2.31E-06** | **7.3** | | |
| ****** | 635.4143 | 12.79 | | | PE(28:0) | | C33H66NO8P | | >5 | Metlin 40418 | >0.05 | <2 | | **1.21E-02** | | **-3.56** | | | **4.22E-03** | **-3.35** | | |
|  | 642.3612 | 1.11 | | | PI(21:0) | | C30H59O12P | | >5 | LMGP06050025 | >0.05 | <2 | | **1.98E-06** | | **10.07** | | | **6.21E-07** | **9.49** | | |
|  | 646.4514 | 1.26 | | | PA(14:0/18:1(9Z)) | | C35H67O8P | | >5 | Metlin 40917 | **3.46E-02** | **-4.89** | | >0.05 | | <2 | | | **5.90E-03** | **-6** | | |
| ******* | 647.6205 | 1.71 | | | Nervonic Ceramide | | C42H81NO3 | | >5 | Metlin 63014 | **2.04E-03** | **-6.91** | | **5.61E-04** | | **7.68** | | | >0.05 | <2 | | |
|  | 649.3669 | 1.16 | | | PS(12:0/14:1(9Z)) | | C32H60NO10P | | 3 | LMGP03010046 | **2.29E-03** | **-6.64** | | **1.94E-06** | | **9.12** | | | **3.48E-02** | **2.48** | | |
|  | 660.4764 | 1.05 | | | DG(40:10) | | C43H64O5 | | >5 | HMDB07353 | **8.29E-04** | **5.28** | | **6.07E-03** | | **-3.93** | | | >0.05 | <2 | | |
|  | 668.5531 | 1.27 | | | DG(40:6) | | C43H72O5 | | >5 | Metlin 4560 | >0.05 | **3.68** | | **1.85E-04** | | **-7.13** | | | **4.33E-02** | **-3.44** | | |
|  | 670.568 | 1.47 | | | CE(20:5) | | C47H74O2 | | 1 | Metlin 58532 | >0.05 | <2 | | **1.44E-05** | | **-8.78** | | | **3.46E-08** | **-10.64** | | |
| ****** | 672.5202 | 14.37 | | | SM(d18:2/14:0) | | C37H73N2O6P | | 2 | Metlin 83736 | **5.28E-02** | **-4.17** | | **3.87E-02** | | **-4.58** | | | **1.66E-05** | **-8.76** | | |
|  | 699.549 | 2.32 | | | GlcCer(d34:1) | | C40H77NO8 | | >5 | Metlin 41606 | >0.05 | <2 | | **4.24E-03** | | **-6.5** | | | **2.39E-02** | **-5.06** | | |
|  | 731.6208 | 1.82 | | | PC(O-34:1) | | C42H86NO6P | | >5 | Metlin 40202 | >0.05 | **2.13** | | **2.92E-04** | | **-7.83** | | | **7.90E-03** | **-5.7** | | |
| ******* | 753.5297 | 12.13 | | | PC(34:4) | | C42H76NO8P | | 5 | LMGP01010506 | >0.05 | **-2.38** | | **8.19E-02** | | **-2.6** | | | **2.26E-03** | **-4.98** | | |
| ******* | 755.548 | 12.18 | | | PC(34:3) | | C42H78NO8P | | >5 | HMDB07881 | >0.05 | <2 | | **1.71E-02** | | **-5.97** | | | **6.94E-03** | **-6.65** | | |
| ****** | 763.5561 | 1.13 | | | PC(36:5) | | C44H78NO7P | | >5 | Metlin 59706 | >0.05 | **2.02** | | **1.93E-05** | | **-7.95** | | | **1.62E-03** | **-5.93** | | |
|  | 771.5415 | 12.32 | | | PS(36:3) | | C42H78NO9P | | >5 | LMGP03020029 | **3.70E-03** | **6.9** | | **2.27E-06** | | **-11.15** | | | **8.95E-02** | **-4.25** | | |
| ****** | 773.5572 | 12.24 | | | PS(36:2) | | C45H76NO7P | | 5 | LMGP03020013 | >0.05 | <2 | | **1.24E-03** | | **-2.92** | | | **8.72E-03** | **-3.07** | | |
| ******* | 777.5311 | 11.87 | | | PC(38:6) | | C44H76NO8P | | 5 | HMDB07892 | **9.06E-03** | **-5.57** | | >0.05 | | **-2.6** | | | **1.13E-05** | **-8.18** | | |
| ******* | 779.5478 | 11.88 | | | PC(36:5) | | C44H78NO8P | | >5 | HMDB07890 | >0.05 | <2 | | **1.83E-06** | | **-10.82** | | | **2.86E-06** | **-10.46** | | |
| ****** | 783.5787 | 12.26 | | | PC(36:3) | | C44H82NO8P | | >5 | HMDB07921 | **9.82E-02** | **3.92** | | **2.28E-05** | | **-9.84** | | | **1.32E-02** | **-5.92** | | |
|  | 789.5524 | 11.59 | | | PS(36:1) | | C42H80NO10P | | >5 | HMDB10163 | **2.11E-02** | **-2.77** | | **1.14E-04** | | **-1.78** | | | **1.76E-04** | **-4.55** | | |
| ******* | 799.5714 | 12.06 | | | PS(38:3) | | C44H82NO9P | | >5 | LMGP03020036 | >0.05 | <2 | | **1.37E-05** | | **-9.69** | | | **3.20E-04** | **-7.9** | | |
| ******* | 805.5633 | 11.78 | | | PC(36:6) | | C46H80NO8P | | >5 | Metlin 39390 | >0.05 | **3.11** | | **3.95E-05** | | **-8.82** | | | **1.24E-02** | **-5.72** | | |
| ****** | 807.5786 | 12.24 | | | PC(38:5) | | C46H82NO8P | | >5 | HMDB07989 | >0.05 | **-2.85** | | **4.25E-02** | | **-5.03** | | | **1.75E-03** | **-7.88** | | |
| ****** | 813.5517 | 11.98 | | | PS(38:3) | | C44H80NO10P | | >5 | LMGP03010224 | >0.05 | **-2.3** | | **9.16E-05** | | **-8.72** | | | **6.50E-07** | **-11.02** | | |
| ****** | 815.567 | 11.5 | | | PS(38:2) | | C44H82NO10P | | >5 | LMGP03010200 | **1.91E-02** | **-4.85** | | **9.76E-04** | | **-6.1** | | | **2.76E-08** | **-10.95** | | |
| ****** | 819.5418 | 11.83 | | | PS(40:6) | | C46H78NO9P | | >5 | LMGP03030089 | >0.05 | <2 | | **3.96E-10** | | **-13.45** | | | **1.15E-09** | **-12.98** | | |
|  | 821.5579 | 11.73 | | | PS(40:6) | | C46H80NO9P | | 1 | LMGP03020083 | >0.05 | **4.09** | | **1.54E-07** | | **-12.48** | | | **2.18E-04** | **-8.38** | | |
|  | 823.5718 | 11.84 | | | PS(40:5) | | C46H82NO9P | | 2 | LMGP03020064 | **7.43E-02** | **4.89** | | **1.28E-06** | | **-11.86** | | | **2.51E-03** | **-6.97** | | |
|  | 824.6873 | 1.42 | | | TG(50:5) | | C53H92O6 | | 4 | LMGL03010056 | **1.36E-02** | **2.6** | | **3.58E-05** | | **-6.49** | | | **2.45E-02** | **-3.89** | | |
| ******* | 825.5869 | 12.01 | | | PS(40:4) | | C46H84NO9P | | >5 | LMGP03020040 | >0.05 | **3.92** | | **1.14E-04** | | **-9.29** | | | **1.46E-02** | **-5.36** | | |
|  | 826.7007 | 1.81 | | | TG(50:4) | | C53H94O6 | | 4 | LMGL03010046 | **4.61E-03** | **5.16** | | **7.71E-04** | | **-6.33** | | | >0.05 | <2 | | |
| ****** | 837.5519 | 11.81 | | | PS(40:5) | | C46H80NO10P | | >5 | LMGP03010339 | >0.05 | <2 | | **3.42E-06** | | **-11.32** | | | **2.54E-05** | **-10.09** | | |
| ****** | 841.5815 | 11.35 | | | PS(40:3) | | C46H84NO10P | | >5 | LMGP03010338 | >0.05 | <2 | | **2.56E-05** | | **-9.12** | | | **2.18E-07** | **-10.54** | | |
|  | 848.6875 | 1.18 | | | PG(42:0) | | C48H97O9P | | >5 | LMGP04020069 | **3.57E-03** | **5.79** | | **1.67E-02** | | **-4.39** | | | >0.05 | <2 | | |
|  | 849.5879 | 11.79 | | | PS(42:6) | | C48H84NO9P | | >5 | LMGP03020093 | >0.05 | **2.76** | | **8.77E-06** | | **-10.48** | | | **1.05E-03** | **-7.72** | | |
|  | 852.7204 | 1.84 | | | TG(52:5) | | C55H96O6 | | 1 | LMGL03010140 | **6.64E-02** | **4.27** | | **4.91E-06** | | **-9.12** | | | **1.33E-02** | **-4.85** | | |
| ****** | 863.5598 | 11.81 | | | PS(44:6) | | C48H82NO10P | | >5 | LMGP03010587 | >0.05 | <2 | | **1.17E-06** | | **-9.79** | | | **4.30E-07** | **-9.82** | | |
|  | 872.6875 | 1.08 | | | TG(54:9) | | C57H92O6 | | >5 | Metlin 37005 | **4.18E-04** | **7.22** | | **1.31E-05** | | **-7.82** | | | >0.05 | <2 | | |
|  | 874.7047 | 1.35 | | | TG(54:8) | | C57H94O6 | | >5 | Metlin 36949 | **5.13E-02** | **3.53** | | **9.50E-05** | | **-7.87** | | | **4.79E-02** | **-4.34** | | |
|  | 876.719 | 1.27 | | | TG(54:7) | | C57H96O6 | | >5 | LMGL03010398 | **1.80E-02** | **5.39** | | **1.75E-05** | | **-8.58** | | | >0.05 | **-3.19** | | |
|  | 880.7504 | 2.4 | | | TG(54:5) | | C57H100O6 | | >5 | Metlin 36812 | **7.51E-04** | **7.11** | | **4.17E-03** | | **-5.73** | | | >0.05 | <2 | | |
| ****** | 887.5658 | 11.93 | | | PS(44:8) | | C50H82NO10P | | >5 | LMGP03010786 | **8.66E-02** | **3.05** | | **8.04E-04** | | **-6.93** | | | **8.41E-02** | **-3.88** | | |
|  | 900.7174 | 1.36 | | | TG(56:9) | | C59H96O6 | | 3 | LMGL03010869 | >0.05 | <2 | | **8.02E-06** | | **-8.43** | | | **1.10E-03** | **-6.55** | | |
|  | 916.7133 | 1.17 | | | TG(57:8) | | C60H100O6 | | >5 | Metlin 37434 | **4.85E-02** | **3.8** | | **7.24E-04** | | **-6.27** | | | 2.44E-01 | **-2.47** | | |
| **MSI Level 4** | | | | | | | | | | | | | | | | | | | | | |  |
|  | **Mass** | **RT*** | | **Potential ID** | | | **Calculated Formula** | | | | **DHF/DSS Vs DF* p-value** | **DHF/DSS Vs DF FC *** | | **DHF/DSS Vs NEG* p-value** | **DHF/DSS Vs NEG FC*** | | | **DF Vs NEG* p-value** | | | **DF Vs NEG FC *** |  |
|  |  |  |  |  |  |  |  |  |  |  |  |  |  |  |  |  |  |  |  |  |  |  |
| ****** | 110.1097 | 1.72 | | Unidentified | | | C8 H14 | | | | >0.05 | <2 | | **1.30E-03** | **-6.07** | | | **8.10E-04** | | | **-6.24** |  |
|  | 127.0997 | 2.3 | | Unidentified | | | C7H13NO | | | | >0.05 | **3.92** | | **8.85E-06** | **10.36** | | | **1.20E-09** | | | **14.28** |  |
|  | 128.0948 | 2.9 | | Unidentified | | | C6H12N2O | | | | >0.05 | **3.67** | | **8.94E-06** | **9.91** | | | **1.06E-09** | | | **13.57** |  |
|  | 147.0174 | 1.44 | | Unidentified | | | C4H5NO5 | | | | **1.79E-02** | **5.01** | | **7.33E-03** | **3.38** | | | **2.69E-06** | | | **8.39** |  |
|  | 155.1308 | 1.8 | | Unidentified | | | C9H17NO | | | | >0.05 | **3.54** | | **5.48E-05** | **8.03** | | | **3.20E-09** | | | **11.56** |  |
| ****** | 164.1045 | 2.2 | | Unidentified | | | C8H12N4 | | | | >0.05 | <2 | | **1.78E-06** | **8.78** | | | **2.76E-05** | | | **7.34** |  |
| ******* | 166.0394 | 1.49 | | Unidentified | | | C7H6N2O3 | | | | >0.05 | **3.28** | | **3.11E-04** | **6.61** | | | **4.18E-07** | | | **9.89** |  |
| ******* | 168.0785 | 1.26 | | Unidentified | | | C5H8N6O | | | | >0.05 | <2 | | **4.44E-07** | **11.64** | | | **1.09E-08** | | | **13.38** |  |
|  | 169.1463 | 1.68 | | Unidentified | | | C10H19NO | | | | >0.05 | **3.64** | | **6.36E-03** | **4.51** | | | **1.02E-05** | | | **8.15** |  |
|  | 183.1618 | 1.59 | | Unidentified | | | C11H21NO | | | | >0.05 | **2.81** | | **1.52E-02** | **5.05** | | | **1.64E-04** | | | **7.85** |  |
|  | 196.1574 | 1.91 | | Unidentified | | | C11H20N2O | | | | >0.05 | **2.55** | | **2.08E-06** | **10.14** | | | **1.20E-09** | | | **12.69** |  |
|  | 214.1203 | 1.23 | | Unidentified | | | C11H18O4 | | | | >0.05 | <2 | | **1.92E-06** | **10.76** | | | **9.84E-09** | | | **12.69** |  |
| ******* | 222.1464 | 2.2 | | Unidentified | | | C17H18 | | | | >0.05 | <2 | | **4.52E-02** | **4.79** | | | **3.79E-03** | | | **6.39** |  |
| ****** | 225.1732 | 1.47 | | Unidentified | | | C13H23NO2 | | | | **5.05E-02** | **-4.19** | | **5.39E-02** | **-4.13** | | | **4.52E-05** | | | **-8.32** |  |
|  | 232.1308 | 1.23 | | Unidentified | | | C11H20O5 | | | | >0.05 | <2 | | **1.91E-06** | **10.78** | | | **9.90E-09** | | | **12.67** |  |
|  | 236.1621 | 2.02 | | Unidentified | | | C11H24O5 | | | | >0.05 | <2 | | **8.84E-03** | **5.74** | | | **1.72E-02** | | | **5.17** |  |
| ******* | 258.1969 | 1.14 | | Unidentified | | | C16H24N3 | | | | **3.36E-04** | **6.76** | | **3.13E-02** | **-3.21** | | | **7.36E-02** | | | **3.55** |  |
|  | 267.2041 | 2.19 | | Unidentified | | | C11H23N8 | | | | >0.05 | <2 | | **1.23E-02** | **6.02** | | | **6.95E-03** | | | **6** |  |
|  | 294.204 | 2.17 | | Unidentified | | | C14H30O6 | | | | >0.05 | <2 | | **6.99E-04** | **6.85** | | | **7.32E-04** | | | **6.71** |  |
|  | 295.2869 | 1.42 | | Unidentified | | | C19H37NO | | | | >0.05 | <2 | | **1.88E-06** | **9.64** | | | **1.29E-08** | | | **10.95** |  |
|  | 299.2091 | 1.85 | | Unidentified | | | C16H29NO4 | | | | >0.05 | <2 | | **9.69E-06** | **8.02** | | | **3.12E-05** | | | **7.31** |  |
| ******* | 308.2199 | 1.99 | | Unidentified | | | C15H32O6 | | | | >0.05 | <2 | | **1.79E-02** | **5.19** | | | **4.09E-03** | | | **5.97** |  |
|  | 310.1989 | 3.04 | | Unidentified | | | C14H30O7 | | | | >0.05 | <2 | | **1.67E-04** | **8.19** | | | **1.10E-05** | | | **9.42** |  |
|  | 311.2304 | 2.61 | | Unidentified | | | C13H27N8O | | | | >0.05 | <2 | | **4.56E-02** | **4.57** | | | **3.19E-03** | | | **6.13** |  |
|  | 313.2095 | 3.42 | | Unidentified | | | C15H27N3O4 | | | | >0.05 | <2 | | **4.44E-04** | **5.69** | | | **1.19E-05** | | | **7.21** |  |
| ****** | 314.1271 | 1.18 | | Unidentified | | | C21H30O2 | | | | >0.05 | <2 | | **2.14E-10** | **-11.54** | | | **7.06E-08** | | | **-10.34** |  |
|  | 321.3023 | 1.4 | | Unidentified | | | C19H37N4 | | | | >0.05 | <2 | | **9.15E-06** | **7.24** | | | **2.94E-05** | | | **6.72** |  |
|  | 323.3184 | 1.37 | | Unidentified | | | C21H41NO | | | | >0.05 | **2.1** | | **2.51E-03** | **6.91** | | | **3.32E-06** | | | **9.01** |  |
|  | 325.2457 | 2.37 | | Unidentified | | | C22H31NO | | | | >0.05 | <2 | | **1.16E-02** | **5.73** | | | **1.89E-03** | | | **6.63** |  |
|  | 327.2253 | 3.02 | | Unidentified | | | C13H27N8O2 | | | | >0.05 | <2 | | **1.37E-07** | **10.83** | | | **3.36E-08** | | | **10.88** |  |
|  | 332.2338 | 1.15 | | Unidentified | | | C21H32O3 | | | | >0.05 | <2 | | **1.14E-05** | **-9.32** | | | **7.27E-07** | | | **-9.88** |  |
| ******* | 338.2302 | 2.49 | | Unidentified | | | C16H34O7 | | | | >0.05 | **2.44** | | **1.07E-03** | **6.19** | | | **3.12E-06** | | | **8.63** |  |
|  | 343.22 | 4.5 | | Unidentified | | | C13H27N8O3 | | | | >0.05 | <2 | | **5.23E-04** | **7.86** | | | **1.07E-05** | | | **9.31** |  |
|  | 349.3334 | 1.38 | | Unidentified | | | C21H41N4 | | | | >0.05 | <2 | | **1.88E-06** | **9.34** | | | **1.04E-08** | | | **10.96** |  |
|  | 351.3481 | 1.42 | | Unidentified | | | C21H43N4 | | | | >0.05 | <2 | | **8.15E-06** | **9.04** | | | **9.07E-08** | | | **10.49** |  |
|  | 354.0642 | 1.05 | | Unidentified | | | C21H10N2O4 | | | | **8.93E-03** | **5.12** | | **1.07E-05** | **-7.51** | | | >0.05 | | | **-2.39** |  |
|  | 357.2358 | 4.09 | | Unidentified | | | C14H29N8O3 | | | | >0.05 | **2.92** | | **5.43E-03** | **4.23** | | | **1.75E-05** | | | **7.15** |  |
|  | 359.96 | 1.37 | | Unidentified | | | C12N4O10 | | | | **8.09E-02** | **4.61** | | **2.61E-05** | **-9.15** | | | **7.34E-03** | | | **-4.53** |  |
|  | 364.3121 | 1.22 | | Unidentified | | | C27H40 | | | | **7.13E-02** | **4.23** | | **3.13E-05** | **-8.27** | | | **1.97E-02** | | | **-4.04** |  |
|  | 374.1699 | 2.1 | | Unidentified | | | C21H26O6 | | | | **5.16E-02** | **-4.3** | | **5.80E-04** | **6.71** | | | >0.05 | | | **2.41** |  |
| ******* | 385.2669 | 3.18 | | Unidentified | | | C24H35NO3 | | | | **7.07E-02** | **4.25** | | **3.17E-04** | **6.34** | | | **1.08E-08** | | | **10.59** |  |
|  | 398.2428 | 1.38 | | Unidentified | | | C21H30N6O2 | | | | >0.05 | <2 | | **2.20E-02** | **3.09** | | | **1.83E-02** | | | **3.16** |  |
|  | 411.2839 | 2.09 | | Unidentified | | | C20H37N5O4 | | | | >0.05 | <2 | | **1.23E-04** | **6.4** | | | **1.20E-05** | | | **7.25** |  |
|  | 423.3549 | 1.52 | | Unidentified | | | C21H43N8O | | | | >0.05 | <2 | | **1.89E-02** | **5.33** | | | **4.07E-03** | | | **6.37** |  |
|  | 444.3595 | 1.08 | | Unidentified | | | C29H48O3 | | | | **7.06E-02** | **2.45** | | **7.77E-03** | **-3.62** | | | **6.04E-04** | | | **-1.17** |  |
|  | 460.3505 | 1.07 | | Unidentified | | | C29H48O4 | | | | **7.02E-02** | **-3.5** | | **1.07E-04** | **6.33** | | | **5.20E-02** | | | **2.82** |  |
|  | 475.2991 | 6.62 | | Unidentified | | | C31H41NO3 | | | | >0.05 | <2 | | **3.63E-03** | **5.81** | | | **2.42E-02** | | | **4.41** |  |
| ******* | 483.3403 | 2.03 | | Unidentified | | | C22H43N8O4 | | | | >0.05 | <2 | | **8.78E-04** | **5.18** | | | **1.48E-05** | | | **6.78** |  |
|  | 494.3609 | 1.06 | | Unidentified | | | C29H50O6 | | | | >0.05 | <2 | | **5.71E-03** | **-5.86** | | | **6.12E-04** | | | **-6.77** |  |
|  | 506.4178 | 1.56 | | Unidentified | | | C31H54O5 | | | | >0.05 | <2 | | **1.12E-02** | **-5.11** | | | **2.78E-02** | | | **-4.35** |  |
|  | 513.3508 | 2.48 | | Unidentified | | | C23H45N8O5 | | | | >0.05 | **3.08** | | **8.63E-04** | **5.66** | | | **6.16E-07** | | | **8.74** |  |
|  | 519.4282 | 0.57 | | Unidentified | | | C32H57NO4 | | | | >0.05 | <2 | | **1.00E-02** | **-3.12** | | | **8.71E-04** | | | **-3.61** |  |
|  | 527.3674 | 2.27 | | Unidentified | | | C23H45N9O5 | | | | >0.05 | <2 | | **3.15E-04** | **6.07** | | | **3.26E-06** | | | **7.83** |  |
|  | 529.28 | 9.23 | | Unidentified | | | C24H35N9O5 | | | | >0.05 | <2 | | **9.48E-03** | **-5.23** | | | **2.69E-03** | | | **-5.85** |  |
| ****** | 534.4486 | 1.54 | | Unidentified | | | C28H60N3O6 | | | | >0.05 | <2 | | **2.20E-02** | **-2.96** | | | **4.50E-03** | | | **-4.36** |  |
|  | 545.5164 | 1.35 | | Unidentified | | | C34H65N4O | | | | >0.05 | **-2.09** | | **3.10E-02** | **-3.29** | | | **1.29E-03** | | | **-5.37** |  |
|  | 547.5298 | 1.42 | | Unidentified | | | C32H65N7 | | | | **5.01E-03** | **-5.31** | | >0.05 | **2.38** | | | >0.05 | | | **-2.93** |  |
| ****** | 550.4438 | 1.69 | | Unidentified | | | C34H62O5 | | | | >0.05 | **2.41** | | **2.57E-03** | **-5.46** | | | >0.05 | | | **-3.05** |  |
|  | 551.4235 | 1.16 | | Unidentified | | | C29H62NO6P | | | | **2.59E-04** | **-7.51** | | >0.05 | <2 | | | **4.23E-05** | | | **-8.04** |  |
|  | 567.4191 | 1.18 | | Unidentified | | | C38H53N3O | | | | **1.89E-03** | **-6.96** | | >0.05 | <2 | | | **3.21E-04** | | | **-7.43** |  |
|  | 574.4938 | 1.15 | | Unidentified | | | C33H62N6O2 | | | | **1.60E-02** | **-5.31** | | >0.05 | <2 | | | **8.40E-03** | | | **-5.35** |  |
|  | 614.3299 | 1.12 | | Unidentified | | | C44H42N2O | | | | >0.05 | <2 | | **1.97E-06** | **9.8** | | | **8.22E-06** | | | **7.87** |  |
|  | 619.3852 | 13.01 | | Unidentified | | | C28H55N6O7S | | | | >0.05 | **-2.32** | | **1.22E-03** | **-7.34** | | | **1.65E-06** | | | **-9.67** |  |
|  | 627.1869 | 1.02 | | Unidentified | | | C34H25N7O6 | | | | >0.05 | <2 | | **1.96E-02** | **-3.19** | | | **6.15E-04** | | | **-4.84** |  |
|  | 630.796 | 1.38 | | Unidentified | | | C44H86O | | | | >0.05 | <2 | | **4.09E-05** | **-10.87** | | | **3.61E-05** | | | **-11.08** |  |
|  | 631.3566 | 1.12 | | Unidentified | | | C34H45N7O5 | | | | >0.05 | <2 | | **1.98E-06** | **10.49** | | | **1.21E-08** | | | **10.97** |  |
|  | 633.641 | 2.04 | | Unidentified | | | C40H81N4O | | | | >0.05 | <2 | | **4.34E-02** | **-4.01** | | | **6.64E-03** | | | **-5.1** |  |
|  | 645.7947 | 1.36 | | Unidentified | | | C44H85O2 | | | | >0.05 | **3.6** | | **3.90E-04** | **-6.86** | | | **3.99E-02** | | | **-3.26** |  |
|  | 659.3884 | 1.11 | | Unidentified | | | C32H51N8O7 | | | | >0.05 | <2 | | **1.97E-06** | **10.82** | | | **1.12E-08** | | | **11.59** |  |
| ****** | 662.5596 | 1.38 | | Unidentified | | | C39H68N9 | | | | >0.05 | **3.26** | | **7.60E-06** | **-8.53** | | | **6.40E-03** | | | **-5.27** |  |
|  | 677.5575 | 1.05 | | Unidentified | | | C38H77O9 | | | | **1.71E-02** | **4.9** | | **2.27E-04** | **-7.2** | | | >0.05 | | | **-2.29** |  |
|  | 681.3565 | 1.05 | | Unidentified | | | C44H47N3O4 | | | | >0.05 | <2 | | **3.36E-03** | **-4.94** | | | **1.68E-04** | | | **-6.2** |  |
|  | 687.419 | 1.1 | | Unidentified | | | C48H53N3O | | | | >0.05 | <2 | | **2.00E-06** | **9.79** | | | **2.59E-06** | | | **8.57** |  |
|  | 701.2051 | 1.02 | | Unidentified | | | C44H31NO8 | | | | >0.05 | <2 | | **1.20E-02** | **-2.63** | | | **9.59E-04** | | | **-4.19** |  |
|  | 707.6204 | 1.7 | | Unidentified | | | C45H79N4O2 | | | | >0.05 | **2.96** | | **1.16E-07** | **-10.97** | | | **1.14E-04** | | | **-8.01** |  |
|  | 709.6356 | 1.64 | | Unidentified | | | C45H81N4O2 | | | | >0.05 | **3.5** | | **4.25E-03** | **-6.62** | | | >0.05 | | | **-3.12** |  |
|  | 723.6134 | 1.49 | | Unidentified | | | C44H83O7 | | | | **5.50E-02** | **3.15** | | **1.12E-03** | **-6.09** | | | >0.05 | | | **-2.94** |  |
|  | 726.8528 | 1.92 | | Unidentified | | | C50H98N2 | | | | **9.90E-02** | **3.69** | | **2.25E-03** | **4.71** | | | **2.21E-06** | | | **8.41** |  |
|  | 742.9198 | 1.34 | | Unidentified | | | C45HN3O10 | | | | >0.05 | **3.05** | | **2.13E-10** | **-11.89** | | | **2.71E-07** | | | **-8.84** |  |
|  | 749.5224 | 1.12 | | Unidentified | | | C39H76NO10P | | | | >0.05 | <2 | | **1.08E-04** | **-7.27** | | | **2.50E-03** | | | **-5.65** |  |
|  | 756.3411 | 1.05 | | Unidentified | | | C46H48N2O8 | | | | **6.53E-03** | **-4.75** | | **9.87E-05** | **6.42** | | | >0.05 | | | <2 |  |
|  | 757.2136 | 1.27 | | Unidentified | | | C39H31N7O10 | | | | >0.05 | **-2.29** | | **3.49E-02** | **-2.47** | | | **3.27E-04** | | | **-4.76** |  |
|  | 760.2125 | 1.28 | | Unidentified | | | C52H24N8 | | | | **7.59E-02** | **-3.31** | | >0.05 | **-2.04** | | | **6.33E-04** | | | **-5.35** |  |
|  | 770.9133 | 1.45 | | Unidentified | | | C51HNO9 | | | | >0.05 | <2 | | **1.81E-03** | **5.23** | | | **3.87E-03** | | | **4.56** |  |
|  | 775.224 | 1.02 | | Unidentified | | | C54H27N6O | | | | >0.05 | <2 | | **1.84E-02** | **-2.37** | | | **1.61E-03** | | | **-3.8** |  |
| ****** | 781.8466 | 1.28 | | Unidentified | | | C45H99N9O | | | | >0.05 | <2 | | **9.70E-04** | **5.02** | | | **7.73E-06** | | | **6.98** |  |
|  | 801.9023 | 1.24 | | Unidentified | | | C50N3O10 | | | | **4.57E-02** | **3.14** | | >0.05 | <2 | | | **2.35E-03** | | | **4.27** |  |
|  | 814.8132 | 1.6 | | Unidentified | | | C49H100N9 | | | | >0.05 | **-3.09** | | **1.09E-05** | **7.57** | | | **1.92E-03** | | | **4.47** |  |
| ******* | 833.5939 | 12.4 | | Unidentified | | | C48H84NO8P | | | | >0.05 | <2 | | **1.28E-02** | **-7.58** | | | **1.07E-02** | | | **-7.52** |  |
|  | 845.4772 | 1.08 | | Unidentified | | | C55H63N3O5 | | | | **2.59E-03** | **-6.04** | | **2.11E-06** | **8.51** | | | **1.88E-02** | | | **2.47** |  |
|  | 856.9502 | 1.47 | | Unidentified | | | C58HO10 | | | | >0.05 | <2 | | **2.00E-03** | **5.66** | | | **2.90E-04** | | | **6.45** |  |
|  | 857.4688 | 11.71 | | Unidentified | | | C46H63N7O9 | | | | **4.55E-02** | **3.05** | | **3.50E-08** | **-10.52** | | | **8.84E-05** | | | **-7.47** |  |
|  | 873.5066 | 1.08 | | Unidentified | | | C55H65N6O4 | | | | **1.99E-02** | **-4.83** | | **2.08E-06** | **8.64** | | | **1.86E-03** | | | **3.81** |  |
|  | 893.559 | 11.95 | | Unidentified | | | C58H69N8O | | | | **7.47E-02** | **-3.85** | | >0.05 | <2 | | | **3.54E-03** | | | **-5.79** |  |
|  | 899.5378 | 11.72 | | Unidentified | | | C61H67N6O | | | | **5.84E-02** | **2.93** | | **7.29E-06** | **-9.05** | | | **4.37E-03** | | | **-6.12** |  |
|  | 937.0865 | 1.42 | | Unidentified | | | C73H13O3 | | | | >0.05 | <2 | | **3.50E-05** | **7.1** | | | **3.33E-06** | | | **7.36** |  |
|  | 951.105 | 1.33 | | Unidentified | | | C77H13N | | | | >0.05 | <2 | | **9.80E-04** | **-7.88** | | | **2.99E-03** | | | **-6.46** |  |
|  | 961.6624 | 1 | | Unidentified | | | C56H89N4O9 | | | | >0.05 | <2 | | **9.87E-05** | **5.28** | | | **3.42E-03** | | | **3.38** |  |
|  | 975.9779 | 1.41 | | Unidentified | | | C60N8O9 | | | | >0.05 | <2 | | **1.08E-02** | **-3.85** | | | **7.27E-03** | | | **-3.79** |  |
|  | 979.1335 | 1.33 | | Unidentified | | | C76H19O3 | | | | >0.05 | <2 | | **6.50E-04** | **-5.68** | | | **1.01E-03** | | | **-5.31** |  |
|  | 999.0873 | 1.28 | | Unidentified | | | C74H15O6 | | | | >0.05 | **2.79** | | **2.85E-03** | **-5.73** | | | **2.87E-02** | | | **-2.94** |  |

^*^Abbreviations DHF/DSS - dengue hemorrhagic fever/dengue shock syndrome; DF - dengue fever; ND - non-dengue febrile disease; HILIC-MS/MS - hydrophilic interaction chromatography tandem mass spectrometry; MRM LC-MS/MS - multiple reaction monitoring LC-MS/MS; RT - retention time; FC - fold change; DB – database; MSI – Metabolomics Standard Initiative; HMDB - human metabolome database; LMGP - Lipid maps gateway; MetLin - Metabolite and Tandem Mass Spectrometry Database; KEGG- Kyoto Encyclopedia of Genes and Genomes, NIST - National Institute of Standards and Technology; lysoPC: lysophosphatidylcholine; PS – phosphatidylserine; PC – phosphatidylcholine; DG – diacylglycerol; TG – triglyceride; HETE – hydroxyeicosatetraenoic acid; PE – phosphatidylethanolamine; PA–phosphatidic acid; Cer – ceramide; PI – phosphatidylinositol; CE – cholesteryl ester; SM– sphingomyelin.
^**^ Metabolies also detected in Mexican samples with concordant fold change directions.
^***^ Metabolites also detected in Mexican samples but with non-concordant fold change directions.
^****^Ionized form of metabolites: myristoleic acid [M+H-H2O]^+^ = 226.1932; phosphatidylcholine (34:1) [M+Na-2H]^+^ = 781.5627; phosphatidylcholine (34:0) [M+H-Na]^+^ =784.5827; all other metabolites listed were [M+H]^+^.
