# Supplementary material for: Metabolomics-Based Discovery of Small Molecule Biomarkers in Serum Associated with Dengue Virus Infections and Disease Outcomes
Source: PLoS Negl Trop Dis. 2016 Feb 25;10(2):e0004449. doi: 10.1371/journal.pntd.0004449 (PMC4768770; doi:10.1371/journal.pntd.0004449)
Supplement: S2 Table — MSI Level 1: Metabolites identified by HILIC- MS/MS*or MRM-LC-MS/MS* spectra matched with spectra of chemical reference standards acquired on the same analytical platform. MSI Level 2: Metabolites identified by HILIC-MS* and spectrum similarity with public/commercial spectrum libraries. MSI Level 3: Metabolites putatively characterized based on physicochemical characteristics of a chemical class of compounds or by spectrum similarity to known compounds. MSI Level 4: Unidentified or unclassified MFs that can be differentiated or quantified based on spectrum data. Bolded values statistically differentiated the pairwise comparison of the two diagnosis groups. (DOCX) [file pntd.0004449.s012.docx]

**S2 Table. Mexican serum metabolites that differentiate dengue outcomes in at least one of the pairwise comparisons of the DHF/DSS, DF, and ND* diagnosis groups.** MSI Level 1: Metabolites identified by HILIC- MS/MS*or MRM-LC-MS/MS* spectra matched with spectra of chemical reference standards acquired on the same analytical platform. MSI Level 2: Metabolites identified by HILIC-MS* and spectrum similarity with public/commercial spectrum libraries. MSI Level 3: Metabolites putatively characterized based on physicochemical characteristics of a chemical class of compounds or by spectrum similarity to known compounds. MSI Level 4: Unidentified or unclassified MFs that can be differentiated or quantified based in spectrum data. Bolded values statistically differentiated the pairwise comparison of the two diagnosis groups.

|  | | **Mass** | | | **RT*** | | | | **Identity** | | | **ChemicalFormula** | | | **DB identifier*** | | | | **DHF/DSS Vs DF* p-value** | **DHF/DSS Vs DF FC*** | **DHF/DSS Vs NEG* p-value** | | **DHF/DSS Vs NEG* FC** | | | | | **DF Vs NEG* p-value** | **DF Vs NEG FC*** | | |  |
| --- | --- | --- | --- | --- | --- | --- | --- | --- | --- | --- | --- | --- | --- | --- | --- | --- | --- | --- | --- | --- | --- | --- | --- | --- | --- | --- | --- | --- | --- | --- | --- | --- |
|  |  |  |  |  |  |  |  |  |  |  |  |  |  |  |  |  |  |  |  |  |  |  |  |  |  |  |  |  |  |  |  |  |
| **MSI Level 1** | | | | | | | | | | | | | | | | | | | | | | | | | | | | | | | |  |
| ******* | | 278.2245 | | | | 1.1 | | α-Linolenic acid | | | | | | C18H30 O2 | | Metlin 192 | | | >0.05 | <2 | | **5.04E-04** | **-8.61** | | | **3.32E-03** | | | | **-7.27** | |  |
| ******* | | 328.2402 | | | | 1.15 | | Docosahexaenoic acid | | | | | | C22 H32 O2 | | Kegg C06429 | | | **5.58E-04** | **-9.6** | | **4.18E-02** | <2 | | | **4.11E-03** | | | | **2.00** | |  |
| ****** | | 302.2242 | | | | 1.30 | | Arachidonic acid | | | | | | C20H30O2 | | Metlin 35293 | | | **8.09E-03** | **3.75** | | **7.87E-02** | <2 | | | **2.93E-02** | | | | **-2.10** | |  |
|  | | 384.3387 | | | | 1.15 | | Vitamin D3 | | | | | | C27H44O | | Metlin 165 | | | >0.05 | <2 | | **6.53E-03** | **-6.53** | | | **2.27E-02** | | | | **-5.02** | |  |
| ****** | | 400.3339 | | | | 1.16 | | 1α-hydroxyvitamin D3 | | | | | | C27H44O2 | | Metlin 42168 | | | >0.05 | <2 | | **2.16E-03** | **-7.64** | | | **1.25E-02** | | | | **-5.79** | |  |
| ****** | | 416.3275 | | | | 1.31 | | 1,25-dihydroxyvitamin D3 | | | | | | C27H44O3 | | Kegg C01673 | | | **6.27E-02** | **2.66** | | **2.52E-04** | **-8.30** | | | **3.06E-04** | | | | **-8.08** | |  |
| ****** | | 495.3337 | | | | 14.97 | | LysoPC(16:0) | | | | | | C24H50NO7P | | Metlin 40284 | | | >0.05 | <2 | | **2.42E-04** | **11.69** | | | **1.22E-03** | | | | **10.07** | |  |
| ****** | | 521.3493 | | | | 14.8 | | LysoPC(18:1) | | | | | | C26H52NO7P | | HMDB02815 | | | >0.05 | <2 | | **2.47E-04** | **9.60** | | | **1.24E-03** | | | | **8.49** | |  |
| **MSI Level 2** | | | | | | | | | | | | | | | | | | | | | | | | | | | | | | | |  |
| ** | | 226.1932^****^ | | | | 1.33 | | Myristoleic acid | | | | | | C14 H26 O2 | | NIST 1114112 | | | >0.05 | <2 | | **3.27E-03** | **-6.77** | | | **6.47E-03** | | | | **-6.09** | |  |
| ****** | | 759.5778^****^ | | | | 12.3 | | Phosphatidylcholine (34:1) | | | | | | C42 H82 NO8 P | | NIST 112638 | | | **5.47E-02** | **2.56** | | **5.90E-04** | **-13.35** | | | **3.12E-04** | | | | **-13.65** | |  |
| ****** | | 761.5934^****^ | | | | 11.9 | | Phosphatidylcholine (34:0) | | | | | | C42H84NO8P | | NIST 112620 | | | >0.05 | <2 | | **1.01E-02** | **-9.87** | | | **3.65E-03** | | | | **-6.13** | |  |
| ****** | | 771.5415 | | | | 12.2 | | Phosphatidylcholine (36:1) | | | | | | C44H86NO7P | | NIST 1047871 | | | >0.05 | <2 | | >0.05 | <2 | | | >0.05 | | | | **2.18** | |  |
| **MSI Level 3** | | | | | | | | | | | | | | | | | | | | | | | | | | | | | | | |  |
|  | | | **Mass** | **RT*** | | | **Potential ID** | | | | | | **Calculated Formula** | | | | **# DB hits*** | **DB identifier*** | **DHF/DSS Vs DF* p-value** | **DHF/DSS Vs DF FC*** | | **DHF/DSS Vs NEG* p-value** | | | **DHF/DSS Vs NEG FC *** | | | **DF Vs NEG* p-value** | | | **DF Vs NEG FC*** |  |
|  |  |  |  |  |  |  |  |  |  |  |  |  |  |  |  |  |  |  |  |  |  |  |  |  |  |  |  |  |  |  |  |  |
|  | | | 109.0017 | 30.69 | | | Hypotaurine | | | | | | C2H7NO2S | | | | 1 | HMDB00965 | >0.05 | **-2.54** | | **4.63E-04** | | | **-8.56** | | **8.98E-04** | | | **-7.99** | |  |
|  | | | 116.0839 | 2.14 | | | Caproic acid | | | | | | C6H12O2 | | | | >5 | HMDB00535 | >0.05 | <2 | | **2.44E-02** | | | **-5.39** | | **3.30E-03** | | | **-6.50** | |  |
|  | | | 117.079 | 17.91 | | | Valine | | | | | | C5H11NO2 | | | | >5 | Kegg C00183 | **5.29E-02** | **-3.94** | | **2.36E-04** | | | **-12.37** | | **6.31E-06** | | | **-14.01** | |  |
|  | | | 149.0508 | 9.26 | | | Methionine | | | | | | C5H11NO2S | | | | 3 | Kegg C00073 | **6.87E-02** | **-3.27** | | **1.29E-02** | | | **-6.01** | | **2.26E-02** | | | **-5.38** | |  |
|  | | | 210.1617 | 1.16 | | | Tridecadienoic acid | | | | | | C13H22O2 | | | | >5 | Metlin 34909 | >0.05 | <2 | | **5.62E-04** | | | **-6.62** | | **2.62E-02** | | | **-4.65** | |  |
|  | | | 241.2405 | 1.44 | | | (4E,6E,d14:2) sphingosine | | | | | | C14H27NO2 | | | | >5 | Metlin 53907 | >0.05 | <2 | | **4.55E-03** | | | **3.65** | | **7.65E-02** | | | **2.45** | |  |
|  | | | 243.1833 | 1.23 | | | N-Undecanoylglycine | | | | | | C13H25NO3 | | | | >5 | HMDB13286 | >0.05 | <2 | | **1.01E-03** | | | **-8.46** | | **5.55E-03** | | | **-7.15** | |  |
| ****** | | | 294.2195 | 1.13 | | | 13-hydroxyoctadeca-9,11,15-trienoic acid | | | | | | C18H30O3 | | | | >5 | Metlin 36023 | >0.05 | <2 | | **9.12E-03** | | | **-5.04** | | **7.92E-03** | | | **-4.71** | |  |
|  | | | 296.1833 | 5.45 | | | Eicosatetraynoic acid | | | | | | C20H24O2 | | | | 1 | Metlin 62956 | >0.05 | <2 | | **3.39E-04** | | | **-8.12** | | **2.24E-03** | | | **-7.03** | |  |
|  | | | 299.2822 | 1.45 | | | Sphingosine | | | | | | C18H37NO2 | | | | >5 | Kegg C00319 | >0.05 | **2.49** | | **1.44E-03** | | | **-8.03** | | **6.16E-03** | | | **-6.66** | |  |
| ******* | | | 300.209 | 1.17 | | | Retinoic acid | | | | | | C20H28O2 | | | | >5 | Metlin 2277 | >0.05 | <2 | | >0.05 | | | **-2.84** | | **6.93E-02** | | | **-3.62** | |  |
| ****** | | | 318.22 | 1.12 | | | Leukotriene A4 | | | | | | C20H30O3 | | | | >5 | Kegg C00909 | >0.05 | <2 | | **2.52E-03** | | | **-8.02** | | **2.77E-04** | | | **-8.81** | |  |
| ******* | | | 324.2145 | 3.95 | | | 4,7,10,13-Docosatetraynoic acid | | | | | | C22H28O2 | | | | >5 | Metlin 74281 | >0.05 | **2.02** | | **1.89E-03** | | | **-8.14** | | **5.64E-03** | | | **-7.23** | |  |
| ****** | | | 326.224 | 1.12 | | | Ala Ala Ala Asp | | | | | | C22H30O2 | | | | 5 | Metlin 62961 | >0.05 | <2 | | **2.91E-02** | | | **-5.83** | | **4.91E-05** | | | **-9.20** | |  |
|  | | | 346.1965 | 4 | | | Thr Arg Ala | | | | | | C13H26N6O5 | | | | >5 | Metlin 17763 | >0.05 | <2 | | **4.07E-04** | | | **-8.53** | | **4.97E-04** | | | **-8.09** | |  |
|  | | | 350.2092 | 1.32 | | | Prostaglandin D3 | | | | | | C20H30O5 | | | | >5 | Metlin 34497 | **7.06E-03** | **6.82** | | **7.21E-05** | | | **10.59** | | **2.39E-11** | | | **8.56** | |  |
|  | | | 356.2708 | 1.36 | | | Docosahexaenoic acid ethyl ester | | | | | | C24H36O2 | | | | >5 | Kegg C16185 | >0.05 | **-3.17** | | **7.55E-03** | | | **-6.64** | | **3.81E-04** | | | **-7.69** | |  |
|  | | | 370.2348 | 1.21 | | | 6-Keto-prostaglandin F1alpha | | | | | | C20H34O6 | | | | >5 | Kegg C05961 | >0.05 | **-2.11** | | **4.41E-05** | | | **-7.85** | | **1.03E-05** | | | **-7.85** | |  |
|  | | | 380.2203 | 1.21 | | | Pro His Lys | | | | | | C17H28N6O4 | | | | >5 | Metlin 17284 | >0.05 | <2 | | **1.36E-02** | | | **-5.93** | | **2.03E-04** | | | **-7.48** | |  |
|  | | | 382.1087 | 4.38 | | | Thr Gly Cys Cys | | | | | | C12H22N4O6S2 | | | | >5 | Metlin 233500 | >0.05 | <2 | | **3.01E-04** | | | **-10.08** | | **1.96E-04** | | | **-10.08** | |  |
|  | | | 384.3387 | 1.15 | | | Vitamin D3 | | | | | | C27H44O | | | | >5 | Metlin 165 | >0.05 | <2 | | **6.53E-03** | | | **-6.53** | | **2.27E-02** | | | **-5.02** | |  |
|  | | | 399.2827 | 4.16 | | | Arg Pro Lys | | | | | | C17H33N7O4 | | | | >5 | Metlin 16057 | >0.05 | **-2.11** | | **8.36E-05** | | | **-9.70** | | **7.36E-05** | | | **-9.36** | |  |
| ******* | | | 429.2933 | 5.87 | | | Arg Arg Val | | | | | | C20H39N5O5 | | | | >5 | Metlin 15788 | >0.05 | **-2.09** | | **5.20E-04** | | | **-8.48** | | **1.89E-03** | | | **-7.50** | |  |
|  | | | 453.33 | 1.51 | | | PC(O-12:0/O-2:0) | | | | | | C22H48NO6P | | | | >5 | Metlin 40190 | >0.05 | **-2.32** | | **9.58E-03** | | | **-6.19** | | **6.58E-03** | | | **-6.18** | |  |
|  | | | 457.3249 | 4.35 | | | N-docosahexaenoyl glutamic acid | | | | | | C22H43N5O5 | | | | >5 | LMFA08020089 | >0.05 | **-2.4** | | **2.08E-03** | | | **-7.87** | | **2.67E-04** | | | **-8.40** | |  |
| ****** | | | 467.3011 | 14.89 | | | PC(14:0) | | | | | | C22H46NO7P | | | | >5 | Metlin 40278 | >0.05 | <2 | | **8.62E-04** | | | **-9.03** | | **1.23E-02** | | | **-6.63** | |  |
|  | | | 469.3248 | 1.33 | | | Leu Pro Ile Lys | | | | | | C23H43N5O5 | | | | >5 | Metlin 180427 | >0.05 | **-2.33** | | **4.75E-04** | | | **-7.33** | | **1.37E-04** | | | **-7.43** | |  |
|  | | | 473.2775 | 1.19 | | | Leu Trp Arg | | | | | | C23H35N7O4 | | | | >5 | Metlin 15833 | >0.05 | **2.43** | | **1.85E-04** | | | **-9.17** | | **7.17E-02** | | | **-4.89** | |  |
| ******* | | | 473.3195 | 6.79 | | | Docosa-4,7,10,13,16-pentaenoyl carnitine | | | | | | C22H43N5O6 | | | | >5 | HMDB06321 | >0.05 | <2 | | **1.10E-04** | | | **-9.24** | | **6.57E-04** | | | **-8.17** | |  |
|  | | | 479.3375 | 14.11 | | | PC(O-16:1) | | | | | | C24H50NO6P | | | | >5 | LMGP01060028 | **1.31E-02** | **5.94** | | **2.42E-04** | | | **9.29** | | **6.99E-02** | | | **3.36** | |  |
| ****** | | | 481.3166 | 14.72 | | | PC(15:0) | | | | | | C23H48NO7P | | | | >5 | LMGP01050016 | >0.05 | **-2.33** | | **2.32E-03** | | | **-7.94** | | **2.73E-02** | | | **-5.61** | |  |
|  | | | 492.378 | 1.09 | | | PC(16:1) | | | | | | C30H52O5 | | | | 2 | LMGP01050021 | >0.05 | **2.17** | | **6.96E-04** | | | **-7.46** | | **5.36E-03** | | | **-6.21** | |  |
|  | | | 493.3165 | 14.68 | | | PC(16:1) | | | | | | C24H48NO7P | | | | 5 | Metlin 40287 | >0.05 | <2 | | **3.16E-03** | | | **-8.41** | | **1.02E-02** | | | **-7.25** | |  |
|  | | | 501.3672 | 1.67 | | | Lys Lys Lys Val | | | | | | C23H47N7O5 | | | | 4 | Metlin 170856 | >0.05 | **-2.27** | | **6.39E-04** | | | **10.14** | | **6.35E-03** | | | **7.71** | |  |
|  | | | 507.3684 | 14.8 | | | PC(O-18:1) | | | | | | C26H54NO6P | | | | >5 | LMGP01060034 | >0.05 | **3.84** | | **2.17E-03** | | | **-8.35** | | **1.34E-02** | | | **-6.61** | |  |
|  | | | 509.3487 | 14.41 | | | LysoPE(20:0) | | | | | | C25H52NO7P | | | | >5 | HMDB11481 | >0.05 | **3.71** | | **2.44E-04** | | | **9.62** | | **8.92E-03** | | | **5.91** | |  |
|  | | | 519.333 | 14.48 | | | LysoPC(18:2) | | | | | | C26H50NO7P | | | | 5 | HMDB10386 | >0.05 | <2 | | **2.44E-04** | | | **10.67** | | **5.71E-04** | | | **10.2** | |  |
|  | | | 523.365 | 14.72 | | | PC(O-18:0) | | | | | | C26H54NO7P | | | | >5 | Metlin 40075 | >0.05 | <2 | | **2.44E-04** | | | **10.67** | | **5.71E-04** | | | **10.20** | |  |
|  | | | 543.3324 | 14.29 | | | PC(20:4) | | | | | | C28H50NO7P | | | | >5 | LMGP01050048 | >0.05 | <2 | | **4.93E-03** | | | **-9.12** | | **1.59E-02** | | | **-7.50** | |  |
|  | | | 549.3784 | 14.13 | | | PC(O-20:1) | | | | | | C28H56NO7P | | | | >5 | LMGP01020150 | >0.05 | <2 | | **4.78E-03** | | | **-7.37** | | **2.53E-02** | | | **-5.73** | |  |
|  | | | 562.4604 | 0.98 | | | DG(32:3) | | | | | | C35H62O5 | | | | >5 | Metlin 58652 | >0.05 | <2 | | **6.49E-04** | | | **-6.04** | | **3.17E-02** | | | **-4.18** | |  |
|  | | | 565.3149 | 14.68 | | | Lys Lys Gln Tyr | | | | | | C26H43N7O7 | | | | >5 | Metlin 170958 | >0.05 | <2 | | **2.44E-04** | | | **9.62** | | **8.92E-03** | | | **5.91** | |  |
|  | | | 572.377 | 6.97 | | | Vitamin D2 3-glucuronide | | | | | | C34H52O7 | | | | 4 | Metlin 57895 | >0.05 | <2 | | **4.81E-04** | | | **-7.83** | | **2.31E-04** | | | **-7.84** | |  |
| ****** | | | 579.3556 | 13.92 | | | PS(22:1) | | | | | | C28H54NO9P | | | | 1 | LMGP03050023 | >0.05 | <2 | | **8.86E-05** | | | **-8.60** | | **8.54E-06** | | | **-8.90** | |  |
|  | | | 585.3034 | 14.97 | | | Pro Gln Arg Trp | | | | | | C27H39N9O6 | | | | >5 | Metlin 204977 | >0.05 | <2 | | **2.41E-04** | | | **10.43** | | **8.69E-03** | | | **6.59** | |  |
|  | | | 589.4033 | 6.97 | | | PC(22:2) | | | | | | C30H56NO8P | | | | >5 | Metlin 39167 | >0.05 | <2 | | **4.14E-02** | | | **5.11** | | >0.05 | | | **1.63** | |  |
|  | | | 593.3697 | 13.82 | | | PC(16:0/5:0(CHO)) | | | | | | C29H56NO9P | | | | >5 | LMGP20010005 | >0.05 | <2 | | **1.56E-04** | | | **-9.35** | | **1.29E-03** | | | **-7.79** | |  |
|  | | | 611.3182 | 14.8 | | | Phe Phe Ile Trp | | | | | | C35H41N5O5 | | | | >5 | Metlin 137238 | >0.05 | <2 | | **2.46E-04** | | | **9.17** | | **8.82E-03** | | | **5.85** | |  |
|  | | | 617.4489 | 1.94 | | | CerP(d34:1) | | | | | | C34H68NO6P | | | | >5 | LMSP02050002 | >0.05 | **-2.36** | | **2.49E-03** | | | **-6.87** | | **1.84E-03** | | | **-6.75** | |  |
|  | | | 630.4846 | 0.97 | | | PA(P-32:1) | | | | | | C35H67O7P | | | | >5 | LMGP10030008 | >0.05 | <2 | | **2.42E-03** | | | **-6.39** | | **4.78E-04** | | | **-6.68** | |  |
|  | | | 633.4457 | 2.1 | | | PE(28:1) | | | | | | C33H64NO8P | | | | >5 | Metlin 60267 | >0.05 | <2 | | **7.01E-03** | | | **7.67** | | **4.35E-02** | | | **5.42** | |  |
| ****** | | | 635.416 | 13.24 | | | PE(28:0) | | | | | | C33H66NO8P | | | | >5 | Metlin 40418 | >0.05 | **3.33** | | **2.46E-04** | | | **9.17** | | **8.82E-03** | | | **5.85** | |  |
|  | | | 640.4757 | 1.72 | | | DG(38:6) | | | | | | C41H68O5 | | | | >5 | LMGL02010130 | >0.05 | <2 | | **4.99E-03** | | | **-6.53** | | **6.82E-03** | | | **-6.05** | |  |
|  | | | 645.4661 | 3.36 | | | PE(P-30:1) | | | | | | C35H68NO7P | | | | >5 | LMGP02030010 | >0.05 | **-2.45** | | **1.37E-06** | | | **-10.05** | | **3.34E-09** | | | **-10.72** | |  |
|  | | | 647.445 | 7.03 | | | PC(26:1) | | | | | | C34H66NO8P | | | | 3 | LMGP01011316 | >0.05 | <2 | | **5.51E-04** | | | **-7.93** | | **9.19E-06** | | | **-8.72** | |  |
| ******* | | | 647.6207 | 1.3 | | | Nervonic ceramide | | | | | | C42H81NO3 | | | | >5 | Metlin 63014 | >0.05 | <2 | | **2.35E-03** | | | **-7.10** | | **1.99E-02** | | | **-5.28** | |  |
|  | | | 654.4913 | 1.57 | | | PA(O-34:4) | | | | | | C37H67O7P | | | | >5 | LMGP10020011 | >0.05 | <2 | | **5.02E-03** | | | **-5.75** | | **3.61E-02** | | | **-4.29** | |  |
|  | | | 656.4712 | 2.41 | | | PA(O-34:3) | | | | | | C37H69O7P | | | | >5 | LMGP10020079 | >0.05 | **-2.14** | | **4.53E-03** | | | **-8.12** | | **2.01E-02** | | | **-6.46** | |  |
|  | | | 663.4458 | 12.99 | | | PS(P-28:0) | | | | | | C34H66NO9P | | | | >5 | LMGP03030001 | >0.05 | <2 | | **5.98E-06** | | | **-8.66** | | **1.04E-06** | | | **-8.66** | |  |
|  | | | 671.4823 | 1.76 | | | PE(32:2) | | | | | | C37H70NO7P | | | | 4 | Metlin 60329 | >0.05 | <2 | | **7.50E-04** | | | **-7.17** | | **1.76E-05** | | | **-7.96** | |  |
| ****** | | | 672.5215 | 13.83 | | | SM(d32:2) | | | | | | C37H73N2O6P | | | | 4 | LMSP03010034 | >0.05 | <2 | | **2.52E-03** | | | **-7.87** | | **3.53E-02** | | | **-5.42** | |  |
|  | | | 675.4761 | 4.83 | | | PC(28:1) | | | | | | C36H70NO8P | | | | >5 | LMGP01010392 | >0.05 | <2 | | **6.06E-04** | | | **-7.80** | | **9.46E-05** | | | **-8.03** | |  |
|  | | | 684.5019 | 1.93 | | | PA(O-36:3) | | | | | | C39H73O7P | | | | >5 | LMGP10020030 | >0.05 | <2 | | **2.27E-03** | | | **-7.61** | | **2.71E-02** | | | **-5.47** | |  |
|  | | | 687.5124 | 2.15 | | | PC(P-30:1) | | | | | | C38H74NO7P | | | | >5 | LMGP01030022 | >0.05 | <2 | | **4.66E-03** | | | **-8.39** | | **4.03E-03** | | | **-8.12** | |  |
|  | | | 699.4473 | 12.89 | | | PS(30:4) | | | | | | C36H62NO10P | | | | >5 | LMGP03010056 | >0.05 | **-2.25** | | **1.60E-06** | | | **-10.57** | | **3.64E-09** | | | **-11.28** | |  |
|  | | | 700.4971 | 2.93 | | | PA(36:2) | | | | | | C39H73O8P | | | | >5 | LMGP10010036 | >0.05 | <2 | | **3.94E-02** | | | **5.41** | | **9.21E-02** | | | **3.97** | |  |
|  | | | 702.5636 | 13.88 | | | SM(d34:1) | | | | | | C40H79O7P | | | | >5 | LMSP03010003 | >0.05 | <2 | | **4.11E-02** | | | **-6.22** | | **1.34E-02** | | | **-6.97** | |  |
|  | | | 703.5077 | 3.46 | | | PC(30:1) | | | | | | C38H74NO8P | | | | >5 | LMGP01010440 | **7.81E-02** | **-3.56** | | **1.00E-02** | | | **-6.94** | | **2.16E-02** | | | **-6.07** | |  |
|  | | | 715.5414 | 1.71 | | | PC(O-32:2) | | | | | | C40H78NO7P | | | | >5 | LMGP01020017 | >0.05 | <2 | | **1.05E-02** | | | **-5.30** | | **6.89E-04** | | | **-6.16** | |  |
|  | | | 718.5531 | 1.09 | | | DG(44:9) | | | | | | C40H79O8P | | | | >5 | LMGL02010300 | >0.05 | <2 | | **4.95E-06** | | | **-10.99** | | **5.06E-05** | | | **-9.69** | |  |
|  | | | 719.5017 | 6.38 | | | PS(O-32:1) | | | | | | C38H74NO9P | | | | >5 | LMGP03020007 | >0.05 | <2 | | **5.69E-04** | | | **-7.43** | | **1.04E-04** | | | **-7.61** | |  |
|  | | | 728.5825 | 13.77 | | | SM(d36:2) | | | | | | C41H81N2O6P | | | | >5 | LMSP03010029 | >0.05 | <2 | | **9.25E-03** | | | **-8.04** | | **1.33E-02** | | | **-7.47** | |  |
|  | | | 729.5305 | 12.15 | | | PC(32:2) | | | | | | C40H76NO8P | | | | >5 | LMGP01010494 | >0.05 | **-2.94** | | >0.05 | | | **2.67** | | **6.49E-03** | | | **5.61** | |  |
|  | | | 731.5462 | 12.24 | | | PC(32:1) | | | | | | C40H78NO8P | | | | >5 | Metlin 40731 | >0.05 | <2 | | **5.20E-03** | | | **-9.36** | | **1.11E-03** | | | **-10.33** | |  |
|  | | | 745.5549 | 2.2 | | | PE(36:1) | | | | | | C41H80NO8P | | | | >5 | Metlin 40425 | >0.05 | <2 | | **1.81E-02** | | | **-6.75** | | **1.02E-02** | | | **-6.93** | |  |
|  | | | 747.5343 | 4.27 | | | PS(O-34:1) | | | | | | C43H74NO7P | | | | >5 | LMGP03020044 | >0.05 | <2 | | **2.26E-03** | | | **-8.10** | | **1.78E-03** | | | **-7.87** | |  |
| ******* | | | 753.5306 | 11.89 | | | PC(34:4) | | | | | | C42H76NO8P | | | | >5 | LMGP01010506 | >0.05 | <2 | | **1.10E-02** | | | **-6.81** | | **4.00E-02** | | | **-5.46** | |  |
| ****** | | | 755.5477 | 12.98 | | | PC(34:3) | | | | | | C42H78NO8P | | | | >5 | HMDB07881 | 8.73E-02 | **4.79** | | **2.52E-04** | | | **10.51** | | **1.61E-02** | | | **5.72** | |  |
|  | | | 757.5633 | 12.14 | | | PC(34:2) | | | | | | C42H80NO8P | | | | >5 | Metlin 39327 | >0.05 | <2 | | **5.89E-04** | | | **-13.91** | | **4.40E-03** | | | **-11.32** | |  |
|  | | | 759.5764 | 12.26 | | | PC(34:1) | | | | | | C42H82NO8P | | | | >5 | LMGP01010005 | >0.05 | <2 | | **9.30E-02** | | | **-4.78** | | **8.18E-03** | | | **-6.80** | |  |
| ****** | | | 763.5513 | 11.73 | | | PC(P-36:5) | | | | | | C40H78NO10P | | | | >5 | LMGP01030040 | >0.05 | <2 | | **6.81E-05** | | | **-9.63** | | **1.53E-02** | | | **-6.06** | |  |
|  | | | 765.5672 | 11.79 | | | PC(O-36:5) | | | | | | C44H80NO7P | | | | >5 | LMGP01020058 | >0.05 | **2.54** | | **6.67E-03** | | | **-8.47** | | **4.01E-03** | | | **-8.92** | |  |
|  | | | 769.5966 | 12.37 | | | PC(36:2) | | | | | | C44H84NO7P | | | | >5 | Metlin 59542 | >0.05 | <2 | | **3.78E-03** | | | **-7.04** | | **3.99E-02** | | | **-5.27** | |  |
| ****** | | | 773.5581 | 12.28 | | | PS(O-36:2) | | | | | | C42H80NO9P | | | | 5 | LMGP03020013 | >0.05 | <2 | | **1.28E-04** | | | **-12.73** | | **2.52E-03** | | | **-9.86** | |  |
|  | | | 775.5647 | 2.95 | | | PS(O-36:1) | | | | | | C45H78NO7P | | | | >5 | LMGP03020012 | >0.05 | <2 | | **2.81E-03** | | | **-8.56** | | **7.53E-03** | | | **-7.42** | |  |
| ******* | | | 777.5327 | 11.78 | | | PC(36:6) | | | | | | C44H76NO8P | | | | >5 | HMDB07892 | **1.58E-02** | **-3.32** | | **3.99E-03** | | | **-7.28** | | **7.53E-03** | | | **-6.67** | |  |
| ******* | | | 779.5471 | 11.84 | | | PC(36:5) | | | | | | C44H78NO8P | | | | >5 | HMDB07890 | >0.05 | <2 | | **2.07E-04** | | | **-10.22** | | **6.97E-04** | | | **-9.43** | |  |
| ****** | | | 783.5769 | 11.99 | | | PC(36:3) | | | | | | C44H82NO8P | | | | >5 | LMGP01010622 | >0.05 | <2 | | **1.60E-03** | | | **-8.11** | | **6.00E-03** | | | **-7.08** | |  |
|  | | | 793.5983 | 11.92 | | | PC(O-38:5) | | | | | | C46H84NO7P | | | | >5 | LMGP01020066 | >0.05 | <2 | | **9.26E-04** | | | **-10.90** | | **2.04E-03** | | | **-10.08** | |  |
|  | | | 795.5417 | 11.51 | | | PS(O-38:5) | | | | | | C44H78NO9P | | | | 4 | LMGP03020085 | **3.83E-02** | **-3.3** | | **1.44E-05** | | | **-10.48** | | **4.71E-06** | | | **-10.33** | |  |
|  | | | 797.5585 | 11.95 | | | PS(O-38:4) | | | | | | C44H80NO9P | | | | >5 | LMGP03020017 | >0.05 | <2 | | **2.17E-03** | | | **-10.41** | | **2.95E-03** | | | **-9.80** | |  |
| ******* | | | 799.5724 | 12.09 | | | PS(O-38:3) | | | | | | C44H82NO9P | | | | >5 | LMGP03020036 | >0.05 | <2 | | **1.59E-04** | | | **-11.55** | | **4.93E-04** | | | **-10.57** | |  |
|  | | | 801.587 | 12.18 | | | PS(O-38:2) | | | | | | C44H84NO9P | | | | >5 | LMGP03020016 | **8.59E-02** | **-3.65** | | **1.19E-02** | | | **-7.94** | | **3.03E-02** | | | **-6.68** | |  |
|  | | | 803.546 | 11.77 | | | PC(38:7) | | | | | | C46H78NO8P | | | | >5 | LMGP01010696 | >0.05 | <2 | | **2.06E-03** | | | **-9.05** | | **3.75E-03** | | | **-8.23** | |  |
| ****** | | | 805.5636 | 12.46 | | | PC(38:6) | | | | | | C46H80NO8P | | | | >5 | Metlin 39390 | >0.05 | **2.54** | | **2.40E-04** | | | **11.26** | | **2.50E-03** | | | **8.72** | |  |
| ****** | | | 807.5771 | 11.8 | | | PC(38:5) | | | | | | C46H82NO8P | | | | >5 | LMGP01010645 | >0.05 | <2 | | **2.27E-02** | | | **-8.72** | | **1.54E-02** | | | **-9.04** | |  |
|  | | | 809.5941 | 11.9 | | | PC(38:4) | | | | | | C46H84NO8P | | | | >5 | Metlin 39383 | >0.05 | <2 | | **2.27E-02** | | | **-8.72** | | **1.54E-02** | | | **-9.04** | |  |
| ****** | | | 813.5543 | 12.27 | | | PS(38:3) | | | | | | C44H80NO10P | | | | >5 | HMDB12382 | >0.05 | <2 | | **2.00E-02** | | | **-6.94** | | **4.05E-05** | | | **-10.36** | |  |
| ****** | | | 815.569 | 11.68 | | | PS(38:2) | | | | | | C44H82NO10P | | | | >5 | LMGP03010200 | >0.05 | <2 | | **3.67E-03** | | | **-8.02** | | **1.27E-05** | | | **-9.89** | |  |
| ****** | | | 819.5461 | 12.31 | | | PS(P-40:6) | | | | | | C46H78NO9P | | | | 1 | LMGP03030089 | >0.05 | **2.57** | | **6.78E-05** | | | **-8.80** | | **2.63E-02** | | | **-5.49** | |  |
| ****** | | | 825.5889 | 12.51 | | | PS(O-40:4) | | | | | | C46H84NO9P | | | | 4 | LMGP03020040 | >0.05 | **2.88** | | **9.83E-04** | | | **8.79** | | **1.50E-02** | | | **5.91** | |  |
|  | | | 827.5983 | 11.96 | | | PS(O-40:3) | | | | | | C46H86NO9P | | | | 3 | LMGP03020062 | >0.05 | <2 | | **1.37E-03** | | | **-8.74** | | **8.08E-02** | | | **-5.09** | |  |
|  | | | 829.5567 | 11.57 | | | PC(40:8) | | | | | | C48H80NO8P | | | | >5 | LMGP01010947 | >0.05 | <2 | | **5.60E-03** | | | **-8.26** | | **1.09E-02** | | | **-7.49** | |  |
|  | | | 835.6061 | 11.85 | | | PC(40:5) | | | | | | C48H86NO8P | | | | >5 | LMGP01010816 | >0.05 | <2 | | **5.40E-03** | | | **-8.92** | | **4.19E-03** | | | **-8.88** | |  |
| ****** | | | 837.5527 | 12.25 | | | PS(40:5) | | | | | | C46H80NO10P | | | | >5 | HMDB10166 | >0.05 | <2 | | **9.10E-08** | | | **-13.54** | | **2.67E-08** | | | **-13.24** | |  |
|  | | | 837.6172 | 11.98 | | | PC(40:4) | | | | | | C48H88NO8P | | | | >5 | LMGP01010813 | >0.05 | <2 | | **4.83E-04** | | | **-9.24** | | **3.22E-06** | | | **-10.86** | |  |
| ****** | | | 841.5825 | 11.48 | | | PS(40:3) | | | | | | C46H84NO10P | | | | >5 | LMGP03010338 | >0.05 | <2 | | **2.87E-03** | | | **-8.71** | | **5.17E-03** | | | **-7.77** | |  |
|  | | | 847.5713 | 11.74 | | | PS(P-42:6) | | | | | | C48H82NO9P | | | | 1 | LMGP03030093 | >0.05 | <2 | | **1.75E-02** | | | **-6.41** | | **3.04E-02** | | | **-5.67** | |  |
|  | | | 849.6019 | 5.23 | | | PS(O-42:6) | | | | | | C48H84NO9P | | | | >5 | LMGP03020093 | >0.05 | **3.54** | | **6.97E-04** | | | **-8.69** | | **4.81E-04** | | | **-8.44** | |  |
| ****** | | | 863.5646 | 11.42 | | | PS(42:6) | | | | | | C48H82NO10P | | | | >5 | LMGP03010587 | >0.05 | <2 | | **6.47E-03** | | | **-7.60** | | **4.77E-03** | | | **-7.47** | |  |
|  | | | 867.5954 | 11.49 | | | PS(42:4) | | | | | | C48H86NO10P | | | | >5 | LMGP03010527 | >0.05 | <2 | | **7.80E-05** | | | **-8.29** | | **1.78E-05** | | | **-8.37** | |  |
| ****** | | | 887.5615 | 11.38 | | | PS(44:8) | | | | | | C50H82NO10P | | | | 3 | LMGP03010786 | >0.05 | <2 | | **2.50E-03** | | | **-6.73** | | **9.32E-06** | | | **-8.07** | |  |
|  | | | 893.6284 | 6.04 | | | PS(44:5) | | | | | | C50H88NO10P | | | | >5 | LMGP03010754 | >0.05 | **-2.06** | | **1.78E-04** | | | **-9.02** | | **1.87E-05** | | | **-9.28** | |  |
|  | | | 924.6716 | 5.36 | | | TG(58:11) | | | | | | C61H96O6 | | | | >5 | LMGL03011393 | >0.05 | **-2.17** | | **7.25E-04** | | | **-7.24** | | **5.94E-04** | | | **-6.94** | |  |
|  | | | 937.6546 | 6.77 | | | FMC-5(d36:1) | | | | | | C52H91NO13 | | | | 1 | LMSP05010032 | >0.05 | <2 | | **9.06E-04** | | | **-7.61** | | **3.60E-03** | | | **-6.65** | |  |
|  | | | 948.6596 | 5.43 | | | PI(42:1) | | | | | | C51H97O13P | | | | 4 | LMGP06010493 | >0.05 | <2 | | **1.27E-04** | | | **-9.60** | | **1.69E-06** | | | **-10.49** | |  |
|  | | | 965.6865 | 5.43 | | | FMC-5(d38:1) | | | | | | C54H95NO13 | | | | 1 | LMSP05010033 | >0.05 | <2 | | **5.66E-04** | | | **-8.70** | | **3.98E-04** | | | **-8.42** | |  |
|  | | | 976.6492 | 14.67 | | | PI(44:1) | | | | | | C53H101O13P | | | | >5 | LMGP06010691 | >0.05 | <2 | | **7.26E-04** | | | **-7.55** | | **6.12E-02** | | | **-4.47** | |  |
|  | | | 978.6706 | 6.84 | | | PI(44:0) | | | | | | C53H103O13P | | | | >5 | LMGP06010915 | >0.05 | <2 | | **4.80E-05** | | | **-8.94** | | **1.11E-03** | | | **-7.60** | |  |
|  | | | 991.3318 | 14.54 | | | 3-Oxotetradecanoyl-CoA | | | | | | C35H60N7O18P3S | | | | >5 | HMDB03935 | >0.05 | <2 | | **4.92E-04** | | | **-7.49** | | **1.55E-02** | | | **-5.68** | |  |
| **MSI Level 4** | | | | | | | | | | | | | | | | | | | | | | | | | | | | | | | | |
|  | | | **Mass** | | | **RT*** | | | | | **Potential ID** | | | **Calculated Formula** | | | | | | **DHF/DSS Vs DF* p-value** | **DHF/DSS Vs DF FC *** | | **DHF/DSS Vs NEG* p-value** | | **DHF/DSS Vs NEG FC*** | | **DF Vs NEG* p-value** | | | | **DF Vs NEG FC *** | |
| ******* | 110.1098 | | | | | 1.87 | | | | | Unidentified | | C8H14 | | | | | | >0.05 | **-2.58** | | **2.20E-03** | | **-6.94** | | **7.13E-02** | | | | **-4.36** | |  |
|  | 118.0784 | | | | | 1.17 | | | | | Unidentified | | C9H10 | | | | | | >0.05 | <2 | | **3.86E-02** | | **4.82** | | **2.06E-03** | | | | **5.95** | |  |
|  | 125.084 | | | | | 2.21 | | | | | Unidentified | | C7H11NO | | | | | | >0.05 | <2 | | **5.34E-03** | | **-6.63** | | **1.17E-02** | | | | **-5.88** | |  |
|  | 126.0317 | | | | | 4.34 | | | | | Unidentified | | C6H6O3 | | | | | | >0.05 | <2 | | **9.61E-03** | | **-6.27** | | **2.53E-02** | | | | **-5.39** | |  |
|  | 133.0525 | | | | | 0.83 | | | | | Unidentified | | C8H7NO | | | | | | >0.05 | <2 | | **6.04E-04** | | **-7.76** | | **7.15E-02** | | | | **-4.49** | |  |
|  | 138.1042 | | | | | 1.12 | | | | | Unidentified | | C9H14O | | | | | | >0.05 | <2 | | **1.19E-02** | | **-5.53** | | **3.85E-03** | | | | **-5.86** | |  |
|  | 139.0995 | | | | | 1.91 | | | | | Unidentified | | C8H13NO | | | | | | >0.05 | **-3.28** | | **9.27E-04** | | **-7.19** | | **1.36E-02** | | | | **-5.62** | |  |
|  | 140.9512 | | | | | 30.64 | | | | | Unidentified | | C5HO5 | | | | | | **4.68E-02** | **3.92** | | **4.12E-03** | | **-7.50** | | **6.50E-03** | | | | **-7.00** | |  |
|  | 143.0945 | | | | | 18.79 | | | | | Unidentified | | C7H13NO2 | | | | | | >0.05 | **-2.57** | | **9.05E-03** | | **-7.92** | | **1.60E-03** | | | | **-9.14** | |  |
|  | 144.0422 | | | | | 4.36 | | | | | Unidentified | | C6H8O4 | | | | | | >0.05 | <2 | | **3.08E-03** | | **-7.91** | | **1.37E-02** | | | | **-6.55** | |  |
|  | 150.089 | | | | | 3.29 | | | | | Unidentified | | C6H14O4 | | | | | | >0.05 | <2 | | **4.87E-03** | | **-8.43** | | **1.94E-02** | | | | **-6.57** | |  |
|  | 152.0583 | | | | | 4.29 | | | | | Unidentified | | C7H8N2O2 | | | | | | >0.05 | <2 | | **5.64E-04** | | **-9.46** | | **2.41E-03** | | | | **-8.17** | |  |
|  | 154.1366 | | | | | 1.27 | | | | | Unidentified | | C10H18O | | | | | | >0.05 | <2 | | **5.69E-04** | | **-6.37** | | **1.08E-03** | | | | **-5.94** | |  |
|  | 155.0346 | | | | | 17.79 | | | | | Unidentified | | C3H10NO4P | | | | | | >0.05 | <2 | | **4.23E-03** | | **-7.18** | | **1.43E-04** | | | | **-8.18** | |  |
|  | 156.1151 | | | | | 1.19 | | | | | Unidentified | | C9H16O2 | | | | | | **3.24E-02** | **-4.56** | | **1.38E-04** | | **-7.93** | | **1.21E-03** | | | | **-6.90** | |  |
|  | 162.0527 | | | | | 4.38 | | | | | Unidentified | | C6H10O5 | | | | | | >0.05 | <2 | | **1.22E-02** | | **-6.25** | | **2.79E-02** | | | | **-5.42** | |  |
|  | 162.1259 | | | | | 1.51 | | | | | Unidentified | | C8H18O3 | | | | | | >0.05 | <2 | | **1.59E-05** | | **-10.07** | | **1.02E-02** | | | | **-6.13** | |  |
| ****** | 164.1051 | | | | | 2.47 | | | | | Unidentified | | C8H12N4 | | | | | | >0.05 | <2 | | **1.82E-03** | | **-6.49** | | **1.31E-02** | | | | **-5.40** | |  |
| ******* | 166.0992 | | | | | 1.35 | | | | | Unidentified | | C10H14O2 | | | | | | >0.05 | <2 | | **9.38E-03** | | **-6.71** | | **3.73E-02** | | | | **-5.23** | |  |
| ******* | 168.0785 | | | | | 1.32 | | | | | Unidentified | | C9H12O3 | | | | | | >0.05 | <2 | | **1.97E-02** | | **-6.17** | | **1.29E-02** | | | | **-6.40** | |  |
|  | 174.1402 | | | | | 1.12 | | | | | Unidentified | | C13H18 | | | | | | >0.05 | <2 | | **3.33E-03** | | **-6.09** | | **1.56E-04** | | | | **-6.85** | |  |
|  | 178.0841 | | | | | 1.66 | | | | | Unidentified | | C7H14O5 | | | | | | >0.05 | **-2.04** | | **1.93E-03** | | **-6.03** | | **6.44E-02** | | | | **-3.97** | |  |
|  | 180.0648 | | | | | 2.6 | | | | | Unidentified | | C6H12O6 | | | | | | >0.05 | <2 | | **2.90E-03** | | **-9.53** | | **6.94E-02** | | | | **-5.50** | |  |
|  | 182.0708 | | | | | 1.66 | | | | | Unidentified | | C6H15O4P | | | | | | >0.05 | **3.54** | | **1.73E-03** | | **8.56** | | **3.92E-02** | | | | **5.01** | |  |
|  | 183.9781 | | | | | 30.71 | | | | | Unidentified | | C3H5O7P | | | | | | >0.05 | <2 | | >0.05 | | **-4.15** | | **5.06E-03** | | | | **-7.04** | |  |
|  | 184.1212 | | | | | 8.67 | | | | | Unidentified | | C14H16 | | | | | | >0.05 | <2 | | **1.02E-04** | | **-11.25** | | **5.89E-04** | | | | **-9.83** | |  |
|  | 194.1153 | | | | | 4 | | | | | Unidentified | | C8H18O5 | | | | | | **5.48E-02** | **2.02** | | **7.98E-02** | | **-4.55** | | >0.05 | | | | <2 | |  |
|  | 196.1209 | | | | | 2.45 | | | | | Unidentified | | C15H16 | | | | | | >0.05 | **-3.19** | | **3.12E-03** | | **-7.34** | | **1.77E-02** | | | | **-5.93** | |  |
|  | 197.0898 | | | | | 4.33 | | | | | Unidentified | | C12H11N3 | | | | | | >0.05 | <2 | | **2.25E-03** | | **-8.86** | | **1.01E-02** | | | | **-7.23** | |  |
|  | 199.1574 | | | | | 1.25 | | | | | Unidentified | | C11H21NO2 | | | | | | **5.53E-02** | **4.44** | | **8.80E-02** | | **3.85** | | **4.52E-02** | | | | **3.78** | |  |
|  | 202.0453 | | | | | 4.35 | | | | | Unidentified | | C15H22 | | | | | | **9.97E-02** | **-3.73** | | **1.36E-04** | | **-11.73** | | **4.13E-03** | | | | **-8.54** | |  |
|  | 208.0942 | | | | | 1.97 | | | | | Unidentified | | C8H16O6 | | | | | | >0.05 | <2 | | **3.34E-03** | | **-6.44** | | **2.33E-04** | | | | **-7.25** | |  |
|  | 211.1569 | | | | | 1.41 | | | | | Unidentified | | C12H21NO2 | | | | | | >0.05 | <2 | | **8.89E-04** | | **-7.51** | | **1.91E-02** | | | | **-5.53** | |  |
|  | 213.1724 | | | | | 1.27 | | | | | Unidentified | | C12H23NO2 | | | | | | >0.05 | <2 | | **9.69E-03** | | **-6.11** | | **5.07E-02** | | | | **-4.70** | |  |
|  | 215.1884 | | | | | 1.82 | | | | | Unidentified | | C12H25NO2 | | | | | | >0.05 | <2 | | **2.02E-04** | | **-8.84** | | **2.63E-04** | | | | **-8.46** | |  |
|  | 220.1673 | | | | | 1.57 | | | | | Unidentified | | C11H24O4 | | | | | | >0.05 | <2 | | **5.30E-04** | | **-7.81** | | **1.12E-04** | | | | **-8.17** | |  |
| ******* | 222.1463 | | | | | 2.8 | | | | | Unidentified | | C17H18 | | | | | | >0.05 | <2 | | **1.55E-03** | | **-7.81** | | **2.53E-03** | | | | **-7.23** | |  |
| ****** | 225.1729 | | | | | 1.24 | | | | | Unidentified | | C13H23NO2 | | | | | | >0.05 | <2 | | **3.12E-04** | | **-9.12** | | **7.99E-04** | | | | **-8.42** | |  |
|  | 226.1682 | | | | | 7.65 | | | | | Unidentified | | C12H22N2O2 | | | | | | **7.77E-03** | **-4.74** | | **3.64E-08** | | **-12.40** | | **5.90E-03** | | | | **-7.65** | |  |
|  | 227.1883 | | | | | 1.23 | | | | | Unidentified | | C13H25NO2 | | | | | | >0.05 | **-2.74** | | **2.35E-04** | | **-9.04** | | **7.93E-03** | | | | **-5.76** | |  |
|  | 230.0708 | | | | | 1.1 | | | | | Unidentified | | C17H10O | | | | | | >0.05 | <2 | | **7.58E-04** | | **8.25** | | **3.76E-03** | | | | **6.84** | |  |
|  | 234.1621 | | | | | 1.13 | | | | | Unidentified | | C15H22O2 | | | | | | >0.05 | <2 | | **8.51E-03** | | **-6.77** | | >0.05 | | | | **-3.04** | |  |
|  | 238.1414 | | | | | 5.16 | | | | | Unidentified | | C16H18N2 | | | | | | >0.05 | <2 | | **1.78E-02** | | **-5.38** | | >0.05 | | | | **-1.67** | |  |
|  | 239.173 | | | | | 3.95 | | | | | Unidentified | | C17H21N | | | | | | >0.05 | <2 | | **3.75E-03** | | **-6.99** | | **1.16E-02** | | | | **-6.08** | |  |
|  | 243.2565 | | | | | 13.26 | | | | | Unidentified | | C15H33NO | | | | | | **2.79E-02** | **4.87** | | **2.39E-04** | | **8.48** | | **4.31E-02** | | | | **3.61** | |  |
|  | 246.0852 | | | | | 2.68 | | | | | Unidentified | | C14H14O4 | | | | | | >0.05 | <2 | | **2.31E-03** | | **-7.33** | | **3.91E-03** | | | | **-6.71** | |  |
|  | 248.1986 | | | | | 1.36 | | | | | Unidentified | | C13H28O4 | | | | | | >0.05 | **-2.36** | | **2.57E-03** | | **-6.73** | | **7.01E-04** | | | | **-6.98** | |  |
|  | 252.1571 | | | | | 4.46 | | | | | Unidentified | | C17H20N2 | | | | | | >0.05 | <2 | | **2.87E-03** | | **-7.04** | | **9.14E-04** | | | | **-7.33** | |  |
| ****** | 253.2408 | | | | | 1.27 | | | | | Unidentified | | C16H31NO | | | | | | >0.05 | <2 | | **4.21E-03** | | **6.34** | | **8.66E-02** | | | | **3.40** | |  |
|  | 254.1518 | | | | | 1.12 | | | | | Unidentified | | C14H22O4 | | | | | | >0.05 | <2 | | **3.04E-03** | | **-6.50** | | **9.40E-06** | | | | **-7.88** | |  |
| ******* | 258.1986 | | | | | 1.07 | | | | | Unidentified | | C16H24N3 | | | | | | >0.05 | <2 | | **1.71E-04** | | **-7.85** | | **7.69E-08** | | | | **-9.17** | |  |
|  | 259.0718 | | | | | 4.3 | | | | | Unidentified | | C13H11N2O4 | | | | | | **5.55E-02** | **-4** | | **2.91E-03** | | **-6.29** | | **1.36E-02** | | | | **-5.32** | |  |
|  | 264.2458 | | | | | 1.27 | | | | | Unidentified | | C18H32O | | | | | | >0.05 | <2 | | **4.90E-03** | | **6.66** | | **1.65E-02** | | | | **5.62** | |  |
|  | 268.2194 | | | | | 1.14 | | | | | Unidentified | | C15H28N2O2 | | | | | | >0.05 | <2 | | **2.24E-02** | | **-3.41** | | **5.08E-03** | | | | <2 | |  |
|  | 275.0673 | | | | | 4.26 | | | | | Unidentified | | C10H13NO8 | | | | | | >0.05 | <2 | | **6.44E-03** | | **-6.84** | | **4.67E-02** | | | | **-5.02** | |  |
|  | 280.0224 | | | | | 4.29 | | | | | Unidentified | | C11H2N7O3 | | | | | | >0.05 | <2 | | **4.11E-04** | | **-7.55** | | **3.37E-03** | | | | **-6.45** | |  |
|  | 282.1963 | | | | | 1.13 | | | | | Unidentified | | C20H26O | | | | | | >0.05 | **3.5** | | **1.89E-04** | | **-8.07** | | **2.07E-04** | | | | **-7.74** | |  |
| ******* | 283.2869 | | | | | 1.24 | | | | | Unidentified | | C18H37NO | | | | | | >0.05 | <2 | | **3.09E-03** | | **-8.50** | | **4.45E-04** | | | | **-9.20** | |  |
|  | 292.2404 | | | | | 1.1 | | | | | Unidentified | | C19H32O2 | | | | | | >0.05 | <2 | | **3.39E-02** | | **-5.35** | | **3.56E-04** | | | | **-7.94** | |  |
|  | 294.2041 | | | | | 2.66 | | | | | Unidentified | | C20H26N2 | | | | | | **8.55E-02** | **2.34** | | **1.55E-03** | | **5.86** | | **6.30E-03** | | | | **4.48** | |  |
|  | 297.3032 | | | | | 1.32 | | | | | Unidentified | | C19H39NO | | | | | | >0.05 | <2 | | **2.27E-02** | | **5.42** | | **2.75E-03** | | | | **6.50** | |  |
|  | 298.192 | | | | | 1.15 | | | | | Unidentified | | C20H26O2 | | | | | | **8.75E-02** | **3.26** | | **1.19E-03** | | **-7.78** | | **8.24E-08** | | | | **-10.13** | |  |
|  | 307.2513 | | | | | 1.25 | | | | | Unidentified | | C20H30O2 | | | | | | >0.05 | <2 | | **7.26E-04** | | **-8.08** | | **4.85E-03** | | | | **-6.59** | |  |
| ******* | 308.2195 | | | | | 2.3 | | | | | Unidentified | | C15H32O6 | | | | | | >0.05 | <2 | | **3.37E-04** | | **-9.32** | | **1.49E-02** | | | | **-6.28** | |  |
|  | 310.2152 | | | | | 1.12 | | | | | Unidentified | | C18H30O4 | | | | | | >0.05 | <2 | | **4.24E-03** | | **-6.85** | | **4.83E-02** | | | | **-4.74** | |  |
| ****** | 314.1268 | | | | | 1.18 | | | | | Unidentified | | C17H18N2O4 | | | | | | >0.05 | <2 | | **2.02E-03** | | **-7.37** | | **1.81E-04** | | | | **-8.76** | |  |
|  | 316.2038 | | | | | 1.14 | | | | | Unidentified | | C20H28O3 | | | | | | >0.05 | <2 | | >0.05 | | **-3.96** | | **1.56E-03** | | | | **-7.22** | |  |
|  | 322.1934 | | | | | 1.22 | | | | | Unidentified | | C22H26O2 | | | | | | >0.05 | <2 | | **7.29E-02** | | **-4.69** | | **1.19E-04** | | | | **-8.61** | |  |
|  | 325.3715 | | | | | 12.74 | | | | | Unidentified | | C22H47N | | | | | | **6.71E-02** | **4.36** | | **1.72E-03** | | **7.89** | | **9.83E-02** | | | | **3.52** | |  |
|  | 326.194 | | | | | 7.31 | | | | | Unidentified | | C21H26O3 | | | | | | **8.76E-02** | **2.43** | | **3.18E-05** | | **-8.99** | | **2.93E-03** | | | | **-6.88** | |  |
|  | 330.2199 | | | | | 1.15 | | | | | Unidentified | | C21H30O3 | | | | | | >0.05 | <2 | | **1.24E-02** | | **-6.37** | | **1.18E-05** | | | | **-8.80** | |  |
|  | 334.2145 | | | | | 1.12 | | | | | Unidentified | | C20H30O4 | | | | | | >0.05 | <2 | | **2.64E-06** | | **-11.31** | | **3.18E-06** | | | | **-10.65** | |  |
|  | 336.2145 | | | | | 1.37 | | | | | Unidentified | | C22H28N2O | | | | | | >0.05 | <2 | | **8.73E-03** | | **-6.04** | | **9.57E-03** | | | | **-5.77** | |  |
| ******* | 338.2303 | | | | | 3.29 | | | | | Unidentified | | C23H30O2 | | | | | | >0.05 | **-2.17** | | **7.45E-03** | | **-7.47** | | **9.31E-03** | | | | **-7.00** | |  |
|  | 342.2191 | | | | | 1.13 | | | | | Unidentified | | C22H30O3 | | | | | | >0.05 | <2 | | **8.96E-02** | | **-4.29** | | **2.48E-03** | | | | **-6.32** | |  |
|  | 343.2204 | | | | | 7.31 | | | | | Unidentified | | C21H29NO3 | | | | | | >0.05 | <2 | | **7.60E-04** | | **-7.38** | | **2.35E-02** | | | | **-5.29** | |  |
|  | 344.1218 | | | | | 2.06 | | | | | Unidentified | | C19H20O6 | | | | | | >0.05 | **-2.93** | | **3.74E-02** | | **-5.34** | | **3.23E-03** | | | | **-6.73** | |  |
|  | 348.2301 | | | | | 1.18 | | | | | Unidentified | | C21H32O4 | | | | | | >0.05 | <2 | | **2.73E-03** | | **-7.80** | | **4.09E-04** | | | | **-8.62** | |  |
|  | 352.2098 | | | | | 1.81 | | | | | Unidentified | | C22H28N2O2 | | | | | | **7.83E-02** | **3.37** | | **8.63E-03** | | **-7.21** | | **6.00E-02** | | | | **-5.04** | |  |
|  | 354.2168 | | | | | 1.21 | | | | | Unidentified | | C23H30O3 | | | | | | **7.94E-02** | **3.85** | | **2.02E-03** | | **6.69** | | **4.39E-02** | | | | **3.89** | |  |
| ******* | 355.2563 | | | | | 2.65 | | | | | Unidentified | | C15H31N8O2 | | | | | | >0.05 | <2 | | **1.30E-02** | | **-5.65** | | **8.28E-03** | | | | **-6.11** | |  |
|  | 357.3611 | | | | | 13.06 | | | | | Unidentified | | C22H47NO2 | | | | | | >0.05 | <2 | | **2.58E-04** | | **8.72** | | **2.62E-02** | | | | **4.28** | |  |
|  | 360.1932 | | | | | 1.34 | | | | | Unidentified | | C15H28N4O6 | | | | | | >0.05 | **-3.04** | | **2.56E-03** | | **-6.73** | | **6.22E-04** | | | | **-7.06** | |  |
|  | 362.2958 | | | | | 1.08 | | | | | Unidentified | | C27H38 | | | | | | >0.05 | <2 | | **2.62E-03** | | **-6.36** | | **7.54E-04** | | | | **-6.58** | |  |
|  | 364.2245 | | | | | 2.26 | | | | | Unidentified | | C21H32O5 | | | | | | >0.05 | <2 | | **2.79E-03** | | **-6.49** | | **1.01E-02** | | | | **-5.60** | |  |
|  | 366.2612 | | | | | 2.36 | | | | | Unidentified | | C24H34N2O | | | | | | **7.66E-02** | **-3.66** | | **3.49E-03** | | **-7.49** | | **3.88E-02** | | | | **-5.22** | |  |
|  | 368.2408 | | | | | 4.92 | | | | | Unidentified | | C24H32O3 | | | | | | >0.05 | <2 | | **2.26E-06** | | **-11.74** | | **9.19E-06** | | | | **-10.80** | |  |
|  | 369.2723 | | | | | 2.33 | | | | | Unidentified | | C24H35NO2 | | | | | | >0.05 | <2 | | **6.11E-02** | | **-4.87** | | **5.18E-03** | | | | **-7.03** | |  |
|  | 376.3192 | | | | | 1.35 | | | | | Unidentified | | C25H44O2 | | | | | | >0.05 | <2 | | **4.40E-03** | | **-7.11** | | **1.83E-02** | | | | **-5.71** | |  |
|  | 383.2522 | | | | | 1.95 | | | | | Unidentified | | C17H37NO8 | | | | | | >0.05 | <2 | | **2.34E-03** | | **-6.49** | | **9.32E-06** | | | | **-7.77** | |  |
| ******* | 385.2676 | | | | | 4.92 | | | | | Unidentified | | C16H33N8O3 | | | | | | >0.05 | <2 | | **1.77E-05** | | **-10.61** | | **7.89E-05** | | | | **-9.62** | |  |
|  | 388.1038 | | | | | 1.04 | | | | | Unidentified | | C19H18NO8 | | | | | | >0.05 | <2 | | **6.73E-03** | | **-6.51** | | **9.24E-02** | | | | **-3.77** | |  |
|  | 410.2874 | | | | | 2.46 | | | | | Unidentified | | C27H38O3 | | | | | | >0.05 | <2 | | **9.02E-03** | | **-7.25** | | **4.75E-04** | | | | **-8.64** | |  |
|  | 411.3183 | | | | | 2.08 | | | | | Unidentified | | C27H41NO2 | | | | | | >0.05 | <2 | | **3.17E-03** | | **-6.29** | | **9.28E-06** | | | | **-7.65** | |  |
|  | 413.2986 | | | | | 3.52 | | | | | Unidentified | | C26H39NO3 | | | | | | >0.05 | <2 | | **2.58E-02** | | **5.01** | | >0.05 | | | | **2.23** | |  |
|  | 415.357 | | | | | 1.02 | | | | | Unidentified | | C26H45N3O | | | | | | **5.47E-02** | <2 | | **3.08E-03** | | **-7.40** | | >0.05 | | | | **-3.40** | |  |
|  | 420.3452 | | | | | 1.5 | | | | | Unidentified | | C23H48O6 | | | | | | >0.05 | <2 | | **8.81E-03** | | **-6.59** | | **1.10E-02** | | | | **-6.33** | |  |
|  | 424.2682 | | | | | 1.59 | | | | | Unidentified | | C21H36N4O5 | | | | | | >0.05 | <2 | | **8.96E-05** | | **-8.03** | | **1.02E-06** | | | | **-8.69** | |  |
|  | 432.3227 | | | | | 1.73 | | | | | Unidentified | | C27H44O4 | | | | | | >0.05 | <2 | | **2.39E-03** | | **-6.59** | | **3.35E-02** | | | | **-4.77** | |  |
|  | 434.3606 | | | | | 1.49 | | | | | Unidentified | | C24H50O6 | | | | | | >0.05 | <2 | | **7.17E-03** | | **-6.84** | | **1.17E-02** | | | | **-6.19** | |  |
|  | 441.3444 | | | | | 1.27 | | | | | Unidentified | | C23H45N4O4 | | | | | | >0.05 | <2 | | **4.99E-05** | | **-9.61** | | **4.27E-05** | | | | **-9.47** | |  |
|  | 443.309 | | | | | 5.07 | | | | | Unidentified | | C21H41N5O5 | | | | | | >0.05 | <2 | | **1.54E-03** | | **-8.39** | | **1.09E-04** | | | | **-9.26** | |  |
|  | 446.3965 | | | | | 1.27 | | | | | Unidentified | | C26H54O5 | | | | | | >0.05 | <2 | | **6.15E-03** | | **-6.57** | | **9.95E-03** | | | | **-5.93** | |  |
|  | 448.3762 | | | | | 1.46 | | | | | Unidentified | | C24H46N7O | | | | | | >0.05 | **-3.2** | | **3.77E-02** | | **-5.19** | | **1.11E-02** | | | | **-5.89** | |  |
|  | 457.4139 | | | | | 12.77 | | | | | Unidentified | | C27H55NO4 | | | | | | >0.05 | <2 | | **2.43E-04** | | **9.78** | | **2.61E-02** | | | | **4.87** | |  |
|  | 464.3707 | | | | | 1.65 | | | | | Unidentified | | C24H46N7O2 | | | | | | >0.05 | <2 | | **1.30E-02** | | **-5.65** | | **8.28E-03** | | | | **-6.11** | |  |
|  | 468.3299 | | | | | 2.92 | | | | | Unidentified | | C30H44O4 | | | | | | >0.05 | <2 | | **3.99E-04** | | **-7.29** | | **1.06E-03** | | | | **-6.73** | |  |
|  | 470.309 | | | | | 5.98 | | | | | Unidentified | | C21H40N7O5 | | | | | | >0.05 | <2 | | **1.01E-04** | | **-9.49** | | **2.31E-04** | | | | **-8.67** | |  |
|  | 480.366 | | | | | 1.49 | | | | | Unidentified | | C32H48O3 | | | | | | >0.05 | <2 | | **1.58E-02** | | **-6.04** | | **1.04E-04** | | | | **-8.21** | |  |
|  | 482.3455 | | | | | 2.22 | | | | | Unidentified | | C31H46O4 | | | | | | >0.05 | <2 | | **8.19E-03** | | **-7.19** | | **6.82E-02** | | | | **-4.65** | |  |
| ******* | 483.3409 | | | | | 1.83 | | | | | Unidentified | | C23H49NO9 | | | | | | >0.05 | **2.15** | | **3.09E-02** | | **4.63** | | **1.30E-02** | | | | **4.91** | |  |
|  | 485.371 | | | | | 1.39 | | | | | Unidentified | | C26H45N8O | | | | | | >0.05 | <2 | | **9.05E-03** | | **-6.73** | | **1.75E-03** | | | | **-7.78** | |  |
|  | 490.4221 | | | | | 1.38 | | | | | Unidentified | | C26H56N3O5 | | | | | | >0.05 | **2.3** | | **6.73E-03** | | **-6.32** | | **4.13E-02** | | | | **-4.73** | |  |
|  | 497.3568 | | | | | 1.66 | | | | | Unidentified | | C24H51NO9 | | | | | | >0.05 | <2 | | **8.51E-03** | | **-5.90** | | **8.54E-03** | | | | **-5.66** | |  |
|  | 513.3869 | | | | | 2.54 | | | | | Unidentified | | C24H49N8O4 | | | | | | >0.05 | <2 | | **9.42E-03** | | **-5.63** | | **9.46E-06** | | | | **-7.54** | |  |
|  | 514.35 | | | | | 1.74 | | | | | Unidentified | | C27H44N7O3 | | | | | | >0.05 | <2 | | **1.24E-02** | | **-6.18** | | **1.05E-02** | | | | **-6.07** | |  |
|  | 515.3661 | | | | | 3.56 | | | | | Unidentified | | C27H45N7O3 | | | | | | >0.05 | <2 | | **1.66E-04** | | **-8.60** | | **6.82E-04** | | | | **-7.71** | |  |
|  | 525.3872 | | | | | 1.39 | | | | | Unidentified | | C25H49N8O4 | | | | | | >0.05 | <2 | | **9.27E-04** | | **-7.34** | | **1.58E-03** | | | | **-6.75** | |  |
|  | 526.3717 | | | | | 2.6 | | | | | Unidentified | | C33H50O5 | | | | | | >0.05 | <2 | | **5.97E-02** | | **-5.14** | | **5.06E-03** | | | | **-7.06** | |  |
|  | 529.3822 | | | | | 3.09 | | | | | Unidentified | | C24H49N8O5 | | | | | | >0.05 | <2 | | **4.86E-04** | | **-8.84** | | **3.87E-03** | | | | **-7.37** | |  |
| ****** | 534.4492 | | | | | 1.54 | | | | | Unidentified | | C29H56N7O2 | | | | | | >0.05 | <2 | | **9.65E-03** | | **-5.94** | | **7.30E-03** | | | | **-6.01** | |  |
|  | 540.3867 | | | | | 2.3 | | | | | Unidentified | | C26H50N7O5 | | | | | | >0.05 | <2 | | **4.00E-03** | | **-8.16** | | **6.52E-02** | | | | **-4.97** | |  |
|  | 541.3835 | | | | | 1.87 | | | | | Unidentified | | C27H51N5O6 | | | | | | **9.72E-02** | **-2.92** | | **2.72E-03** | | **-6.71** | | **1.32E-04** | | | | **-7.57** | |  |
| ****** | 550.4166 | | | | | 1.12 | | | | | Unidentified | | C40H54O | | | | | | **8.73E-02** | **4.79** | | **2.90E-03** | | **-7.02** | | **2.16E-01** | | | | **-3.22** | |  |
|  | 553.4551 | | | | | 1.79 | | | | | Unidentified | | C28H57N8O3 | | | | | | >0.05 | <2 | | **1.10E-02** | | **-6.61** | | **1.72E-02** | | | | **-5.96** | |  |
|  | 554.4028 | | | | | 2.07 | | | | | Unidentified | | C27H52N7O5 | | | | | | >0.05 | <2 | | **2.52E-03** | | **-8.56** | | **1.78E-02** | | | | **-6.65** | |  |
|  | 554.4033 | | | | | 1.9 | | | | | Unidentified | | C28H58O10 | | | | | | >0.05 | <2 | | **1.27E-03** | | **8.50** | | **7.14E-03** | | | | **6.74** | |  |
|  | 555.3985 | | | | | 1.68 | | | | | Unidentified | | C26H51N8O5 | | | | | | >0.05 | <2 | | **7.44E-02** | | **-4.36** | | **2.76E-03** | | | | **-6.51** | |  |
|  | 559.3925 | | | | | 5.29 | | | | | Unidentified | | C25H51N8O6 | | | | | | **2.58E-02** | **5.2** | | **6.16E-04** | | **8.14** | | **1.56E-03** | | | | **7.29** | |  |
|  | 571.4284 | | | | | 2.69 | | | | | Unidentified | | C27H55N8O5 | | | | | | >0.05 | **-3.35** | | >0.05 | | **-3.95** | | **2.91E-03** | | | | **-6.25** | |  |
|  | 573.4231 | | | | | 1.72 | | | | | Unidentified | | C29H57N4O7 | | | | | | >0.05 | <2 | | **1.87E-03** | | **-7.40** | | **1.73E-04** | | | | **-8.56** | |  |
|  | 578.4753 | | | | | 1.7 | | | | | Unidentified | | C36H66O5 | | | | | | >0.05 | <2 | | **7.11E-04** | | **-7.06** | | **6.09E-03** | | | | **-5.80** | |  |
|  | 581.4205 | | | | | 1.03 | | | | | Unidentified | | C37H57O5 | | | | | | >0.05 | <2 | | **8.81E-04** | | **-8.42** | | **5.18E-06** | | | | **-10.06** | |  |
|  | 584.4131 | | | | | 2.78 | | | | | Unidentified | | C28H54N7O6 | | | | | | >0.05 | **-2.32** | | **5.47E-04** | | **-8.87** | | **1.16E-03** | | | | **-8.10** | |  |
|  | 595.473 | | | | | 1.23 | | | | | Unidentified | | C39H63O4 | | | | | | >0.05 | <2 | | **8.31E-04** | | **7.81** | | **1.55E-02** | | | | **5.17** | |  |
|  | 596.4476 | | | | | 1.54 | | | | | Unidentified | | C27H60N6O8 | | | | | | >0.05 | <2 | | **3.03E-03** | | **-6.50** | | **4.06E-02** | | | | **-4.50** | |  |
|  | 599.4252 | | | | | 1.9 | | | | | Unidentified | | C30H57N5O7 | | | | | | >0.05 | <2 | | **5.43E-03** | | **-6.41** | | **6.43E-03** | | | | **-6.12** | |  |
|  | 601.4397 | | | | | 2.78 | | | | | Unidentified | | C28H57N8O6 | | | | | | >0.05 | <2 | | **1.25E-04** | | **-9.85** | | **1.15E-02** | | | | **-6.93** | |  |
|  | 603.4177 | | | | | 6.2 | | | | | Unidentified | | C24H57N7O10 | | | | | | >0.05 | **3.52** | | **1.30E-04** | | **9.20** | | **9.95E-05** | | | | **8.85** | |  |
|  | 607.3882 | | | | | 13.65 | | | | | Unidentified | | C38H49N5O2 | | | | | | >0.05 | <2 | | **5.99E-06** | | **-8.68** | | **1.04E-06** | | | | **-8.68** | |  |
|  | 609.3018 | | | | | 14.89 | | | | | Unidentified | | C35H39N5O5 | | | | | | >0.05 | <2 | | **2.50E-04** | | **9.23** | | **8.87E-03** | | | | **5.84** | |  |
|  | 626.4604 | | | | | 1.88 | | | | | Unidentified | | C31H60N7O6 | | | | | | >0.05 | <2 | | **2.16E-03** | | **-8.25** | | **1.13E-02** | | | | **-6.64** | |  |
|  | 629.471 | | | | | 2.07 | | | | | Unidentified | | C30H61N8O6 | | | | | | >0.05 | <2 | | **5.68E-03** | | **-8.27** | | **7.51E-02** | | | | **-4.92** | |  |
|  | 631.4499 | | | | | 3.97 | | | | | Unidentified | | C29H59N8O7 | | | | | | >0.05 | <2 | | **9.84E-05** | | **-9.06** | | **4.80E-04** | | | | **-8.11** | |  |
|  | 642.5329 | | | | | 1.08 | | | | | Unidentified | | C38H68N5O3 | | | | | | >0.05 | **3.47** | | **3.07E-04** | | **-6.82** | | **1.33E-02** | | | | **-5.15** | |  |
|  | 644.5513 | | | | | 1.11 | | | | | Unidentified | | C45H72O2 | | | | | | >0.05 | <2 | | **4.74E-03** | | **-6.56** | | **7.62E-03** | | | | **-6.01** | |  |
|  | 649.4321 | | | | | 13.05 | | | | | Unidentified | | C33H64NO9P | | | | | | >0.05 | **3.39** | | **2.42E-04** | | **9.51** | | **8.76E-03** | | | | **5.99** | |  |
|  | 653.2906 | | | | | 14.97 | | | | | Unidentified | | C42H35N7O | | | | | | >0.05 | **2.02** | | **6.77E-03** | | **-6.91** | | **8.97E-03** | | | | **-6.39** | |  |
|  | 659.4168 | | | | | 13.34 | | | | | Unidentified | | C31H53N11O5 | | | | | | >0.05 | <2 | | **2.19E-06** | | **-10.91** | | **9.72E-11** | | | | **-12.32** | |  |
|  | 661.4602 | | | | | 6.31 | | | | | Unidentified | | C35H68NO8P | | | | | | >0.05 | <2 | | **5.93E-04** | | **-7.92** | | **9.78E-06** | | | | **-8.72** | |  |
| ****** | 662.5625 | | | | | 1.1 | | | | | Unidentified | | C42H78O5 | | | | | | >0.05 | <2 | | **5.99E-03** | | **-6.52** | | **7.18E-03** | | | | **-6.06** | |  |
|  | 666.5365 | | | | | 1.06 | | | | | Unidentified | | C31H70N8O7 | | | | | | **5.47E-02** | **2.56** | | **4.31E-04** | | **-8.41** | | **7.96E-05** | | | | **-8.80** | |  |
|  | 670.4863 | | | | | 2.14 | | | | | Unidentified | | C38H71O7P | | | | | | **7.81E-02** | **4** | | **1.70E-03** | | **-8.57** | | **1.81E-02** | | | | **-6.32** | |  |
|  | 676.5436 | | | | | 1.07 | | | | | Unidentified | | C38H77O7P | | | | | | >0.05 | <2 | | **2.19E-05** | | **-9.98** | | **3.51E-06** | | | | **-9.98** | |  |
|  | 682.5326 | | | | | 1.12 | | | | | Unidentified | | C47H70O3 | | | | | | >0.05 | <2 | | **5.12E-02** | | **-5.44** | | **5.45E-03** | | | | **-6.97** | |  |
|  | 683.5543 | | | | | 2.26 | | | | | Unidentified | | C35H71N8O5 | | | | | | >0.05 | <2 | | **6.93E-03** | | **-6.49** | | **8.27E-03** | | | | **-6.27** | |  |
|  | 686.4814 | | | | | 3.5 | | | | | Unidentified | | C38H71O8P | | | | | | **4.66E-02** | **-3.8** | | **2.20E-03** | | **-7.37** | | **1.80E-03** | | | | **-7.10** | |  |
|  | 689.4923 | | | | | 4.1 | | | | | Unidentified | | C37H72NO8P | | | | | | >0.05 | **-2.01** | | **7.65E-04** | | **-8.34** | | **1.17E-04** | | | | **-8.76** | |  |
|  | 714.513 | | | | | 2.49 | | | | | Unidentified | | C40H75O8P | | | | | | >0.05 | **-2.58** | | **3.84E-03** | | **-8.04** | | **1.33E-02** | | | | **-6.60** | |  |
|  | 717.5233 | | | | | 2.94 | | | | | Unidentified | | C39H76NO8P | | | | | | >0.05 | <2 | | **6.72E-04** | | **-9.63** | | **7.09E-03** | | | | **-7.61** | |  |
|  | 721.2792 | | | | | 14.97 | | | | | Unidentified | | C43H33N10O2 | | | | | | >0.05 | **3.47** | | **2.44E-04** | | **9.33** | | **8.80E-03** | | | | **5.86** | |  |
|  | 743.5561 | | | | | 12.12 | | | | | Unidentified | | C41H78NO8P | | | | | | **7.45E-02** | **3.42** | | **2.90E-02** | | **-6.21** | | **9.20E-03** | | | | **-6.88** | |  |
|  | 749.3095 | | | | | 14.72 | | | | | Unidentified | | C40H47NO13 | | | | | | >0.05 | <2 | | **2.46E-04** | | **8.34** | | **2.64E-02** | | | | **4.34** | |  |
| ****** | 781.8172 | | | | | 11.81 | | | | | Unidentified | | C45H99N9O | | | | | | >0.05 | <2 | | **3.11E-03** | | **-8.55** | | **1.02E-06** | | | | **-11.10** | |  |
|  | 782.0276 | | | | | 11.87 | | | | | Unidentified | | C51H10O10 | | | | | | >0.05 | <2 | | **2.18E-03** | | **-7.53** | | **1.66E-03** | | | | **-7.45** | |  |
|  | 789.266 | | | | | 14.97 | | | | | Unidentified | | C43H35N9O7 | | | | | | **4.66E-02** | **4.51** | | **2.44E-04** | | **8.93** | | **8.84E-03** | | | | **5.61** | |  |
|  | 802.5646 | | | | | 3.62 | | | | | Unidentified | | C44H83O10P | | | | | | >0.05 | **2.88** | | **8.75E-04** | | **7.73** | | **1.13E-04** | | | | **8.01** | |  |
|  | 804.6139 | | | | | 13.24 | | | | | Unidentified | | C45H89O9P | | | | | | >0.05 | <2 | | **5.87E-06** | | **-8.62** | | **2.01E-02** | | | | **-5.30** | |  |
|  | 808.1143 | | | | | 11.74 | | | | | Unidentified | | C52H12N10O2 | | | | | | >0.05 | <2 | | **5.81E-04** | | **-6.58** | | **9.13E-06** | | | | **-7.25** | |  |
|  | 810.1999 | | | | | 11.84 | | | | | Unidentified | | C51H24N9O3 | | | | | | >0.05 | <2 | | **2.48E-03** | | **-5.96** | | **1.28E-03** | | | | **-6.04** | |  |
|  | 821.3853 | | | | | 1.02 | | | | | Unidentified | | C57H49N4O2 | | | | | | >0.05 | <2 | | **8.31E-03** | | **-6.72** | | **3.85E-02** | | | | **-5.17** | |  |
| ******* | 833.5936 | | | | | 12.37 | | | | | Unidentified | | C48H84NO8P | | | | | | >0.05 | **-4.03** | | **3.12E-03** | | **-10.02** | | **4.55E-03** | | | | **-9.44** | |  |
|  | 857.2541 | | | | | 14.96 | | | | | Unidentified | | C59H37O7 | | | | | | >0.05 | <2 | | **2.46E-04** | | **8.63** | | **2.67E-02** | | | | **4.12** | |  |
|  | 866.6971 | | | | | 1.07 | | | | | Unidentified | | C55H94O7 | | | | | | >0.05 | <2 | | **1.36E-05** | | **-8.04** | | **7.73E-08** | | | | **-8.64** | |  |
|  | 876.5788 | | | | | 1.03 | | | | | Unidentified | | C46H85O13P | | | | | | >0.05 | <2 | | **8.94E-03** | | **-6.09** | | **2.22E-02** | | | | **-5.22** | |  |
|  | 891.6493 | | | | | 3.18 | | | | | Unidentified | | C57H85N3O5 | | | | | | >0.05 | <2 | | **7.45E-04** | | **-7.48** | | **5.43E-04** | | | | **-7.21** | |  |
|  | 921.6599 | | | | | 4.65 | | | | | Unidentified | | C58H87N3O6 | | | | | | >0.05 | <2 | | **8.16E-04** | | **-8.16** | | **7.42E-05** | | | | **-8.52** | |  |
|  | 949.668 | | | | | 1.02 | | | | | Unidentified | | C61H85N6O3 | | | | | | >0.05 | <2 | | **7.78E-03** | | **-6.70** | | **1.70E-02** | | | | **-5.67** | |  |
|  | 951.6703 | | | | | 6.11 | | | | | Unidentified | | C59H89N3O7 | | | | | | >0.05 | **-3.09** | | **8.00E-04** | | **-8.03** | | **5.24E-04** | | | | **-7.75** | |  |
|  | 968.6975 | | | | | 6.1 | | | | | Unidentified | | C61H94NO8 | | | | | | >0.05 | <2 | | **7.52E-03** | | **-6.25** | | **3.64E-04** | | | | **-7.23** | |  |
|  | 979.7015 | | | | | 4.73 | | | | | Unidentified | | C61H93N3O7 | | | | | | >0.05 | <2 | | **8.21E-04** | | **-7.80** | | **7.45E-05** | | | | **-8.15** | |  |
|  | 988.6488 | | | | | 14.63 | | | | | Unidentified | | C55H86N7O9 | | | | | | >0.05 | **-2.47** | | **2.34E-04** | | **-9.29** | | **6.85E-03** | | | | **-6.82** | |  |

^*^Abbreviations: HILIC-MS/MS - hydrophilic interaction chromatography tandem mass spectrometry; MRM-LC-MS/MS – multiple reaction monitoring LC-MS/MS; DHF/DSS - dengue hemorrhagic syndrome/dengue shock syndrome; DF - dengue fever; ND - non-dengue febrile disease; RT - retention time; FC - fold change; DB – database; MSI - Metabolomics Standard Initiative; HMDB - human metabolome database; LMGP - Lipid maps gateway; KEGG- Kyoto Encyclopedia of Genes and Genomes, METLIN - Metabolite and Tandem Mass Spectrometry Database; NIST - National Institute of Standards and Technology. PS - phosphatidylserine; PC - phosphatidylcholine; DG – diacylglycerol; TG – triglyceride; HETE – hydroxyeicosatetraenoic acid; PE – phosphatidylethanolamine; PA–phosphatidic acid; Cer – ceramide; PI – phosphatidylinositol; CE – cholesteryl ester; SM– sphingomyelin. ^**^ Metabolites also detected in Nicaraguan samples with concordant fold change directions.^***^Metabolites also detected in Nicaraguan samples but with non-concordant fold change directions.^****^Ionized form of metabolites: myristoleic acid [M+H-H2O]^+^ = 226.1932; phosphatidylcholine (34:1) [M+Na-2H]^+^ = 781.5627; phosphatidylcholine (34:0) [M+H-Na]^+^ =784.5827; all other metabolites listed were [M+H]^+^.
