# Supplementary material for: Metabolomics-Based Discovery of Small Molecule Biomarkers in Serum Associated with Dengue Virus Infections and Disease Outcomes
Source: PLoS Negl Trop Dis. 2016 Feb 25;10(2):e0004449. doi: 10.1371/journal.pntd.0004449 (PMC4768770; doi:10.1371/journal.pntd.0004449)
Supplement: S3 Table — MSI Level 1: Metabolites identified by HILIC- MS/MS*or MRM-LC-MS/MS spectra matches with spectra of chemical reference standards acquired on the same analytical platform. MSI Level 3: Metabolites putatively identified based on physicochemical characteristics of a chemical class of compounds or by spectrum similarity to known compounds. MSI Level 4: Unidentified or unclassified metabolites that can be differentiated or quantified based in spectrum data. Bolded values indicate statistically significant differences in pairwise comparison of the two diagnosis groups. (DOCX) [file pntd.0004449.s013.docx]

**S3 Table. Metabolites in Nicaraguan sera collected < 4 days after onset of symptoms that predicted progression of DF patients to DHF/DSS.** MSI Level 1: Metabolites identified by HILIC- MS/MS*or MRM-LC-MS/MS spectra matches with spectra of chemical reference standards acquired on the same analytical platform. MSI Level 3: Metabolites putatively identified based on physicochemical characteristics of a chemical class of compounds or by spectrum similarity to known compounds. MSI Level 4: Unidentified or unclassified metabolites that can be differentiated or quantified based in spectrum data. Bolded values indicate statistically significant differences in pairwise comparison of the two diagnosis groups.

| **MSI level 1** | | | | | | | | |
| --- | --- | --- | --- | --- | --- | --- | --- | --- |
| **Mass** | **RT** | | **Metabolite** | **Chemical formula** | | **DB identifier*** | **DF TO DHF/DSS vs. DF** | |
|  |  |  |  |  |  |  | **P value** | **FC** |
| 115.0635 | 16.45 | | Proline | C5H9NO2 | | HMDB00162 | **1.65E-02** | <2 |
| 278.2245 | 1.10 | | Alpha-linolenic acid | C18H30 O2 | | Metlin 192 | **2.49E-14** | **4.21** |
| 302.2243 | 1.13 | | Arachidonic acid | C20H30O2 | | Metlin 35293 | **1.27E-02** | **2.00** |
| 328.238 | 1.09 | | Docosahexaenoic acid | C22 H32 O2 | | Kegg C06429 | **1.48E-11** | **3.04** |
| 495.3326 | 13.74 | | LysoPC (16:0) | C24H50NO7P | | Metlin 61692 | **2.91E-03** | <2 |
| 521.3492 | 13.54 | | LysoPC (18:1) | C26H52NO7P | | Metlin 61695 | **5.17E-12** | **2.24** |
| **MSI level 3** | | | | | | | | |
| **Mass** | **RT** | **Metabolite** | | **Chemical formula** | **#DB hits** | **DB identifier*** | **DF TO DHF/DSS vs. DF** | |
|  |  |  |  |  |  |  | **P value** | **FC** |
| 276.2087 | 1.11 | Octadecadienoic acid | | C18H28O2 | >5 | Kegg C16300 | **1.08E-04** | **2.28** |
| 318.2191 | 1.19 | Hydroxy-eicosapentaenoic acid | | C20H30O3 | >5 | Metlin 35313 | **1.04E-04** | **2.92** |
| 458.2723 | 6.52 | Arg Arg Gln | | C18H34N8O6 | >5 | Metlin 16788 | >0.05 | **-3.28** |
| 481.2857 | 1.92 | PC(14:0) | | C22H44NO8P | 1 | Metlin 39145 | >0.05 | **2.66** |
| 509.3831 | 14.94 | PC(18:0) | | C26H56NO6P | >5 | LMGP01040041 | **1.88E-04** | <2 |
| 523.364 | 13.61 | PC(18:0) | | C26H54NO7P | >5 | Metlin 40075 | **9.49E-05** | **2.06** |
| 591.4939 | 1.06 | DG(34:0) | | C37H67D5O5 | 1 | Metlin 4687 | **0.00E+00** | **2.84** |
| 638.401 | 1.92 | PA(32:5) | | C35H59O8P | 4 | LMGP10010061 | **2.58E-15** | **2.48** |
| 779.5482 | 11.98 | PC(38:5) | | C44H78NO8P | >5 | HMDB07890 | **8.96E-03** | **-2.05** |
| 813.5529 | 11.34 | PS(38:3) | | C44H80NO10P | >5 | HMDB12382 | >0.05 | **-2.66** |
| 826.7037 | 1.81 | TG(50:4) | | C53H94O6 | >5 | LMGL03010046 | **6.29E-07** | <2 |
| 854.7367 | 2.44 | TG(52:4) | | C55H98O6 | >5 | Metlin 61746 | **4.27E-07** | **3.16** |
| 872.6864 | 1.07 | TG(54:9) | | C57H92O6 | >5 | Metlin 37005 | **1.92E-13** | **3.14** |
| 876.7193 | 1.26 | TG(54:7) | | C57H96O6 | >5 | LMGL03010398 | **4.45E-03** | **2.02** |
| 878.7344 | 1.37 | TG(54:6) | | C57H98O6 | >5 | LMGL03010350 | **8.17E-06** | **2.41** |
| 880.7504 | 2.40 | TG(54:5) | | C57H100O6 | >5 | Metlin 36812 | **5.38E-14** | **3.10** |
| 886.6662 | 1.05 | PG(44:2) | | C50H95O10P | 3 | LMGP04010723 | **1.20E-06** | **2.05** |
| **MSI level 4** | | | | | | | | |
| **Mass** | **RT*** | **Calculated formula** | | | | | **DF TO DHF/DSS vs. DF** | |
|  |  |  |  |  |  |  | **P value** | **FC** |
| 225.0749 | 17.60 | C9H11N3O4 | | | | | **3.65E-05** | **-2.21** |
| 253.1162 | 2.67 | C9H19NO7 | | | | | >0.05 | **2.52** |
| 290.2093 | 1.45 | C15H30O5 | | | | | **2.95E-05** | **2.19** |
| 294.2192 | 1.71 | C18H30O3 | | | | | **4.83E-05** | <2 |
| 320.3187 | 10.03 | C21H40N2 | | | | | **7.81E-05** | **2.28** |
| 321.2777 | 12.57 | C19H35N3O | | | | | >0.05 | **2.86** |
| 323.3184 | 1.38 | C21H41NO | | | | | **7.73E-03** | <2 |
| 325.3706 | 19.15 | C22H47N | | | | | **3.66E-05** | **-2.48** |
| 326.2242 | 1.10 | C22H30O2 | | | | | **4.74E-07** | **2.20** |
| 348.3499 | 9.87 | C23H44N2 | | | | | **1.24E-07** | **2.19** |
| 394.2125 | 32.97 | C19H30N4O5 | | | | | **5.94E-04** | **2.52** |
| 398.3292 | 8.10 | C27H42O2 | | | | | **0.00E+00** | **2.16** |
| 411.0953 | 1.11 | C20H11N8O3 | | | | | **3.32E-03** | **-2.12** |
| 414.2043 | 1.22 | C18H30N4O5S | | | | | **9.01E-03** | <2 |
| 429.2932 | 3.01 | C17H35N9O4 | | | | | **1.95E-04** | <2 |
| 492.3781 | 1.06 | C30H52O5 | | | | | **3.85E-05** | **2.65** |
| 502.2991 | 7.52 | C20H38N8O7 | | | | | **1.28E-03** | **-3.62** |
| 503.867 | 1.59 | C24N4O10 | | | | | **1.65E-03** | **2.28** |
| 543.3612 | 2.94 | C23H45N9O6 | | | | | **3.31E-05** | **-2.54** |
| 572.4784 | 1.07 | C37H64O4 | | | | | **5.05E-05** | <2 |
| 575.2676 | 13.86 | C27H37N5O9 | | | | | >0.05 | **-2.45** |
| 594.4655 | 1.18 | C35H62O7 | | | | | **6.12E-10** | **3.21** |
| 601.3408 | 13.88 | C28H43N9O6 | | | | | **5.47E-10** | <2 |
| 603.3254 | 1.13 | C27H41N9O7 | | | | | **9.83E-06** | **-2.08** |
| 614.3299 | 1.12 | C27H42N12O5 | | | | | **8.12E-05** | **-2.01** |
| 631.356 | 1.12 | C34H45N7O5 | | | | | **5.93E-06** | **-2.30** |
| 642.3612 | 1.11 | C30H59O12P | | | | | **1.76E-04** | **-2.48** |
| 659.3884 | 1.11 | C32H51N8O7 | | | | | **9.91E-05** | <2 |
| 662.5632 | 1.69 | C45H74O3 | | | | | >0.05 | **2.25** |
| 676.543 | 1.22 | C37H77N2O6P | | | | | **4.61E-11** | **3.08** |
| 687.5647 | 1.26 | C38H71N8O3 | | | | | >0.05 | **2.06** |
| 700.5424 | 1.19 | C40H77O7P | | | | | >0.05 | **2.55** |
| 709.6338 | 1.64 | C42H83N3O5 | | | | | **3.23E-04** | <2 |
| 718.5536 | 1.22 | C40H79O8P | | | | | **9.46E-11** | **3.55** |
| 807.8496 | 1.90 | C46H99N10O | | | | | **3.04E-03** | **-2.05** |
| 864.6834 | 1.17 | C55H92O7 | | | | | **7.01E-05** | **2.14** |
| 866.6993 | 1.25 | C55H94O7 | | | | | **6.64E-05** | <2 |
| 880.6808 | 1.11 | C56H88N4O4 | | | | | >0.05 | **3.10** |
| 888.6812 | 1.07 | C50H97O10P | | | | | **1.84E-03** | **2.20** |
| 906.6905 | 1.07 | C51H88N9O5 | | | | | **1.38E-18** | **2.36** |
| 910.6679 | 1.06 | C59H90O7 | | | | | >0.05 | **2.78** |
| 917.8028 | 2.42 | C60H99N7 | | | | | >0.05 | **3.38** |
| 990.6646 | 14.09 | C51H94N2O16 | | | | | **8.55E-04** | <2 |

Abbreviations: HILIC - hydrophilic interaction chromatography; DHF/DSS - dengue hemorrhagic syndrome/dengue shock syndrome; DF - dengue fever; ND - non-dengue febrile disease; RT - retention time; FC - fold change; DB – database; MSI - Metabolomics Standard Initiative; HMDB - human metabolome database; LMGP - Lipid maps gateway; METLIN - Metabolite and Tandem Mass Spectrometry Database; KEGG- Kyoto Encyclopedia of Genes and Genomes PC - phosphatidylcholine; DG – diacylglycerol; TG – triglyceride.
