# Supplementary material for: Metabolomics-Based Discovery of Small Molecule Biomarkers in Serum Associated with Dengue Virus Infections and Disease Outcomes
Source: PLoS Negl Trop Dis. 2016 Feb 25;10(2):e0004449. doi: 10.1371/journal.pntd.0004449 (PMC4768770; doi:10.1371/journal.pntd.0004449)
Supplement: S4 Table — MSI Level 3: Metabolites putatively identified based on physicochemical characteristics of a chemical class of compounds or by spectrum similarity to known compounds. MSI Level 4: Unidentified or unclassified metabolites that can be differentiated or quantified based in spectrum data. Bolded values show those that statistically differentiated pairwise comparisons of the two DENV serotypes. None of the following metabolites have been structurally characterized by LC-MS/MS to be classified at MSI Levels 1 or 2. (DOCX) [file pntd.0004449.s014.docx]

**S4 Table. Mexican acute phase serum metabolites that differentiate DENV-1 and DENV-2 infections.** MSI Level 3: Metabolites putatively identified based on physicochemical characteristics of a chemical class of compounds or by spectrum similarity to known compounds. MSI Level 4: Unidentified or unclassified metabolites that can be differentiated or quantified based in spectrum data. Bolded values show those that statistically differentiated pairwise comparisons of the two DENV serotypes. None of the following metabolites have been structurally characterized by LC-MS/MS to be classified at MSI Levels 1 or 2.

| **MSI level 3** | | | | | | | | | |
| --- | --- | --- | --- | --- | --- | --- | --- | --- | --- |
| **Mass** | | **RT** | **Potential ID** | **Calculated formula** | **#DB hits** | **DB identifier** | | **DENV-2 vs. DENV-1** | |
|  |  |  |  |  |  |  |  | **P value** | **FC** |
| 262.2299 | | 1.37 | Octadecatrienal | C18H30O | >5 | Metlin46529 | | **7.92E-03** | <2 |
| 264.2458 | | 1.27 | Octadecadienal | C18H32O | >5 | Metlin46167 46167 | | **2.65E-03** | **2.30** |
| 280.2401 | | 1.07 | Octadecadienoic acid | C18H32O2 | >5 | Metlin34788 | | **2.32E-03** | **3.53** |
| 283.2869 | | 1.35 | Octadecanamide | C18H37NO | <5 |  | | **5.63E-03** | **-2.33** |
| 295.2872 | | 1.24 | Sphingosine | ‎C18H37NO2 | <5 | LMSP01080013 | | **1.57E-03** | **2.01** |
| 356.2923 | | 1.21 | MG(18:1) | C21H40O4 | <5 | LMGL01010005 | | **5.94E-03** | **2.42** |
| 394.293 | | 1.63 | 25-hydroxy-hexadehydrovitamin D3 | C27H38O2 | <5 | Metlin 42031 | | **1.21E-02** | **2.58** |
| 418.3429 | | 1.10 | 1,25-dihydroxycholesterol | C27H46O3 | >5 | Metlin41686 | | **8.34E-03** | <2 |
| 425.3354 | | 1.56 | 3-Deoxy-25-hydroxyvitamin D3 | C27H43N3O | <5 | LMST03020677 | | **9.73E-03** | **2.05** |
| 450.3278 | | 1.13 | SM(d17:1) | C22H47N2O5P | <5 | Metlin 53899 | | **5.54E-03** | **-6.74** |
| 803.546 | | 11.77 | PC(38:7) | C46H78NO8P | >5 | Metlin39438 | | **9.53E-03** | **2.12** |
| 859.6061 | | 11.66 | PC(42:7) | C50H86NO8P | >5 | Metlin59767 | | **4.85E-03** | <2 |
| **MSI level 4** | | | | | | | | | |
| **Mass** | **RT** | | **Potential ID** | | | | **Calculated formula** | **DENV-2 vs DENV-1** | |
|  |  |  |  |  |  |  |  | **P value** | **FC** |
| 197.1781 | 1.30 | | Unidentified | | | | C12H23NO | **3.74E-05** | **2.07** |
| 285.1363 | 1.33 | | Unidentified | | | | C17H19NO3 | **6.80E-03** | **-3.27** |
| 308.2195 | 2.29 | | Unidentified | | | | C14H26N7O | **9.24E-03** | **2.39** |
| 366.2612 | 2.35 | | Unidentified | | | | C17H32N7O2 | **2.17E-04** | **3.09** |
| 411.0444 | 1.08 | | Unidentified | | | | C31H7O2 | **8.45E-04** | **-2.42** |
| 428.3665 | 1.24 | | Unidentified | | | | C29H48O2 | **8.49E-03** | **2.47** |
| 429.3704 | 0.90 | | Unidentified | | | | C25H45N6 | **6.56E-03** | **4.89** |
| 545.5158 | 1.21 | | Unidentified | | | | C34H65N4O | **1.58E-03** | <2 |
| 562.956 | 1.26 | | Unidentified | | | | C30HN3O10 | >0.05 | **-2.64** |
| 592.1495 | 1.00 | | Unidentified | | | | C34H20N6O5 | **1.50E-02** | <2 |
| 594.3737 | 1.93 | | Unidentified | | | | C43H48NO | **4.81E-04** | **-4.37** |
| 760.0917 | 12.24 | | Unidentified | | | | C50H12N6O4 | **7.31E-07** | **-2.06** |

Abbreviations: HILIC - hydrophilic interaction chromatography; DHF/DSS - dengue hemorrhagic syndrome/dengue shock syndrome; DF - dengue fever; ND - non-dengue febrile disease; RT - retention time; FC - fold change; DENV – dengue virus; DB – database; MSI - Metabolomics Standard Initiative; HMDB - human metabolome database; LMGP - Lipid maps gateway; METLIN - Metabolite and Tandem Mass Spectrometry Database; PC - phosphatidylcholine; SM sphingomyelin.
