# Supplementary material for: Metabolomics-Based Discovery of Small Molecule Biomarkers in Serum Associated with Dengue Virus Infections and Disease Outcomes
Source: PLoS Negl Trop Dis. 2016 Feb 25;10(2):e0004449. doi: 10.1371/journal.pntd.0004449 (PMC4768770; doi:10.1371/journal.pntd.0004449)
Supplement: S5 Table — MSI Level 3: Metabolites putatively identified based on physicochemical characteristics of a chemical class of compounds or by spectrum similarity to known compounds. MSI Level 4: Unidentified or unclassified metabolites that can be differentiated or quantified based in spectrum data. Bolded values indicate statistically significant differences in the pairwise comparison of the two immune response groups. The identities of the metabolites will be subjected to further LCMS-MS analysis to be classified at MSI levels 1 and 2. (DOCX) [file pntd.0004449.s015.docx]

**S5 Table.** **Nicaraguan acute-phase serum metabolites that differentiate dengue primary-type vs. secondary-type immune response in pairwise comparisons.** MSI Level 3: Metabolites putatively identified based on physicochemical characteristics of a chemical class of compounds or by spectrum similarity to known compounds. MSI Level 4: Unidentified or unclassified metabolites that can be differentiated or quantified based in spectrum data. Bolded values indicate statistically significant differences in the pairwise comparison of the two immune response groups. The identities of the metabolites will be subjected to further LC-MS/MS analysis to be classified at MSI levels 1 and 2.

| **MSI level 3** | | | | | | | |
| --- | --- | --- | --- | --- | --- | --- | --- |
| **Mass** | **RT** | **Potential ID** | **Calculated Formula** | **#DB hits** | **DB identifier** | **Secondary vs. primary infection** | |
|  |  |  |  |  |  | **P value** | **FC** |
| 622.3245 | 1.59 | Glu Arg Arg Tyr | C31H42N8O6 | >5 | Metlin133379 | **3.12E-03** | <2 |
| 661.4748 | 1.92 | PE(30:1) | C35H68NO8P | >5 | Metlin60269 | **2.51E-03** | <2 |
| 799.5714 | 12.17 | PS(38:3) | C44H82NO9P | >5 | Metlin78683 | **6.42E-03** | **-2.39** |
| 801.588 | 12.41 | PS(38:2) | C44H84NO9P | >5 | Metlin78663 | **2.29E-02** | **-2.14** |
| 807.5786 | 12.52 | PC(38:5) | C46H82NO8P | >5 | Metlin39385 | **8.41E-03** | >2 |
| 831.5773 | 12.01 | PC(40:7) | C48H82NO8P | >5 | Metlin39655 | **2.38E-02** | **2.05** |
| 861.5593 | 12.83 | PS(42:7) | C48H80NO10P | >5 | Metlin78221 | **3.05E-04** | <2 |
| 883.5406 | 11.98 | PS(44:10) | C50H78NO10P | >5 | Metlin76479 | **1.25E-03** | <2 |
| **MSI Level 4** | | | | | | | |
| **Mass** | **RT** | **Potential ID** | **Calculated Formula** | | | **Secondary vs. primary infection** | |
|  |  |  |  |  |  | **P value** | **FC** |
| 152.0586 | 2.98 | Unidentified | C7H8N2O2 | | | **7.31E-03** | <2 |
| 171.108 | 1.08 | Unidentified | C4H11N8 | | | **2.02E-02** | <2 |
| 323.3184 | 1.37 | Unidentified | C21H41NO | | | **2.74E-03** | **3.25** |
| 336.1364 | 1.11 | Unidentified | C21H20O4 | | | **1.26E-02** | <2 |
| 398.243 | 1.30 | Unidentified | C21H30N6O2 | | | **2.06E-02** | <2 |
| 462.3913 | 1.46 | Unidentified | C24H52N3O5 | | | **7.87E-04** | **-2.35** |
| 463.4222 | 1.40 | Unidentified | C22H53N7O3 | | | **9.07E-04** | <2 |
| 479.4175 | 1.48 | Unidentified | C25H51N8O | | | **6.63E-03** | <2 |
| 506.4178 | 1.56 | Unidentified | C27H52N7O2 | | | **3.59E-03** | **-2.33** |
| 523.444 | 1.58 | Unidentified | C27H55N8O2 | | | **2.64E-03** | **-2.00** |
| 567.4699 | 1.71 | Unidentified | C29H59N8O3 | | | **4.17E-03** | **-2.00** |
| 578.4771 | 1.66 | Unidentified | C33H62N4O4 | | | **9.25E-05** | **-2.20** |
| 579.5071 | 1.55 | Unidentified | C31H63N8O2 | | | **1.03E-02** | <2 |
| 594.4704 | 1.85 | Unidentified | C31H60N7O4 | | | **3.74E-04** | <2 |
| 611.497 | 1.87 | Unidentified | C31H63N8O4 | | | **1.12E-02** | <2 |
| 623.5331 | 1.67 | Unidentified | C33H67N8O3 | | | **1.11E-03** | <2 |
| 672.6496 | 1.29 | Unidentified | C39H84N4O4 | | | **8.34E-05** | **-2.39** |
| 699.549 | 2.32 | Unidentified | C49H69N3 | | | **1.96E-07** | **-2.83** |
| 727.5806 | 2.26 | Unidentified | C51H73N3 | | | **7.96E-03** | <2 |
| 764.831 | 1.40 | Unidentified | C46H100N8 | | | **9.15E-04** | **2.89** |
| 859.4831 | 12.09 | Unidentified | C44H63N10O8 | | | **7.74E-03** | **2.00** |
| 905.2508 | 2.10 | Unidentified | C59H33N6O5 | | | **9.99E-04** | <2 |

Abbreviations: HILIC - hydrophilic interaction chromatography; DHF/DSS - dengue hemorrhagic syndrome/dengue shock syndrome; DF - dengue fever; ND - non-dengue febrile disease; RT - retention time; FC - fold change; DB – database; MSI - Metabolomics Standard Initiative; METLIN - Metabolite and Tandem Mass Spectrometry Database; PC - phosphatidylcholine; PE – phosphatidylethanolamine; PS – phosphatidylserine.
